# Robust and General Late-Stage Methylation of Aryl Chlorides: Application to Isotopic Labeling of Drug-like Scaffolds

**DOI:** 10.1021/acscatal.3c02761

**Published:** 2023-08-16

**Authors:** Elliot Davenport, Daniela E. Negru, Geoff Badman, David M. Lindsay, William J. Kerr

**Affiliations:** † Drug Substance Development, GSK, GSK Medicines Research Centre, Gunnels Wood Road, Stevenage SG1 2NY, U.K.; ‡ Department of Pure and Applied Chemistry, University of Strathclyde, Glasgow G1 1XL, U.K.

**Keywords:** isotopic labeling, methylation, boron, cross-coupling, catalysis

## Abstract

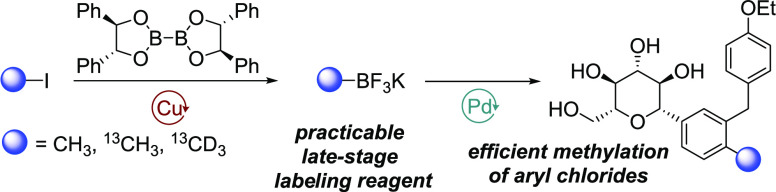

The preparation of isotopically labeled compounds for drug discovery and development presents a unique challenge. Both stable and radioactive isotopes must be incorporated into complex bioactive molecules as efficiently as possible, using precious, and often expensive, isotopically enriched reagents. Due to the ubiquity and importance of methyl groups in drug molecules, there is a requirement for a general, late-stage methylation that allows for the incorporation of both carbon and hydrogen isotopes. Herein, we report a highly efficient, robust palladium-catalyzed approach, optimized via high-throughput experimentation, for the methylation of aryl chlorides using potassium methyltrifluoroborate. A practically straightforward route to isotopically labeled methylating agents has also been developed, and the methodology applied to isotopologue synthesis, including the installation of isotopic labels in a range of drug-like scaffolds.

Isotopic labeling is a crucial process within drug discovery and development pathways.^1^ This impactful strategy delivers the installation of an analytical marker without changing the chemical structure, physical properties, or, by and large, the biological activity of the compound. Pharmaceutical compounds labeled with stable isotopes (most commonly ^2^H, ^13^C, and ^15^N) are vital tools for drug metabolism and pharmacokinetic bioanalysis, where they are employed as internal standards (SILS) for LC–MS/MS-based assays. SILS are chromatographically retained to the same extent as the analyte but have a distinct difference in the mass of their molecular ion, usually by at least 4 mass units, to avoid a cross-signal overlap.^2^ Meanwhile, radionuclides, specifically long-lived β-emitters (e.g., ^3^H and ^14^C), are routinely employed within absorption, distribution, metabolism, and excretion analyses *in vivo*;^3^ radioligand binding assays for target validation and hit identification;^4^ and environmental fate and effect studies (Figure 1a).^5^ Synthetic route design for an isotopologue tends to differ from the unlabeled molecule, as the major consideration is the efficient installation of the expensive, isotopically enriched motif. Related to this, the ideal scenario is one in which the stable or radioactive label(s) can be installed in high yield in the final step of the synthesis.

**Figure 1:**
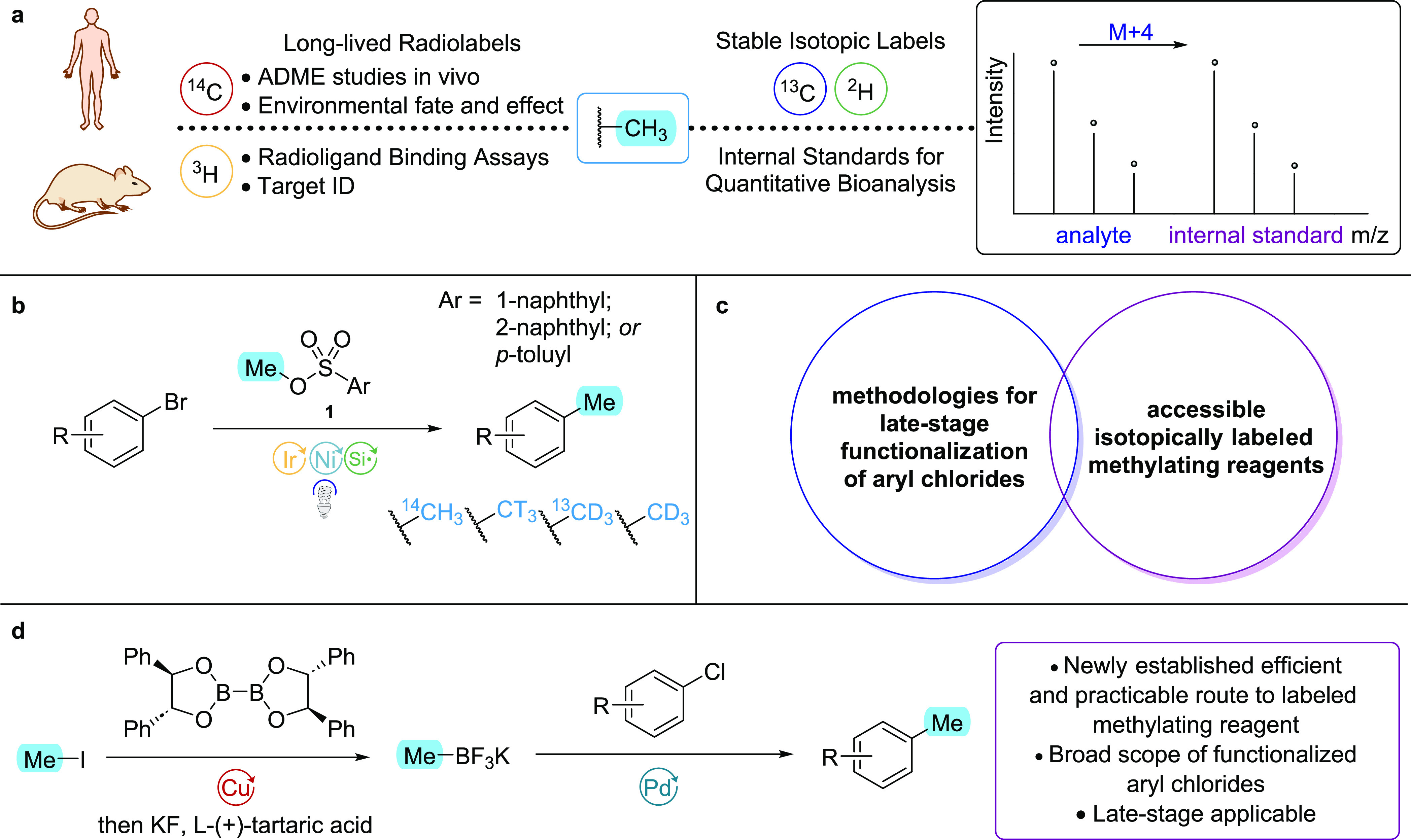
(a) Stable and radioisotopes have numerous applications in the drug discovery and development pipeline. (b) MacMillan and co-workers isotopically labeled methylation of aryl bromides. (c) Late-stage isotopic labeling of aryl chlorides with suitable methylating reagents remains a challenging transformation. (d) Our approach to late-stage isotopically labeled methylation of aryl chlorides via a robust cross-coupling strategy.

The field of hydrogen isotope exchange offers a multitude of strategies for the installation of a deuterium or tritium label in the latter stages of a synthetic route via C–H activation.^6^ In contrast, numerous applications require a carbon label, which are, commonly, more robust to chemical and metabolic cleavage. General strategies for late-stage carbon isotope introduction are more limited, and the current state of the art generally involves the introduction of labeled carboxylate or nitrile groups,^7^ including innovative carbon isotope exchange processes.^8^

Methyl groups represent arguably the most attractive motif for late-stage isotope incorporation, as they allow for both carbon and hydrogen labels to be introduced. The late-stage introduction of a methyl unit could conceivably enable the synthesis of isotopologues containing either 4 additional mass units (^13^CD_3_), a radioactive carbon label (^14^C/^11^C), or a high specific activity −CT_3_ group, all utilizing a single methodology. Moreover, as one-carbon building blocks, methylating reagents are inherently accessible from the major isotopically enriched carbon sources, such as methane, carbon monoxide, and barium carbonate. Furthermore and notably, methyl groups are highly prevalent in pharmaceutical compounds, appearing in over 63% of the 200 top-selling small-molecule drugs in 2021.^9^ Despite the appeal of this strategy, isotopically flexible and broadly applicable methodologies for the late-stage introduction of labeled methyl units within drug-like structures are extremely limited.

While the pool of C–C bond-forming methodologies is large and continues to grow, most cannot be directly applied to isotopically labeled methylation for a range of reasons. First, the methyl source must be a reagent that is commercially available as an enriched isotopologue, or which can be accessed efficiently from an available labeled precursor. Additionally, the reagent should be readily handled in a radiochemical context (i.e., nonvolatile and air-stable). Finally, the methodology should target alkylation of reactive functional handles that are likely to be present in late-stage intermediates. Indeed, this latter requirement rules out the strategy of coupling a labeled methyl electrophile with classical nucleophilic organometallic reagents (e.g., organoboron, organotin, or organozinc species).

The development of cross-electrophile coupling for Csp^2^–Csp^3^ bond formation has enabled the use of methylating reagents with desirable physical properties for use as labeling reagents, such as sulfonate ester **1**, employed by MacMillan and co-workers in a cross-coupling with aryl halides (Figure 1b).^10a^ The ease of handling associated with the reagents **1**, combined with an elegant metallaphotoredox approach, allows for the methylation of aryl bromides incorporating various isotopes of hydrogen and carbon.^10b^ However, such nickel-mediated metallaphotoredox cross-couplings are considerably less applicable when employing aryl chloride substrates.^11^ The efficient methylation of aryl and heteroaryl chlorides would be additionally attractive, given that they represent both a less reactive and more ubiquitous late-stage functionalization handle. In this regard, Doyle et al. have described an elegant Ni/photoredox-catalyzed methylation of aryl- and heteroaryl chlorides, using either trimethyl orthoformate or benzaldehyde dimethyl acetal.^12^ However, the use of this approach to access a range of methyl group isotopologues would result in appreciable and undesirable (radio)isotopic waste as part of each transformation, given the presence of two or three methyl groups in the acetal or orthoformate reagents, respectively. Based on this precedent, a general approach to the late-stage methylation of aryl chlorides, which is also compatible with isotopically labeled methyl moieties, has yet to be fully realized (Figure 1c).^13^

Herein, we outline a targeted high-throughput approach to the optimization of palladium-catalyzed methylation of aryl chlorides, using potassium methyltrifluoroborate as the methyl source. Further, we describe the development of a practical and efficient route to carbon and hydrogen isotope-labeled variants of this methylating agent, enabling access to flexibly labeled drug-like molecules via this optimized late-stage methodology (Figure 1d).

To begin to address the challenges outlined above, we proposed that potassium methyltrifluoroborate had the potential to be an ideal reagent for the isotopically labeled late-stage methylation of aryl chlorides. In particular, as an easy-to-handle, free-flowing solid,^14^ this species possesses the key properties that would allow for facile use even under the more rigorous requirements within radiochemical laboratories. In addition, organotrifluoroborate salts have emerged as versatile reagents in cross-coupling and their use for the alkylation of aryl chlorides has previously been demonstrated by Molander and co-workers.^15^ Nonetheless, only a very limited number of isolated examples have been reported for the methylation of aryl chlorides using MeBF_3_K.^15,16^ Therefore, we initially embarked on a focused, systematic study to identify a robust set of methylation conditions that could be applied to a range of aryl and heteroaryl substrates and which could also be readily employed for isotopologue synthesis of functionalized, drug-like molecules. Aligned with this, we applied a high-throughput approach to identify an optimal set of general conditions for the methylation of aryl chlorides. Four bases commonly employed in Suzuki–Miyaura reactions (cesium carbonate, potassium carbonate, potassium phosphate, and potassium *tert*-butoxide) were individually screened against a panel of 11 palladium catalysts, selected for their known reactivity profiles in cross-coupling (Scheme 1a). Three aryl chlorides (1-chloro-4-nitrobenzene **2**, 4-chloroanisole **3**, and 2-chloro-1,3-dimethylbenzene **4**) were selected as model substrates representing electron-deficient, electron-rich, and sterically encumbered aryl chlorides, respectively, and the various catalyst/base combinations were screened in parallel against all three substrates. Structurally similar pre-catalysts, SPhos Pd G3 and RuPhos Pd G3, were both found to give high yields across all three substrates when combined with potassium phosphate. A system based on SPhos Pd G3 was, thus, adopted to further probe the generality of this process.

**Scheme 1:**
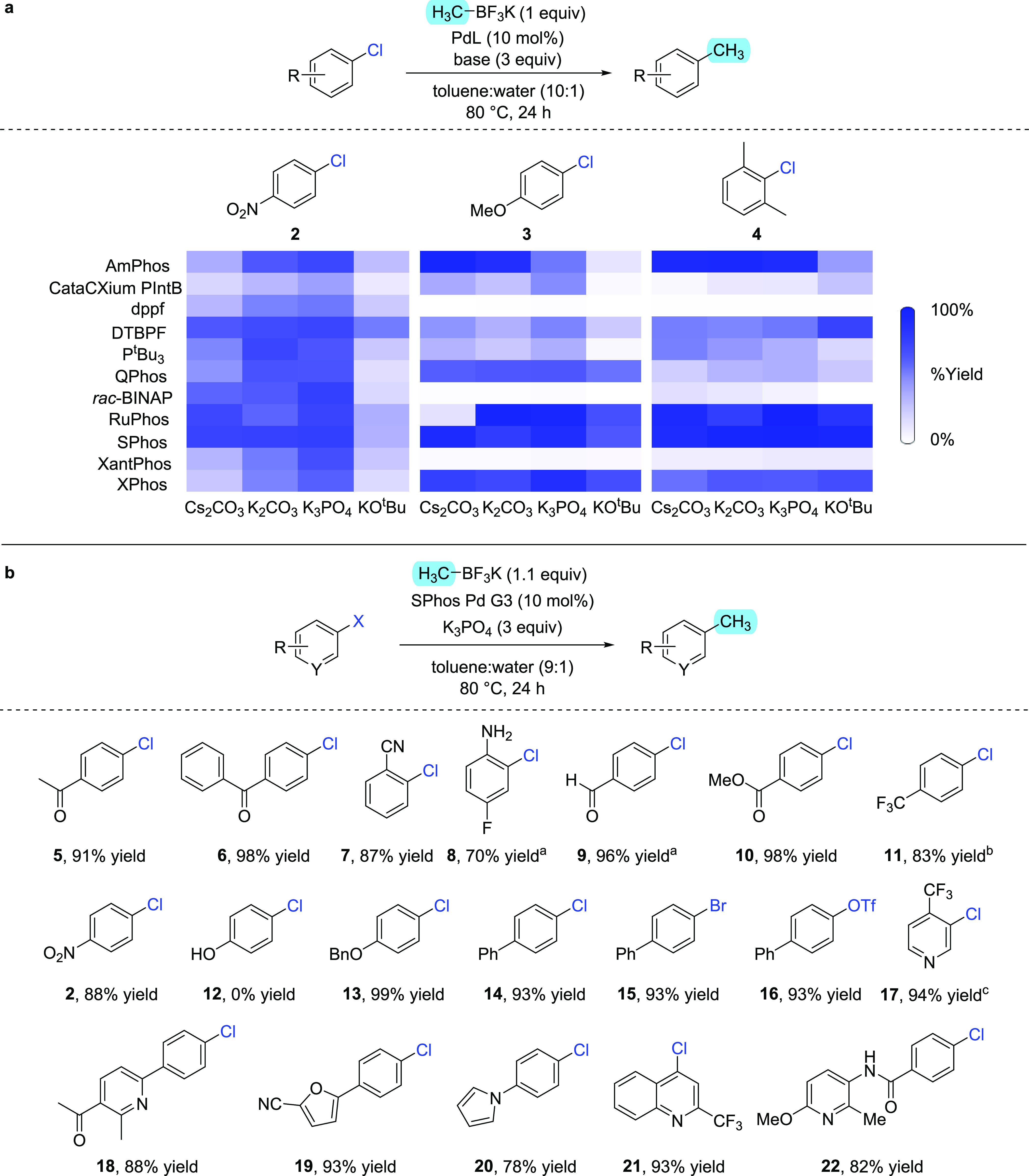
(a) Results of a High-Throughput Experiment to Identify the Most Effective Catalyst and Base Combination across Multiple Substrates; (b) Methylation of (Hetero)aryl Halides/Pseudo-Halides (0.4 mmol Scale) Yields are isolated, or determined by ^a^GC; ^b^HPLC; or ^c19^F NMR.

Using the chosen catalysis conditions, we examined the substrate scope, as detailed in Scheme 1b. Consistent with the results observed in the initial screen, both electron-deficient and electron-rich substrates reacted well under the established protocol. Ketones **5** and **6** gave the methylated products in excellent yields. Additionally, *ortho*-substituted aryl chlorides performed well, as exemplified by nitrile **7** and aniline **8**. Indeed, not only do these substituents sterically encumber the site of oxidative addition, but they also have the potential to competitively coordinate to the catalyst. Aldehyde **9** and ester **10** gave excellent yields, and strongly electron-withdrawing substituents, such as in the *para*-trifluoromethyl and *para*-nitro derivatives, **11** and **2**, respectively, also performed well, albeit with moderately reduced product yields, a trend seemingly inversely correlated to their presumed rates of oxidative addition.^17^ Unprotected phenol **12** did not react, which is likely due to phenoxide formation under the basic reaction conditions, leading to solubility issues. However, benzyl-protected analogue **13** was methylated in near quantitative yield. While the methodology was targeted specifically at aryl chlorides, we were pleased to observe that aryl bromide **15** and triflate **16** give similarly high yields of the methylated product. Substrates **18**, **19**, and **20**, possessing pyridyl, furyl, and pyrrole motifs, respectively, were well-tolerated and gave good-to-excellent yields. Meanwhile, heteroaryl chlorides, such as quinoline **21** and substituted pyridine **17**, were also converted to the methylated product in very good yields. Finally, substrate **22**, containing both pyridyl and amide units, also performed well under the established method.

To further probe the functional group compatibility of the optimized methodology, an additive screen was designed to examine the effect that 16 compounds had on the reaction (Scheme 2).^18^ The additives chosen each contained a chemical motif likely to be found in pharmaceutical compounds and synthetic intermediates thereof. The methylation of 4-chlorobiphenyl **14** was chosen as the standard reaction, and both product yield and additive stability were monitored in each case. In good alignment with the substrate scope, the ketone, ester, aldehyde, nitrile, and aniline additives were all well-tolerated under the reaction conditions and did not impede product formation. Furthermore, the presence of an alkyl chloride, a primary amine, an amide, a primary alcohol, and a phenol all still allows for high-yielding methylation, suggesting that none of these functional groups hinder the activity of the catalyst under the established conditions. A key observation from the additive screen was the incompatibility of alkynes, both terminal and internal, under the formulated method. While the desired methylation reaction still proceeded, both alkynes were also consumed, likely via coordination to the palladium center to form a π-complex, where a range of subsequent processes are then possible.^19^ A similar effect was, not unexpectedly, observed with terminal alkene **A**. In contrast, the increased stability of vinylnaphthalene **J** led to a much lower level of its consumption, and the desired cross-coupling was unaffected. Finally, the presence of 2-hydroxypyridine completely shuts down the desired catalysis process, likely through chelation of both heteroatoms. Nonetheless, methylated compounds bearing this 2-hydroxypyridine motif could still conceivably be accessed via demethylation of the corresponding 2-methoxypyridine,^20^ with a substrate possessing this latter heterocycle class having been shown to perform well within the established methylation procedure (**22**; Scheme 1).

**Scheme 2:**
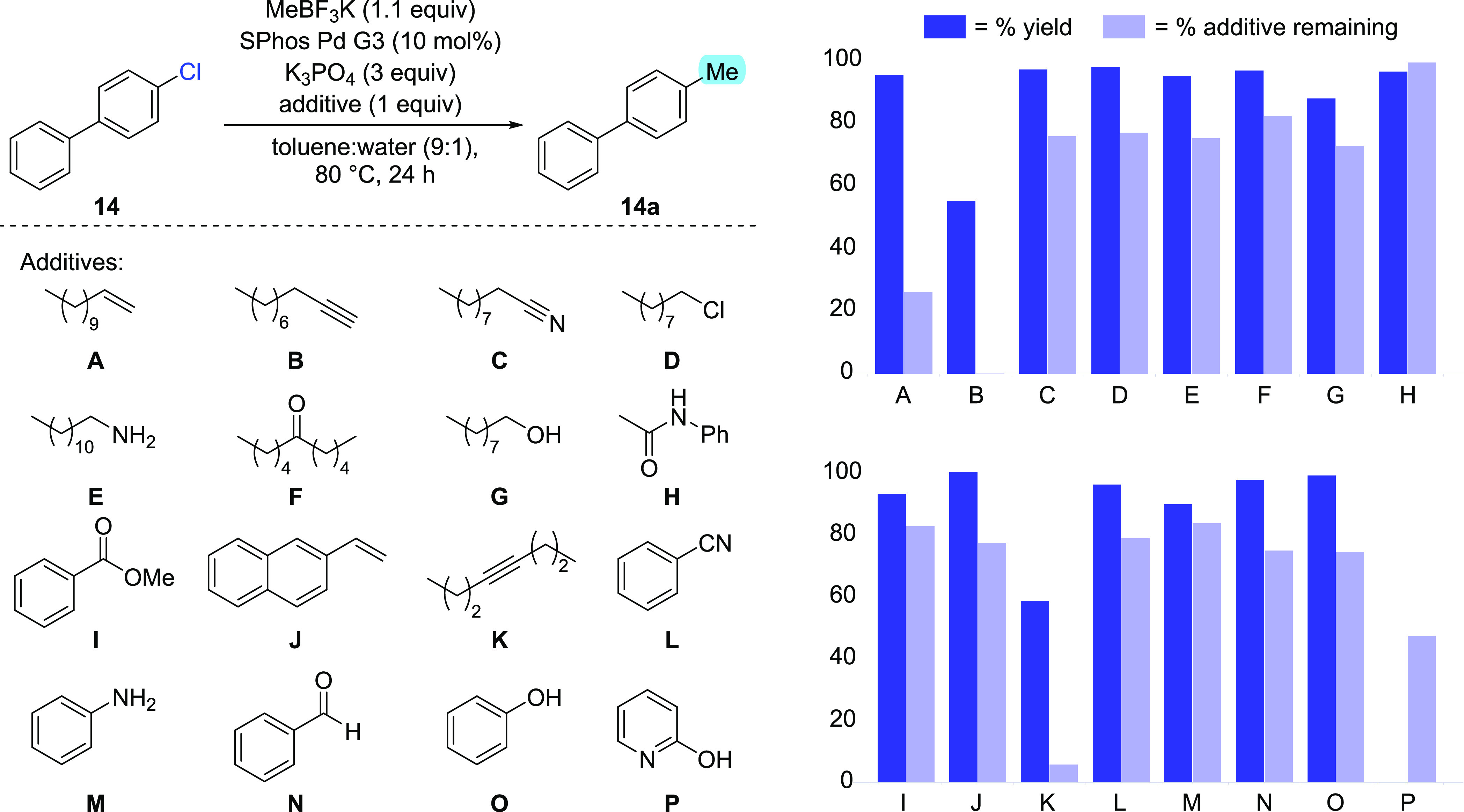
Additive Screen to Determine How Various Functional Groups Affect Reaction Yield and How the Additives Are Affected by the Reaction Conditions

Having established a general protocol for the efficient methylation of aryl chlorides with a broad array of functional group tolerance, we next focused on evaluating the methodology for use in isotopic labeling. Despite the conceptually ideal nature of MeBF_3_K as a labeled methylating agent, this species has not been widely adopted in isotope chemistry, likely due to the perception that it is not practically straightforward to access from available labeled precursors. Indeed, to the best of our knowledge, no carbon-labeled MeBF_3_K analogue has previously been reported.

Traditionally, methylboron species are accessed via electrophilic trapping of a reactive organometallic intermediate, such as methyllithium, with a boric ester.^21^ For an isotopically enriched analogue, methyllithium would likely be prepared from methyl iodide, which is not a facile transformation in the context of labeled material manipulation requirements.^22^ Compared to the direct use of methyl iodide, or readily accessible derivatives thereof, a multistep procedure involving reactive organometallic reagents to reach the desired isotopic methylating agent is strongly disfavored, particularly in a radiochemical setting where efficiency and practicability are paramount.

Given the broad and ever-expanding range of transition metal-catalyzed borylation methodologies in the literature,^23^ we sought to develop a practical and efficient direct borylation of methyl iodide with a diboron reagent to furnish a boronic ester, which could subsequently be converted to the trifluoroborate salt in a facile manner. Such a protocol would, in turn, render potassium methyltrifluoroborate a more attractive and accessible reagent for isotopic labeling. We began by exploring a copper-catalyzed process originally reported by Marder and co-workers for the borylation of alkyl bromides with B_2_pin_2_ (Scheme 3a).^24^ Applying the methodology to methyl iodide gave promising solution yields. However, the volatility of methyl–Bpin **23** made isolation challenging, as well as ruling out this approach if radiolabeled material was required. As a result, several diboron derivatives of higher-molecular-weight diols were synthesized and screened to identify a reagent that reacted as efficiently as B_2_pin_2_ but led to a less volatile boronic ester intermediate. Pleasingly, tetraphenyl-dioxaborolane **24** was readily accessed from tetrahydroxydiboron and (*R*,*R*)-hydrobenzoin. Employing this reagent in the borylation of [^13^CD_3_]-methyl iodide gave nonvolatile boronic ester **25** in good yield (Scheme 3b). The reaction workup was tailored to be rapid and practically straightforward, avoiding column chromatography of the sensitive boronate ester^25^ to maximize yield, while removing any reaction components that could hinder subsequent conversion of the boronic ester to the desired trifluoroborate salt. Fluorination of boronic ester intermediate **25** was carried out using potassium fluoride and l-(+)-tartaric acid^26^ to furnish trifluoroborate salt **26** as a colorless solid in quantitative yield.

**Scheme 3:**
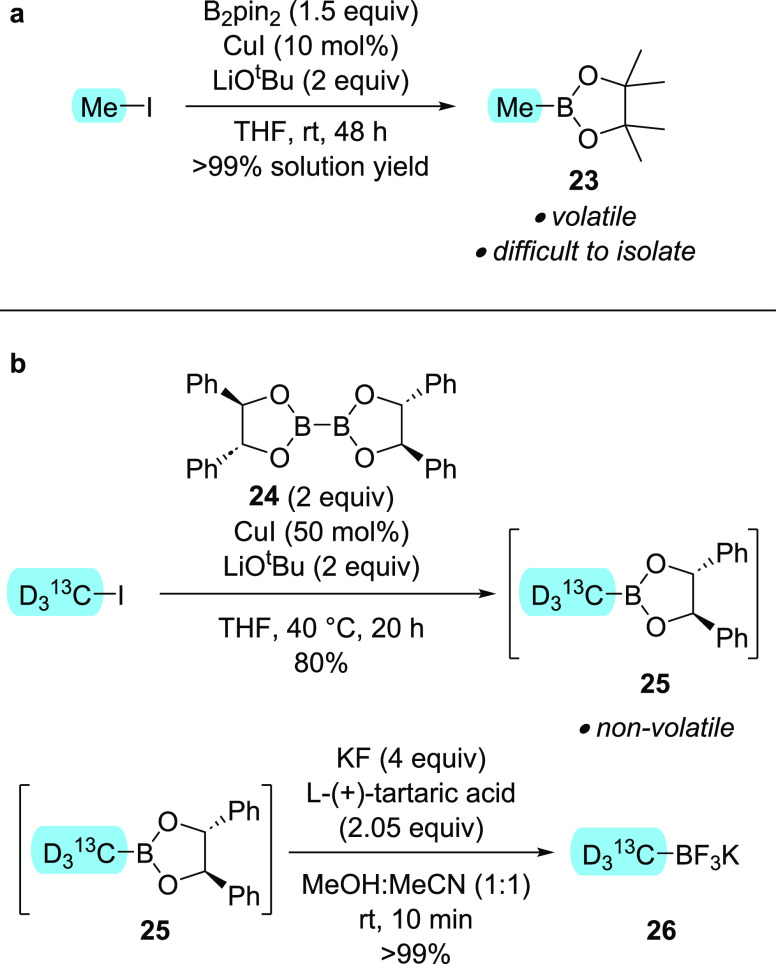
(a) Attempted Borylation of Methyl Iodide with B_2_pin_2_ Based on a Methodology Developed by Marder and Co-Workers; (b) Efficient Two-Step Route to Labeled MeBF_3_K from Methyl Iodide via a Nonvolatile Methyl Boronic Ester Intermediate

With a practical and efficient method to access carbon or hydrogen isotopologues of potassium methyltrifluoroborate in hand, the applicability of the optimized late-stage methylation procedure was demonstrated on several drug-like scaffolds (Scheme 4). The registered drugs amoxapine and chlormezanone, both of which contain an aryl chloride unit, could be methylated with [^13^C]-MeBF_3_K in an almost quantitative manner to yield **27** and **28**, respectively, demonstrating the tolerance of the reaction for common pharmaceutical motifs such as amides, sulfones, and secondary and tertiary amines. Similarly, a carbon-labeled methyl group could be installed on a late-stage precursor to lidocaine to give the ^13^C-labeled drug **29** in acceptable yield, particularly given the sterically encumbered nature of the aryl chloride substrate. Perphenazine, a drug compound containing an aryl chloride moiety, was also methylated to deliver the ^13^C-labeled derivative **30** in good yield. This result further reflects the findings of the additive screen, as the tethered primary alcohol appears not to interfere with the desired coupling process. A ^13^C-labeled derivative of celecoxib, **31**, was synthesized via final-stage methylation of the corresponding aryl chloride intermediate, with our developed process giving a synthetically useful yield in the presence of both a primary sulfonamide and a potentially coordinating heterocycle. Furthermore, and to demonstrate the power of this methodology in delivering labeled products to meet SILS requirements, [M + 4]-isotopologues **32**, **33**, and **34** were prepared in high yield from the registered drug compounds dapagliflozin, chloroquine, and haloperidol, respectively. The near quantitative methylation of dapagliflozin to yield **32** is particularly noteworthy, given that the reaction proceeds unimpeded in the presence of an unprotected sugar motif.

**Scheme 4:**
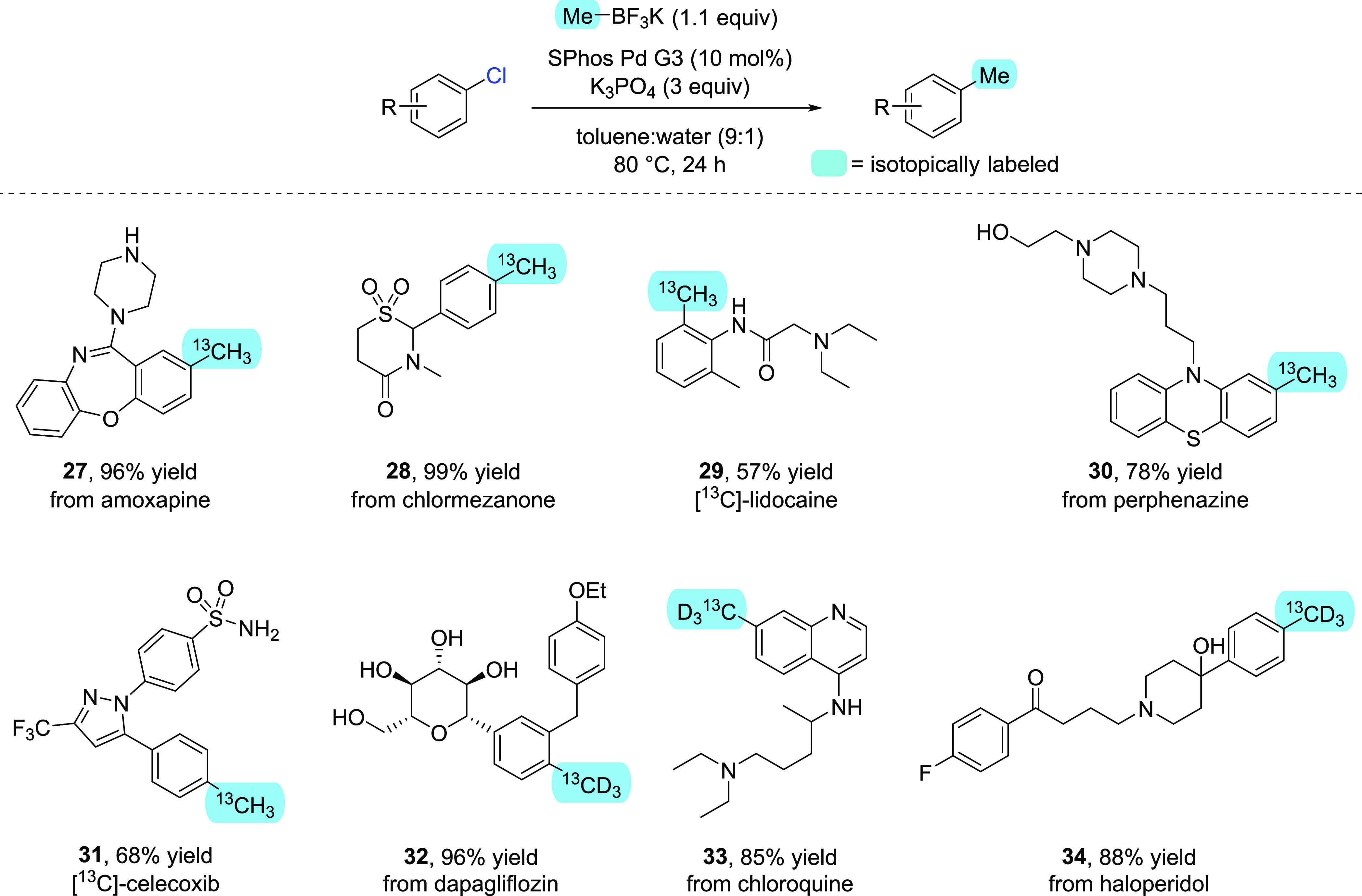
Late-Stage Methylation of Drug-like Scaffolds to Efficiently Install Isotopic Labels

In conclusion, through a focused optimization study, we have developed a high-yielding and generally applicable cross-coupling process for the methylation of aryl and heteroaryl chlorides using potassium methyltrifluoroborate. The functional group compatibility of the established protocol has been explored through both a traditional substrate scope and a related robustness screen. Subsequently, a new route to the key methylating agent has been developed to render it viable from isotopically enriched precursors, proceeding efficiently through nonvolatile intermediates, allowing its use in isotopologue synthesis. To the best of our knowledge, this represents the first reported example of a carbon-enriched MeBF_3_K isotopologue, emphasizing the previous inaccessibility of such a reagent. Finally, a number of drug-like scaffolds have been isotopically labeled via late-stage methylation, further highlighting the functional group compatibility and applicability of the established method to isotopic labeling of architectures of direct pharmaceutical interest.

## Supporting Information Available

Details of experimental procedures and analytical methods (PDF)

## Abbreviations

Bn: benzyl
Tf: triflyl
THF: tetrahydrofuran
