# Supplementary material for: Robust and General Late-Stage Methylation of Aryl Chlorides: Application to Isotopic Labeling of Drug-like Scaffolds
Source: ACS Catal. 2023 Aug 16;13(17):11541–7. doi: 10.1021/acscatal.3c02761 (PMC10476154; doi:10.1021/acscatal.3c02761)

Supporting Information:

A Robust and General Late-stage Methylation of Aryl Chlorides; Application to Isotopic Labelling of Drug-like Scaffolds

Elliot Davenport,[†‡] Daniela E. Negru,[*†] Geoff Badman,[†] David M. Lindsay,[‡] William J. Kerr[*‡]

[†] Drug Substance Development, GSK, GSK Medicines Research Centre, Gunnels Wood Road, Stevenage, SG1 2NY, U.K.

[‡] Department of Pure and Applied Chemistry, University of Strathclyde, Glasgow G1 1XL, Scotland, U.K.

*daniela.x.roman@gsk.com

*w.kerr@strath.ac.uk

Contents

1. General Experimental Information ... 3
2. Compound Characterisation Data ... 5
3. High Throughput Chemistry Protocol ... 21
4. Additive Screen Protocol ... 27
5. References ... 32
6. NMR Spectra ... 34

1. General Experimental Information

Analytical Chromatography:

Thin layer chromatography was carried out using Macherey–Nagel POLYGRAM® SIL G/UV_{254} plates and analysed using UV light and developed using potassium permanganate or vanillin dips.

Liquid chromatography–mass spectrometry (LCMS) analysis was conducted using the following system: an Acquity UPLC CSH C18 column (2.1 mm × 50 mm i.d. 1.7 µm packing diameter). Two solvent systems were used depending on the nature of the analyte:

Low pH method: 0.1% formic acid in water (solvent A) and 0.1% formic acid in acetonitrile (solvent B) eluting with the following gradient: 0.0–1.5 min 3–100% B, 1.5–1.9 min 100% B, and 1.9—2.0 min 100–3% B, at a flow rate of 1 mL/min at 40 °C;

High pH method: 10 mM ammonium bicarbonate in water, adjusted to pH 10 with ammonia solution (solvent A) and acetonitrile (solvent B) eluting with the following gradient: 0.0–1.5 min 0–97% B, 1.5–1.9 min 97% B, and 1.9–2.0 min 97–0% B, at a flow rate of 1 mL/min at 40 °C.

The UV detection was an averaged signal from a wavelength of 210–350 nm, and mass spectra were recorded on a Waters QDa mass spectrometer using alternate-scan electrospray positive and negative mode ionization (ES+ve and ES–ve).

High Pressure Liquid Chromatography (HPLC) analysis was conducted using the following system: a Phenomenex Kinetex XB-C18 100 Å column (50 mm × 3 mm i.d. 2.6 µm packing diameter) eluting with 0.05% trifluoroacetic acid in water (solvent A) and 0.05% trifluoroacetic acid in acetonitrile (solvent B), using the following elution gradient 0.0–8 min 0-95% B, at a flow rate of 1 mL/min at 40 °C. The UV detection was at 220 nm.

Preparative Chromatography:

Normal-phase Column chromatography was performed using a Biotage Isolera automatic chromatography machine and Biotage Sfär Silica Cartridges.

Reverse-phase chromatography was performed on a CombiFlash EZ Prep using an Xselect column eluting with one of the following solvent systems:

Low pH: 0.1% formic acid in water (solvent A) and 0.1% formic acid in acetonitrile (solvent B);

High pH: 10 mM ammonium bicarbonate in water, adjusted to pH 10 with ammonia solution (solvent A) and acetonitrile (solvent B).

Mass Directed Auto Purification (MDAP) was carried out on a Waters MDAP using an Xbridge Prep C18 (150 x 30 mm, 5 μm) eluting with the following solvent system:

Mobile Phase A: 10 mM ammonium carbonate in water, adjusted to pH 10 with aqueous ammonia; Mobile Phase B: Acetonitrile; Total Flow rate: 40 mL/min; 60–100% B over 20 minutes.

^{1}H, ^{13}C, ^{11}B, & ^{19}F NMR were recorded on a Bruker AV-400 spectrometer at 400, 100, 128, & 376 MHz, respectively. Chemical shifts are reported in parts per million (ppm) and are referenced to the appropriate solvent peak. Coupling constants refer to $^{3}J_{H-H}$ interactions, unless otherwise stated, and are reported in Hz.

IR spectroscopy was carried out using a PerkinElmer Spectrum Two IR.

High resolution mass spectrometry (HRMS) was carried out on one of the following systems:

A: Waters XEVO G2-XS Qtof mass spectrometer using positive electrospray ionisation with a scan range of 100 to 1200 atomic mass units (AMU).

B: Thermo Orbitrap Velos Pro mass spectrometer using either positive electrospray ionisation or positive atmospheric pressure chemical ionisation with a scan range of 100 to 1000 AMU.

C: Thermo Orbitrap Fusion mass spectrometer using positive electrospray ionisation with a scan range of 100 to 1000 atomic mass units (AMU).

Other than those species for which a synthesis is provided, all reagents were obtained from commercial suppliers and were used without additional purification unless otherwise stated.

2. Compound Characterisation Data

Synthesis of Starting Materials

1-(benzyloxy)-4-chlorobenzene (13)

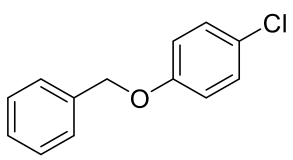

Prepared according to a literature procedure.[1]

To a stirred solution of 4-chlorophenol (317 mg, 2.47 mmol) and potassium carbonate (341 mg, 2.47 mmol) in acetonitrile (5 mL) was added benzyl bromide (401 mg, 2.34 mmol) and the resulting solution was heated to reflux for 18 hours. Aqueous sodium hydroxide (2M, 15 mL) was added, and the reaction was cooled to room temperature. Acetonitrile was removed *in vacuo* and the remaining aqueous mixture was extracted with diethyl ether (3 × 30 mL). The combined organic layers were dried over Na_2SO_4 and concentrated under reduced pressure to yield a white solid (458 mg, 85%). The obtained spectroscopic data matched literature values.[1]

^{1}H NMR (400 MHz; $CDCl_3$) δ ppm: 7.45–7.31 (5H, m), 7.26–7.22 (2H, m), 6.94–6.88 (2H, m), 5.05 (2H, s);

^{13}C NMR (100 MHz; $CDCl_3$) δ ppm: 157.4, 136.6, 129.4, 128.7, 128.1, 127.5, 125.8, 116.2, 70.3.

[1,1'-biphenyl]-4-yl trifluoromethanesulfonate (16)

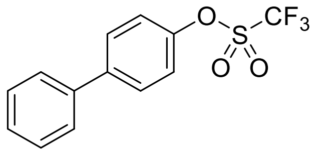

Prepared according to a literature procedure.[2]

To a solution of 4-phenylphenol (1.70 g, 10.0 mmol) in toluene (20 mL) was added aqueous potassium phosphate (tribasic) (30% w/w, 20 mL). The reaction mixture was cooled to 0 °C and triflic anhydride (3.39 g, 12 mmol) was added dropwise with stirring to maintain a reaction temperature of less than 10 °C. The reaction mixture was subsequently allowed to warm to room temperature, stirred for a further 30 min, then extracted with ethyl acetate (2 × 10 mL). The combined organic layers were washed with water, dried over $MgSO_4$, and concentrated under reduced pressure to give the crude residue. Normal phase column chromatography (50 g SFAR column), eluting with 0–20% ethyl acetate in heptane over 15 column volumes, gave the pure product as a colourless solid (2.60 g, 86%). The obtained spectroscopic data matched literature values.[2]

^{1}H NMR (400 MHz; $CDCl_3$) δ ppm: 7.69–7.64 (2H, m), 7.60–7.55 (2H, m), 7.51–7.45 (2H, m), 7.44–7.38 (1H, m), 7.37–7.34 (2H, m);

^{13}C NMR (100 MHz; $CDCl_3$) δ ppm: 148.9, 141.7, 139.3, 129.0, 128.9, 128.1, 127.2, 121.6, 118.8 (q, $^1J_{C\text{-}F}$ = 320.6 Hz);

^{19}F NMR (376 MHz; $CDCl_3$) δ ppm: −72.8.

4-chloro-*N*-(6-methoxy-2-methylpyridin-3-yl)benzamide (22)

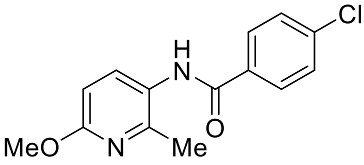

A solution of 4-chlorobenzoic acid (1.25 g, 7.98 mmol), pyridine (2.13 mL, 26.3 mmol) and 6-methoxy-2-methylpyridin-3-amine (1.21 g, 8.78 mmol) in ethyl acetate (6 mL) was cooled to 0 °C. A solution of propylphosphonic anhydride (T3P) in ethyl acetate (50% wt/wt, 9.41 mL, 15.97 mmol) was then added dropwise. The reaction mixture was warmed to room temperature and left to stir for 18 hours. The reaction mixture was then cooled to 0 °C and 0.5 M HCl (3.3 mL) was added dropwise. The mixture was warmed to room temperature and stirred for an additional 5 hours. The pink precipitate was collected by filtration and the filter cake was washed with water (3 × 10 mL). Reverse phase chromatography (High pH, 35-85% solvent B over 30 minutes) was used to further purify the product and yielded **22** as a colourless solid (1.95 g, 88%).

^{1}H NMR (400 MHz; $CDCl_3$) δ ppm: 7.86 (1H, d, *J* = 8.6 Hz), 7.81 (2H, d, *J* = 8.4 Hz), 7.48 (1H, br s), 7.47 (2H, d, *J* = 8.4 Hz), 6.62 (1H, d, *J* = 8.6 Hz), 3.92 (3H, s), 2.44 (3H, s);

^{13}C NMR (100 MHz; $CDCl_3$) δ ppm: 165.1, 161.5, 149.3, 138.5, 135.8, 132.9, 129.3, 128.7, 125.1, 108.3, 53.7, 20.7;

FT-IR ν_{max} (neat): 3189, 3003, 1641, 1474, 830 cm^{-1}.

HRMS (System A - ESI): m/z calculated for $C_{14}H_{14}{}^{35}ClN_2O_2^+$ [M+H]$^+$: 277.0744; found: 277.0755.

1,4,4,5,5-pentamethyl-1,3,2-dioxaborolane (23)

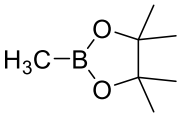

A dry microwave vial was charged with copper(I) iodide (38.0 mg, 0.20 mmol), lithium *tert*-butoxide (320 mg, 4.00 mmol), and B_2Pin_2 (762 mg, 3.00 mmol). The vial was sealed, evacuated, and backfilled thrice with nitrogen. Dry THF (4.0 mL) was added and the mixture was stirred for 5 minutes. Methyl iodide (125 µL, 2.00 mmol) was added and the mixture was stirred at room temperature for 48 h. 1,3,5-Trimethoxybenzene (81.0 mg, 0.48 mmol) was added as an internal standard and an aliquot of the mixture was analysed directly by ^{1}H NMR (S37). The internal standard showed a >99% solution yield. The obtained spectroscopic data matched literature values.[3]

(4*R*,4′*R*,5*R*,5′*R*)-4,4′,5,5′-tetraphenyl-2,2′-bi(1,3,2-dioxaborolane) (24)

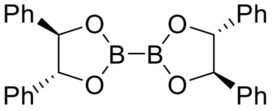

A dry round bottom flask was charged with tetrahydroxydiboron (1.36 g, 15.2 mmol), (*R*,*R*)-(+)-hydrobenzoin (6.50 g, 30.3 mmol), and oven-dried 4 Å molecular sieves. Dry THF (80 mL) was added, and the mixture was stirred at 70 °C for 18 h. After 18 h, the reaction mixture was cooled to room temperature and filtered through a pad of Celite, washing with dichloromethane. The filtrate was concentrated under reduced pressure to yield an off-white solid. Residual starting material was removed by triturating the solid in methanol (10 mL) and filtering to yield **24** as a white solid (5.88 g, 13.2 mmol, 87%). The obtained spectroscopic data matched literature values.[4]

^{1}H NMR (400 MHz; $CDCl_3$) δ ppm: 7.44–7.34 (m, 20H), 5.28 (s, 4H);

^{13}C NMR (100 MHz; $CDCl_3$) δ ppm: 139.8, 128.8, 128.4, 126.0, 86.6;

^{11}**B NMR** (128 MHz; $CDCl_3$) δ ppm: 31.8.

(4*R*,5*R*)-2-(methyl-^{13}C)-4,5-diphenyl-1,3,2-dioxaborolane (25a)

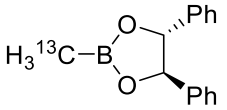

A dry round bottom flask equipped with a large stirrer bar was charged with B_2(hydrobenzoin)$_2$ **24** (3.10 g, 6.95 mmol), copper(I) iodide (381 mg, 2.00 mmol), and lithium *tert*-butoxide (640 mg, 8.00 mmol). The flask was sealed with a septum cap, and evacuated and backfilled thrice with nitrogen. Dry THF (40 mL) was then added, and the reaction mixture was stirred for 5 minutes. [^{13}C]-Methyl iodide (572 mg, 4 mmol) was then added and the reaction was stirred at 40 °C. After 20 h, the reaction mixture was filtered through Celite, washing with EtOAc (3 x 10 mL). Silica was added directly to the filtrate and the mixture was concentrated under reduced pressure until dry, free flowing silica remained. The impregnated silica was then loaded on to a short silica plug (10 g) and washed with 9:1 heptane:ethyl acetate mixture (400 mL). The filtrate was concentrated under reduced pressure to yield a sticky white crude solid which was used without further purification (1.03 g, 95% pure, 98% yield).

^{1}**H NMR** (400 MHz; $CDCl_3$) δ ppm: 7.43–7.30 (10H, m), 5.17 (2H, s), 0.55 (3H, d, $^{1}J_{C\text{-}H}$ = 117.9 Hz).

(4*R*,5*R*)-2-(methyl-^{13}C-*d*$_3$)-4,5-diphenyl-1,3,2-dioxaborolane (25)

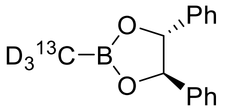

A dry round bottom flask equipped with a large stirrer bar was charged with **24** (3.57 g, 8.00 mmol), copper(I) iodide (381 mg, 2.00 mmol), and lithium *tert*-butoxide (640 mg, 8.00 mmol). The flask was sealed with a septum cap, and evacuated and backfilled thrice with nitrogen. Dry THF (40 mL) was then added, and the reaction mixture was stirred for 5 min. [$^{13}CD_3$]-Methyl iodide (584 mg, 4 mmol) was then added and the reaction was stirred at 40 °C. After 18 h, the reaction mixture was filtered through Celite, washing with EtOAc (3 x 10 mL). Silica was added directly to the filtrate and the mixture was concentrated under reduced pressure until dry, free flowing silica remained. The impregnated silica was then loaded on to a short silica plug (10 g) and washed with 9:1 heptane:ethyl acetate mixture (400 mL). The filtrate was concentrated under reduced pressure to yield a sticky white crude solid which was used without further purification (774 mg - estimated based on NMR integrals, 80%).

^{1}**H NMR** (400 MHz; $CDCl_3$) δ ppm: 7.45–7.29 (10H, m), 5.17 (2H, s);

^{11}**B NMR** (128 MHz; $CDCl_3$) δ ppm: 35.1.

Potassium (methyl-^{13}C)trifluoroborate (26a)

$H_3{}^{13}C{-}BF_3K$

Prepared according to a modified literature procedure.[5]

To **25a** (239 mg, 1.00 mmol) was added methanol (2 mL) and acetonitrile (2 mL). A solution of potassium fluoride (232 mg, 4.00 mmol) in water (0.4 mL) was then added. The mixture was stirred for 1 min. L-(+)-tartaric acid (308 mg, 2.05 mmol) was dissolved in THF (1.5 mL) (heating and agitation required for complete dissolution) and the solution was added dropwise to the rapidly stirring reaction mixture over a period of 5 minutes, as a white precipitate formed.

The reaction was stirred for 2 min, diluted with acetonitrile (3 mL), and stirred for a further 2 min before being diluted again with acetonitrile (1 mL) and filtered. The flask and filter cake were rinsed with further portions of acetonitrile (3 × 5 mL) and the combined filtrates were concentrated under reduced pressure to give a colourless solid. The crude solid was transferred to a glass-sintered funnel and washed with diethyl ether (3 × 15 mL) to remove the hydrobenzoin by-product. The remaining solids were dried under vacuum to yield a colourless solid (123 mg, >99%)

^{1}H NMR (400 MHz; DMSO-*d*$_6$) δ ppm: −0.59 (3H, d, $^{1}J_{C\text{-}H}$ = 111.0 Hz);

^{13}C NMR (100 MHz; DMSO-*d*$_6$) δ ppm: 4.0 (1C, qq, $^{1}J_{C\text{-}B}$ = 71.7, $^{2}J_{C\text{-}F}$ = 50.4 Hz);

^{11}B NMR (128 MHz; DMSO-*d*$_6$) δ ppm: 4.8 (1B, app quint, $^{1}J_{B\text{-}F}$ = 64.6 Hz);

^{19}F NMR (376 MHz; DMSO-*d*$_6$) δ ppm: −130.7–−131.5 (3F, m).

Potassium (methyl-^{13}C-*d*$_3$)trifluoroborate (26)

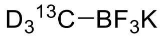

Prepared according to a modified literature procedure.[5]

To **25** (774 mg, 3.20 mmol) was added methanol (8 mL) and acetonitrile (8 mL). A solution of potassium fluoride (743 mg, 12.8 mmol) in water (1 mL) was then added. The mixture was stirred for 2 min. L-(+)-tartaric acid (984 mg, 6.55 mmol) was dissolved in THF (6 mL) (heating and agitation required for complete dissolution) and the solution was added dropwise to the rapidly stirring reaction mixture over a period of 5 minutes, as a white precipitate formed. The reaction was stirred for 2 min, diluted with acetonitrile (8 mL), and stirred for a further 2 min before being diluted again with acetonitrile (2.5 mL) and filtered. The flask and filter cake were rinsed with further portions of acetonitrile (3 × 5 mL) and the combined filtrates were concentrated under reduced pressure to give a colourless solid. The crude solid was transferred to a glass-sintered funnel and washed with diethyl ether (3 × 15 mL) to remove the hydrobenzoin by-product. The remaining solids were dried under vacuum to yield a colourless solid (405 mg, >99%)

^{13}C NMR (100 MHz; DMSO-*d*$_6$) δ ppm: 5.0–1.5 (1C, m);

^{11}B NMR (128 MHz; DMSO-*d*$_6$) δ ppm: 4.8 (1B, app quint, $^{1}J_{B\text{-}F}$ = 62.6 Hz);

^{19}F NMR (376 MHz; DMSO-*d*$_6$) δ ppm: −130.8–−131.5 (3F, m).

1 mmol-Scale Preparation of Potassium Trifluoroborate

The methylating reagent can be prepared on single mmol scale suitable for radiolabelling applications, as described below for the unlabelled species:

(4*R*,5*R*)-3-methyl-4,5-diphenyl-1,3,2-dioxaborolane

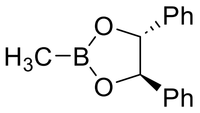

A dry 20 mL microwave vial equipped with a stirrer bar was charged with B_2(hydrobenzoin)$_2$ (892 mg, 2.00 mmol), copper(I) iodide (95.0 mg, 0.50 mmol), and lithium *tert*-butoxide (240 mg, 3.00 mmol). The vial was sealed with a septum cap, and evacuated and backfilled thrice with nitrogen. Dry THF (10 mL) was then added, and the reaction mixture was stirred for 5 minutes. Methyl iodide (62.0 µL, 1.00 mmol) was then added and the reaction was stirred at 40 °C. After 20 h, the

reaction mixture was filtered through a Celite, washing with EtOAc. The filtrate was washed with an aqueous 20% NH_4Cl solution (3 × 20 mL) before being dried over $MgSO_4$, filtered, and concentrated to yield the crude residue, which was purified via column chromatography, eluting with 0–10% EtOAc in heptane over 14 column volumes. Concentrating the desired fractions yielded the product as a colourless oil (140 mg, 59%).

^{1}H NMR (400 MHz; $CDCl_3$) δ ppm: 7.42–7.27 (m, 10H), 5.15 (s, 2H), 0.54 (s, 3H);

^{13}C NMR (100 MHz; $CDCl_3$) δ ppm: 140.5, 128.9, 128.5, 125.9, 86.5 (Methyl peak broadened by B, not assigned);

^{11}B NMR (128 MHz; $CDCl_3$) δ ppm: 35.1.

Potassium methyltrifluoroborate

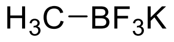

A 10 mL RBF was charged with (4*R*,5*R*)-3-methyl-4,5-diphenyl-1,3,2-dioxaborolane (238 mg, 1.00 mmol). Methanol (2 mL) and acetonitrile (2 mL) were added, followed by potassium fluoride (232 mg, 4.00 mmol) in water (0.4 mL). The mixture was stirred for 1 min. L-(+)-tartaric acid (308 mg, 2.05 mmol) was dissolved into THF (1.5 mL) (gentle heat and agitation was required for rapid dissolution) and added drop-wise to the reaction mixture over a 5 min period, as a white precipitate formed. The reaction was stirred for 2 min, diluted with acetonitrile (3 mL), and stirred for a further 2 min before being diluted again with acetonitrile (1 mL) and filtered. The flask and filter cake were rinsed with further portions of acetonitrile (3 × 5 mL) and the filtrate was concentrated under reduced pressure to give a white solid. The crude solid was triturated with diethyl ether to remove (*R*,*R*)-(+)-hydrobenzoin, leaving the product as a white solid (120 mg, 98%).

^{1}H NMR (400 MHz; D_2O) δ ppm: −0.29 (3H, br s);

^{13}C NMR (100 MHz; D_2O) δ ppm: −2.0 (1C, br s);

^{11}B NMR (128 MHz; D_2O) δ ppm: 6.2 (1B, q, $^1J_{B\text{-}F}$ = 63.9 Hz);

^{19}F NMR (376 MHz; D_2O) δ ppm: −131.9 (3F, q, $^1J_{B\text{-}F}$ = 64.1 Hz).

4-(5-(4-chlorophenyl)-3-(trifluoromethyl)-1H-pyrazol-1-yl)benzenesulfonamide (31b)

Prepared according to a literature procedure.[6]

Stage one: Preparation of (*Z*)-4-(4-chlorophenyl)-1,1,1-trifluoro-4-hydroxybut-3-en-2-one (31a)

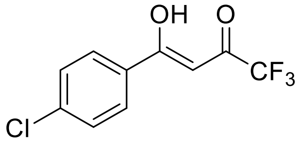

To a suspension of sodium hydride (931 mg, 23.3 mmol) in THF (20 mL) was added ethyl-2,2,2-trifluoroacetate (6.62 g, 46.6 mmol). After stirring at rt for 10 min, the mixture was cooled to 0 °C and a solution of 4-chloroacetophenone (3.00 g, 19.4 mmol) in THF (20 mL) was added dropwise. This mixture was heated under reflux for 2 h before being cooled to rt and poured on to ice cold 1M HCl (50 mL). Ethyl acetate (30 mL) was added, and the organic phase was washed with saturated aqueous sodium bicarbonate (30 mL), then brine (30 mL). The organic phase was then dried over sodium sulfate and concentrated under reduced pressure. The crude residue was filtered through a short pad of silica, eluting with ethyl acetate before once again being concentrated to yield an orange solid. The product was recrystallised (hexane) to yield an off-white solid (3.80 g, 78%).

^{1}**H NMR** (400 MHz; $CDCl_3$) δ ppm: 15.04 (1H, br s), 7.87 (2H, d, *J* = 8.6 Hz), 7.47 (2H, d, *J* = 8.6 Hz), 6.51 (1H, s);

^{13}**C NMR** (100 MHz; $CDCl_3$) δ ppm: 185.3, 177.3 (q, $^{2}J_{C\text{-}F}$ = 35.6 Hz), 140.7, 131.7, 129.5, 129.1, 117.3 (q, $^{1}J_{C\text{-}F}$ = 283.8 Hz), 92.4;

^{19}**F NMR** (376 MHz; $CDCl_3$) δ ppm: −76.5.

Stage 2: Preparation of 4-(5-(4-chlorophenyl)-3-(trifluoromethyl)-1H-pyrazol-1-yl)benzenesulfonamide (31b).

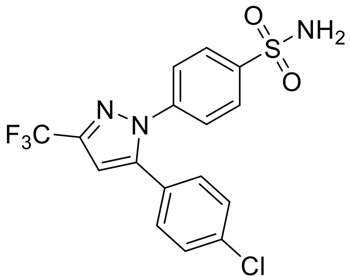

2-(4-Sulfamoylphenyl)hydrazinium chloride (982 mg, 4.39 mmol) was added to a stirred solution of (*Z*)-4-(4-chlorophenyl-1,1,1-trifluoro-4-hydroxybut-3-en-2-one (**31a**) (1.00 g, 3.99 mmol) in ethanol (50 mL). The mixture was heated under reflux for 36 h. After cooling to room temperature, the reaction mixture was concentrated under reduced pressure. The residue was taken up in ethyl acetate (20 mL), washed with water (20 mL), and then with brine (20 mL), before being dried over magnesium sulfate and concentrated under reduced pressure to yield a crude orange residue. Reverse phase chromatography (low pH, 35-85% solvent B over 30 minutes) yielded the pure product as a colourless solid (1.11 g, 69%). The obtained spectroscopic data matched literature values.[6]

^{1}**H NMR** (400 MHz; DMSO-d_6) δ ppm: 7.91–7.86 (2H, m), 7.57–7.54 (2H, m), 7.53–7.48 (4H, m), 7.37–7.33 (2H, m), 7.28 (1H, s);

^{13}**C NMR** (100 MHz; DMSO-d_6) δ ppm: 144.1, 144.0, 142.2 (q, $^{2}J_{C\text{-}F}$ = 38.2 Hz), 140.8, 134.3, 130.8, 128.9, 127.1, 126.9, 126.0, 121.2 (q, $^{1}J_{C\text{-}F}$ = 268.6 Hz), 106.7;

^{19}**F NMR** (376 MHz; DMSO-d_6) δ ppm: −60.9;

HRMS (System C - ESI): m/z calculated for $C_{16}H_{12}{}^{35}ClF_3N_3O_2S^+$ $[M+H]^+$: 402.0285; found: 402.0282.

General procedure for the unlabelled methylation of aryl chlorides

A microwave vial equipped with a stirrer bar was charged with the aryl chloride substrate (0.40 mmol), potassium methyltrifluoroborate (54 mg, 0.44 mmol, 1.1 equiv), SPhos Pd G3 (31 mg, 0.04 mmol, 0.1 equiv), and potassium phosphate (255 mg, 1.20 mmol, 3.0 equiv). The vial was sealed with a teflon-coated septum cap and evacuated, then refilled, thrice with nitrogen. Toluene (1.8 mL) and water (0.2 mL) (both degassed by sparging for 15 min with nitrogen) were then added and the reaction was stirred at 80 °C.

General workup procedure for non-volatile compounds

After 24 h, the mixture was cooled to rt and filtered into a separatory funnel where it was diluted with ethyl acetate (10 mL) and water (10 mL). The organic layer was washed with brine (10 mL) before the combined aqueous layers were extracted with ethyl acetate (10 mL). The combined organic layers were dried over magnesium or sodium sulfate, filtered, and concentrated under reduced pressure to yield the crude residue. The crude was purified by normal phase column chromatography.

1-methyl-4-nitrobenzene (2a)

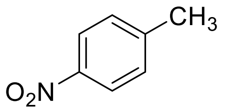

Synthesised and worked up according to the general procedures from 4-chloronitrobenzene (63.0 mg, 0.40 mmol). The crude was purified using a 10 g SFAR silica cartridge, eluting with 0–10% ethyl acetate in heptane over 15 column volumes. Concentrating the desired fractions yielded the product as a colourless oil (48 mg, 88%). The obtained spectroscopic data matched literature values.[7]

^{1}H NMR (400 MHz; $CDCl_3$) δ ppm: 8.11 (2H, d, *J* = 8.3 Hz), 7.31 (2H, d, *J* = 8.8 Hz), 2.46 (3H, s);

^{13}C NMR (100 MHz; $CDCl_3$) δ ppm: 146.3, 146.1, 129.9, 123.7, 21.8;

FT-IR ν_{max} (neat): 3038, 1926, 1795, 1509, 1343, 1320 cm^{-1}.

1-(*p*-tolyl)ethan-1-one (5a)

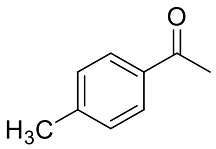

Synthesised and worked up according to the general procedures from 4-chloroacetophenone (62 mg, 0.4 mmol). The crude was purified using a 10 g SFAR silica cartridge, eluting with 0–10% ethyl acetate in heptane over 15 column volumes. Concentrating the desired fraction yielded the product as a colourless oil (49 mg, 91%). The obtained spectroscopic data matched literature values.[8]

^{1}H NMR (400 MHz; $CDCl_3$) δ ppm: 7.87 (2H, d, *J* = 8.1 Hz), 7.27 (2H, d, *J* = 7.9 Hz), 2.58 (3H, s), 2.42 (3H, s);

^{13}C NMR (100 MHz; $CDCl_3$) δ ppm: 197.9, 144.0, 134.8, 129.3, 128.5, 26.6, 21.7;

IR ν_{max} (neat): 1677, 1406, 904, 724 cm^{-1}.

phenyl(*p*-tolyl)methanone (6a)

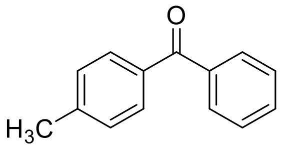

Synthesised and worked up according to the general procedures from 4-chlorobenzophenone (87 mg, 0.4 mmol). The crude was purified using a 10 g SFAR silica cartridge, eluting with 0–10% ethyl acetate in heptane over 15 column volumes. Concentrating the desired fractions yielded the product as a white solid (76 mg, 98%). The obtained spectroscopic data matched literature values.[9]

^{1}H NMR (400 MHz; $CDCl_3$) δ ppm: 7.80–7.76 (2H, m), 7.74–7.70 (2H, m), 7.57 (1H, tt, *J* = 6.6, 1.2 Hz), 7.50–7.44 (2H, m), 7.28 (2H, d, *J* = 7.9 Hz), 2.44 (3H, s);

^{13}C NMR (100 MHz; $CDCl_3$) δ ppm: 196.6, 143.4, 138.1, 135.0, 132.3, 130.4, 130.0, 129.1, 128.3, 21.8;

FT-IR ν_{max} (neat): 1652, 1606, 903, 723 cm^{-1}.

2-methylbenzonitrile (7a)

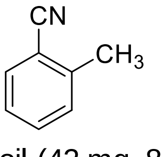

Synthesised and worked up according to the general procedures from 2-chlorobenzonitrile (55 mg, 0.4 mmol). The crude was purified using a 10 g SFAR silica cartridge, eluting with 0–15% ethyl acetate in heptane over 20 column volumes. Concentrating the desired fractions yielded the product as a colourless oil (42 mg, 87%). The obtained spectroscopic data matched literature values.[10]

^{1}H NMR (400 MHz; $CDCl_3$) δ ppm: 7.59 (1H, dd, *J* = 7.6, 1.2 Hz), 7.47 (1H, td, *J* = 7.6, 1.2 Hz), 7.33–7.23 (2H, m), 2.54 (3H, s);

^{13}C NMR (100 MHz; $CDCl_3$) δ ppm: 142.1, 132.7, 132.6, 130.3, 126.3, 118.3, 112.9, 20.6;

FT-IR ν_{max} (neat): 2981, 2226, 760, 712 cm^{-1}.

4-fluoro-2-methylaniline hydrochloride (8a·HCl)

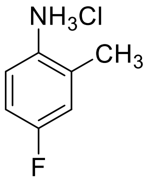

Synthesised according to the general procedure from 2-chloro-4-fluoroaniline (58 mg, 0.4 mmol). An internal standard (1,3,5-trimethoxybenzene, 31.6 mg, 0.2 mmol) was also added for quantification, due to the volatility of the title compound (70% GC yield). The volatile title compound was obtained for characterisation as the hydrochloride salt **8a·HCl** as follows: The reaction mixture was filtered and acidified with aqueous HCl (2M, 5 mL). The aqueous layer was separated and adjusted to pH 14 with NaOH (2M). The organic precipitate was then extracted with diethyl ether (5 mL) and HCl in ether (1M, 5 mL) was added. The white hydrochloride salt which precipitated was collected by filtration and washed with diethyl ether (2 × 5 mL), before being dried under vacuum. The combination of two 0.4 mmol scale reactions gave 87.5 mg of product (68% isolated yield).

^{1}H NMR (400 MHz; DMSO-d_6) δ ppm: 10.37 (2H, br s), 7.53 (1H, dd, *J* = 8.7, 5.3 Hz), 7.22 (1H, dd, *J* = 9.4, 2.7 Hz), 7.13 (1H, td, *J* = 8.5, 3.0 Hz), 2.38 (3H, s);

^{13}C NMR (100 MHz; DMSO-d_6) δ ppm: 160.9 (d, $^1J_{C-F}$ = 244.1 Hz), 135.9 (d, $^3J_{C-F}$ = 7.6 Hz), 127.2 (d, $^4J_{C-F}$ = 3.0 Hz), 125.1 (d, $^3J_{C-F}$ = 9.2 Hz), 117.8 (d, $^2J_{C-F}$ = 22.8 Hz), 113.5 (d, $^2J_{C-F}$ = 22.9), 17.2;

^{19}**F NMR** (376 MHz; DMSO-d_6) δ ppm: −114.7;

FT-IR v_{max} (neat): 2935, 1611, 1500, 1224 cm^{-1}.

4-methylbenzaldehyde (9a)

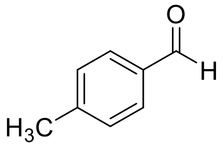

Synthesised according to the general procedure from 4-chlorobenzaldehyde (56 mg, 0.40 mmol). An internal standard (1,3,5-trimethoxybenzene, 28.0 mg, 0.20 mmol) was also added for quantification, due to the volatility of the title compound (96% GC yield). The crude was purified using a 10 g SFAR silica cartridge, eluting with 0–10% ethyl acetate in heptane to yield the title compound as a colourless oil (average of 41.5 mg, 86%, over two runs).

^{1}**H NMR** (400 MHz; $CDCl_3$) δ ppm: 9.94 (1H, s), 7.75 (2H, d, J = 8.1 Hz), 7.31 (2H, d, J = 7.9 Hz), 2.41 (3H, s);

^{13}**C NMR** (100 MHz; $CDCl_3$) δ ppm: 192.0, 145.6, 134.3, 129.9, 129.8, 21.9;

FT-IR v_{max} (neat): 2823, 1701, 806 cm^{-1}.

methyl 4-methylbenzoate (10a)

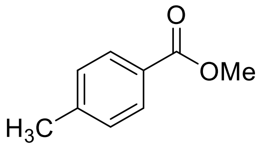

Synthesised and worked up according to the general procedures from methyl 4-chlorobenzoate (68.0 mg, 0.40 mmol). The crude was purified using a 10 g SFAR silica cartridge, eluting with 0–10% ethyl acetate in heptane over 15 column volumes. Concentrating the desired fractions yielded the product as a colourless oil (59 mg, 98%). The obtained spectroscopic data matched literature values.[11]

^{1}**H NMR** (400 MHz; $CDCl_3$) δ ppm: 7.94 (2H, d, J = 8.1 Hz), 7.25 (2H, d, J = 7.9), 3.91 (3H, s), 2.42 (3H, s);

^{13}**C NMR** (100 MHz; $CDCl_3$) δ ppm: 167.3, 143.6, 129.7, 129.2, 127.6, 52.0, 21.7;

FT-IR v_{max} (neat): 3020, 1713, 1215, 744 cm^{-1}.

1-methyl-4-(trifluoromethyl)benzene (11a)

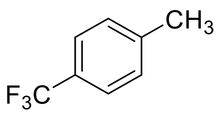

Synthesised according to the general procedure from 4-chlorobenzotrilfuoride (53.0 μL, 0.40 mmol). An internal standard (1,3,5-trimethoxybenzene, 32.7 mg, 0.19 mmol) was also added for quantification, due to the volatility of the title compound (83% HPLC yield). An aliquot of the reaction mixture was diluted with $CDCl_3$ and a ^{19}F NMR spectrum was recorded. The obtained spectroscopic data matched literature values.[12]

^{19}**F NMR** (376 MHz; $CDCl_3$) δ ppm: −62.1.

1-(benzyloxy)-4-methylbenzene (13a)

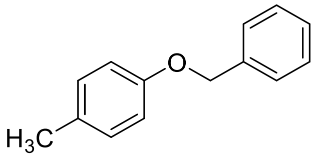

Synthesised and worked up according to the general procedures from 1-(benzyloxy)-4-chlorobenzene (**13**) (87.0 mg, 0.40 mmol). The crude was purified using a 10 g SFAR silica cartridge, eluting with 0–10% ethyl acetate in heptane over 15 column volumes. Concentrating the desired fractions yielded the product as a colourless solid (79 mg, 99%). The obtained spectroscopic data matched literature values.[13]

^{1}H NMR (400 MHz; $CDCl_3$) δ ppm: 7.45–41 (2H, m), 7.38 (2H, t, *J* = 7.3 Hz), 7.34–7.29 (1H, m), 7.10 (2H, d, *J* = 8.1 Hz), 6.90 (2H, d, *J* = 8.4 Hz), 5.06 (2H, s), 2.31 (3H, s);

^{13}C NMR (100 MHz; $CDCl_3$) δ ppm: 156.9, 137.4, 130.3, 130.0, 128.7, 128.0, 127.6, 114.9, 70.3, 20.6;

FT-IR ν_{max} (neat): 2981, 1237, 903, 723 cm^{-1}.

4-methyl-1,1′-biphenyl (14a)

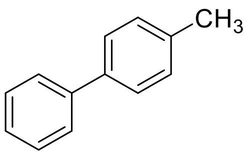

Synthesised and worked up according to the general procedures from either 4-chlorobiphenyl (**14**) (75.0 mg, 0.40 mmol), 4-bromobiphenyl (**15**) (93.0 mg, 0.40 mmol), or [1,1'-biphenyl]-4-yl trifluoromethanesulfonate (**16**) (121.0 mg, 0.40 mmol). The crude was purified using a 10 g SFAR silica cartridge, eluting with heptane over 15 column volumes. Concentrating the desired fractions yielded the product as a colourless solid (62 (93%), 62 (93%), and 62 mg (93%), respectively). The obtained spectroscopic data matched literature values.[14]

^{1}H NMR (400 MHz; $CDCl_3$) δ ppm: 7.62–7.57 (2H, m), 7.51 (2H, d, *J* = 7.9 Hz), 7.44 (2H, t, *J* = 7.8 Hz), 7.37–7.31 (1H, m), 7.27 (2H, d, *J* = 7.9 Hz), 2.42 (3H, s);

^{13}C NMR (100 MHz; $CDCl_3$) δ ppm: 141.2, 138.4, 137.0, 129.5, 128.73, 128.68, 126.98, 126.95, 21.1;

FT-IR ν_{max} (neat): 3029, 1488, 823, 754 cm^{-1}.

3-methyl-4-(trifluoromethyl)pyridine (17a)

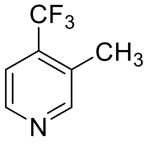

Synthesised according to the general procedure from 3-chloro-4-(trifluoromethyl)pyridine (73 mg, 0.40 mmol). At the end of the reaction, 4-fluorobenzonitrile (61.6 mg, 0.51 mmol) was added as an internal standard and the yield of the volatile title compound was quantified by ^{19}F NMR (94% NMR yield). Product was isolated (for characterisation) by adding aqueous HCl (2M, 8 mL) to the reaction mixture as well as EtOAc (8 mL). The aqueous layer was collected and adjusted to pH 14 with aqueous NaOH (2M, 12 mL), before being extracted with dichloromethane (3 mL). The dichloromethane solution was loaded on to a 10 g SFAR silica cartridge and eluted with 0-30% diethyl ether in pentane over 20 column volumes. The desired product was isolated but excess solvent was not completely removed due to the volatility of the compound.

^{1}H NMR (400 MHz; $CDCl_3$) δ ppm: 8.62–8.58 (2H, m), 7.46 (1H, d, *J* = 4.9 Hz), 2.48 (3H, s);

^{13}**C NMR** (100 MHz; $CDCl_3$) δ ppm: 153.0, 148.3, 136.6 (q, $^{2}J_{C-F}$ = 32.0 Hz), 130.8, 123.4 (q, $^{1}J_{C-F}$ = 273.1 Hz), 119.3 (q, $^{3}J_{C-F}$ = 6.1 Hz), 16.3;

^{19}**F NMR** (376 MHz; $CDCl_3$) δ ppm: −64.0.

1-(2-methyl-6-(*p*-tolyl)pyridin-3-yl)ethan-1-one (18a)

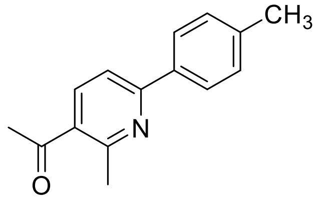

Synthesised and worked up according to the general procedures from 1-(6-(4-chlorophenyl)-2-methylpyridin-3-yl)ethanone (98.0 mg, 0.40 mmol). The crude was purified using a 10 g SFAR silica cartridge, eluting with 0–15% ethyl acetate in heptane over 15 column volumes. Concentrating the desired fractions yielded the product as a colourless solid (79 mg, 88%). The obtained spectroscopic data matched literature values.[15]

^{1}**H NMR** (400 MHz; $CDCl_3$) δ ppm: 8.04 (1H, d, *J* = 8.4 Hz), 7.98 (2H, d, *J* = 8.1 Hz), 7.63 (1H, d, *J* = 7.9 Hz), 7.30 (2H, d, *J* = 7.9 Hz), 2.85 (3H, s), 2.62 (3H, s), 2.43 (3H, s);

^{13}**C NMR** (100 MHz; $CDCl_3$) δ ppm: 199.9, 158.6, 158.5, 139.9, 137.9, 135.6, 130.4, 129.5, 127.2, 116.8, 29.3, 25.4, 21.3;

FT-IR ν_{max} (neat): 3063, 2991, 2921, 1677, 1429, 819 cm^{-1}.

5-(*p*-tolyl)furan-2-carbonitrile (19a)

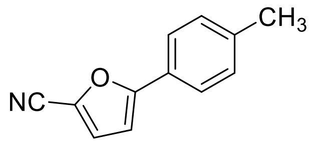

Synthesised and worked up according to the general procedures from 5-(4-chlorophenyl)furan-2-carbonitrile (81.0 mg, 0.40 mmol). The crude was purified using a 10 g SFAR silica cartridge, eluting with 0–10% ethyl acetate in heptane over 15 column volumes. Concentrating the desired fractions yielded the product as a light blue solid (68 mg, 93%). The obtained spectroscopic data matched the literature values.[12]

^{1}**H NMR** (400 MHz; $CDCl_3$) δ ppm: 7.62 (2H, d, *J* = 8.1 Hz), 7.26 (2H, d, *J* = 7.9 Hz), 7.16 (1H, d, *J* = 3.7 Hz), 6.67 (1H, d *J* = 3.7 Hz), 2.40 (3H, s);

^{13}**C NMR** (100 MHz; $CDCl_3$) δ ppm: 159.1, 139.9, 129.8, 126.2, 124.9, 124.8, 124.1, 112.2, 105.5, 21.6;

FT-IR ν_{max} (neat): 3138, 1609, 1589, 1486, 792, 752 cm^{-1}.

1-(*p*-tolyl)-1*H*-pyrrole (20a)

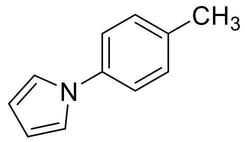

Synthesised and worked up according to the general procedures from 1-(4-chlorophenyl)-1*H*-pyrrole (71.0 mg, 0.40 mmol). The crude was purified using a 10 g SFAR silica cartridge, eluting with 0–10% ethyl acetate in heptane over 15 column volumes. Concentrating the desired fractions yielded the product as a colourless solid (49 mg, 78%). The obtained spectroscopic data matched the literature values.[12]

^{1}**H NMR** (400 MHz; $CDCl_3$) δ ppm: 7.32–7.26 (2H, m), 7.25–7.20 (2H, m), 7.07 (2H, t, *J* = 2.1 Hz), 6.34 (2H, t, *J* = 2.1 Hz), 2.39 (3H, s);

^{13}C NMR (100 MHz; $CDCl_3$) δ ppm: 138.5, 135.4, 130.0, 120.5, 119.4, 110.0, 20.8;

FT-IR ν_{max} (neat): 3147, 2918, 1523, 813, 712 cm^{-1}.

4-methyl-2-(trifluoromethyl)quinoline (21a)

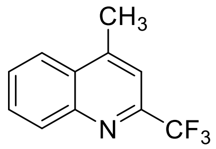

Synthesised and worked up according to the general procedures from 4-chloro-2-(trifluoromethyl)quinoline (93.0 mg, 0.40 mmol). The crude was purified using a 10 g SFAR silica cartridge, eluting with 0–15% ethyl acetate in heptane over 15 column volumes. Concentrating the desired fractions yielded the product as a colourless solid (79 mg, 93%). The obtained spectroscopic data matched the literature values.[12]

^{1}H NMR (400 MHz; $CDCl_3$) δ ppm: 8.22 (1H, d, J = 8.6 Hz), 8.06 (1H, d, J = 8.6 Hz), 7.83–7.78 (1H, m), 7.71–7.66 (1H, m), 7.58 (1H, s), 2.80 (3H, s);

^{13}C NMR (100 MHz; $CDCl_3$) δ ppm: 147.8 (q, $^{2}J_{C\text{-}F}$ = 33.6 Hz), 147.2, 147.1, 130.9, 130.5, 129.0, 128.5, 123.9, 121.8 (q, $^{1}J_{C\text{-}F}$ = 274.7 Hz), 117.6, 19.2;

^{19}F NMR (376 MHz; $CDCl_3$) δ ppm: −67.7;

FT-IR ν_{max} (neat): 2927, 1388, 1247, 1183, 1097, 912, 766 cm^{-1}.

N-(6-methoxy-2-methylpyridin-3-yl)-4-methylbenzamide (22a)

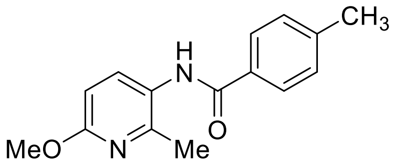

Synthesised and worked up according to the general procedures from **22** (111.0 mg, 0.40 mmol). The crude was purified using a 25 g SFAR silica cartridge, eluting with 0–40% ethyl acetate in heptane over 16 column volumes. Concentrating the desired fractions yielded the product as a colourless solid (84 mg, 82%).

^{1}H NMR (400 MHz; $CDCl_3$) δ ppm: 7.92 (1H, d, J = 8.6 Hz), 7.77 (2H, d, J = 8.1 Hz), 7.47 (1H, br s), 7.29 (2H, d J = 8.1 Hz), 6.62 (1H, d, J = 8.6 Hz), 3.92 (3H, s), 2.45 (3H, s), 2.43 (3H, s);

^{13}C NMR (100 MHz; $CDCl_3$) δ ppm: 166.0, 161.2, 148.9, 142.7, 135.7, 131.8, 129.6, 127.2, 125.6, 108.2, 53.7, 21.7, 20.7;

FT-IR ν_{max} (neat): 3269, 2994, 1639, 1496, 905 cm^{-1};

HRMS (System A - ESI): m/z calculated for $C_{15}H_{17}N_2O_2^+$ $[M+H]^+$: 257.1290; found: 257.1302.

General procedure for the methylation of drug-like aryl chlorides with labelled Me-BF_3K.

A microwave vial equipped with a stirrer bar was charged with the aryl chloride substrate (0.20 mmol), labelled potassium methyltrifluoroborate (0.22 mmol, 1.1 equiv), SPhos Pd G3 (16.0 mg, 0.02 mmol, 0.1 equiv), and potassium phosphate (127 mg, 0.60 mmol, 3.0 equiv). The vial was sealed with a teflon-coated septum cap and evacuated, then refilled, thrice with nitrogen. Toluene (0.9 mL) and water (0.1 mL) (both degassed by sparging for 15 min with nitrogen) were then added and the reaction was stirred at 80 °C. After 24 h, the mixture was cooled to rt and filtered into a separatory funnel where it was diluted with ethyl acetate (5 mL) and water (5 mL). The organic layer was washed with brine (10 mL) before the combined aqueous layers were extracted with ethyl acetate (10 mL). The combined organic layers were dried over magnesium sulfate, filtered, and concentrated under reduced pressure to yield the crude residue. The crude was purified by either normal or reverse phase column chromatography.

2-(methyl-^{13}C)-11-(piperazin-1-yl)dibenzo[b,f][1,4]oxazepine (27)

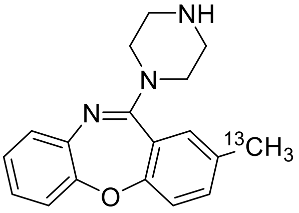

Synthesised according to the general procedure from amoxapine (63.0 mg, 0.20 mmol) and **26a** (27.0 mg, 0.22 mmol). The crude was purified using a using reverse phase chromatography (high pH), eluting with 30–85% acetonitrile in water. Concentrating the desired fractions yielded the product as a colourless solid (57 mg, 96%).

^{1}H NMR (400 MHz; $CDCl_3$) δ ppm: 7.24–7.18 (1H, m), 7.15–7.11 (3H, m), 7.09 (1H, dd, J = 7.9, 1.5 Hz), 7.04 (1H, td, J = 7.5, 1.5 Hz), 6.94 (1H, td, J = 7.6, 1.0 Hz), 3.51 (4H, br s), 2.98 (4H, br s), 2.30 (3H, d, $^1J_{C\text{-}H}$ = 126.8 Hz).

^{13}C NMR (100 MHz; $CDCl_3$) δ ppm: 160.9, 159.1, 152.5, 140.8, 134.5 (d, $^1J_{C\text{-}C}$ = 44.3 Hz), 133.3, 129.7, 127.0, 125.5, 124.2, 123.4, 121.0, 120.2, 48.9, 46.2, 21.0;

HRMS (System A - ESI): m/z calculated for $C_{17}{}^{13}CH_{20}N_3O^+$ [M+H]$^+$: 295.1634; found: 295.1639.

3-methyl-2-(4-(methyl-^{13}C)phenyl)-1,3-thiazinan-4-one 1,1-dioxide (28)

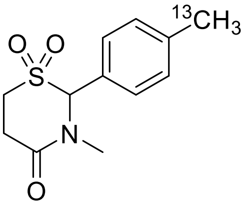

Synthesised according to the general procedure from chlormezanone (55.0 mg, 0.20 mmol) and **26a** (27.0 mg, 0.22 mmol). The crude was purified by normal phase chromatography using a 10 g SFAR silica cartridge, eluting with 40–85% ethyl acetate in heptane over 15 column volumes. Concentrating the desired fractions yielded the product as a colourless solid (51 mg, 99%).

^{1}H NMR (400 MHz; $CDCl_3$) δ ppm: 7.32–7.28 (4H, m), 5.24 (1H, d, $^4J_{H\text{-}H}$ = 2.0 Hz), 3.41–3.30 (1H, m), 3.25–3.06 (3H, m), 2.97 (3H, s), 2.41 (3H, d, $^1J_{C\text{-}H}$ = 127.0 Hz);

^{13}C NMR (100 MHz; $CDCl_3$) δ ppm: 166.2, 140.8 (d, $^1J_{C\text{-}C}$ = 44.3 Hz), 130.0, 128.0, 127.0, 80.6, 43.2, 36.2, 30.6, 21.2;

HRMS (System C - ESI): m/z calculated for $C_{11}{}^{13}CH_{16}NO_3S^+$ $[M+H]^+$: 255.0879; found: 255.0878.

[^{13}C]-lidocaine (29)

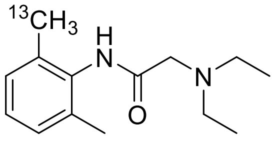

Synthesised according to the general procedure from the hydrochloride salt of *N*-(2-chloro-6-methylphenyl)-2-(diethylamino)acetamide (58.0 mg, 0.20 mmol) and **26a** (27.0 mg, 0.22 mmol). The crude was purified using reverse phase chromatography (high pH), eluting with 25–85% acetonitrile in water. Concentrating the desired fractions yielded the product as a colourless solid (27 mg, 57%).

^{1}H NMR (400 MHz; $CDCl_3$) δ ppm: 8.92 (1H, br s), 7.12–7.06 (3H, m), 3.23 (2H, s), 2.70 (4H, q, J = 7.1 Hz), 2.24 (3H, d, $^1J_{C\text{-}H}$ = 127.0 Hz), 2.24 (3H, s), 1.15 (6H, t, J = 7.1 Hz);

^{13}C NMR (100 MHz; $CDCl_3$) δ ppm: 170.4, 135.24, 135.22 (d, $^1J_{C\text{-}C}$ = 44.3 Hz), 135.21, 128.4, 127.2 (d, $^2J_{C\text{-}C}$ = 4.6 Hz), 57.7, 49.1, 18.7, 12.8;

HRMS (System A - ESI): m/z calculated for $C_{13}{}^{13}CH_{23}N_2O^+$ $[M+H]^+$: 236.1838; found: 236.1840.

2-(4-(3-(2-(methyl-^{13}C)-10*H*-phenothiazin-10-yl)propyl)piperazin-1-yl)ethan-1-ol (30)

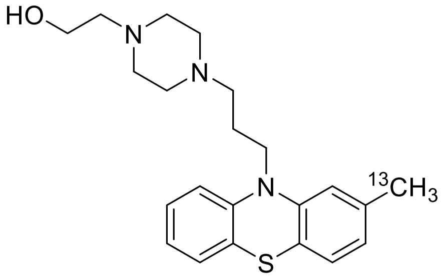

Synthesised according to the general procedure from perphenazine (81.0 mg, 0.20 mmol) and **26a** (27.0 mg, 0.22 mmol). The crude was purified using reverse phase chromatography (high pH), eluting with 35–95% acetonitrile in water. Concentrating the desired fractions yielded the product as a colourless solid (60 mg, 78%).

^{1}H NMR (400 MHz; $CDCl_3$) δ ppm: 7.16–7.10 (2H, m), 7.01 (1H, d, J = 7.9 Hz), 6.92–6.86 (2H, m), 6.76–6.69 (2H, m), 3.92 (2H, t, J = 6.9 Hz), 3.60 (2H, t, J = 5.4 Hz), 2.56–2.40 (12H, m), 2.30 (3H, d, $^1J_{C\text{-}H}$ = 126.5 Hz), 1.96 (2H, app quint, J = 7.0 Hz);

^{13}C NMR (100 MHz; $CDCl_3$) δ ppm: 145.3, 145.2, 137.1 (d, $^1J_{C\text{-}C}$ = 44.3 Hz), 127.4, 127.1, 127.0, 125.5, 123.1, 122.3, 121.7, 116.5, 115.5, 59.2, 57.7, 55.7, 53.4, 52.9, 45.2, 24.5, 21.4;

HRMS (System C - ESI): m/z calculated for $C_{21}{}^{13}CH_{30}N_3OS^+$ $[M+H]^+$: 385.2138; found: 385.2138.

[^{13}C]-celecoxib (31)

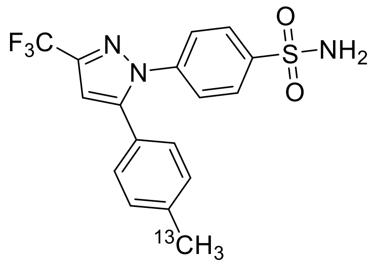

Synthesised according to the general procedure from 4-(5-(4-chlorophenyl)-3-(trifluoromethyl)-1*H*-pyrazol-1-yl) benzenesulfonamide (**31b**) (80.0 mg, 0.20 mmol) and **26a** (27.0 mg, 0.22 mmol). The crude was purified using reverse phase chromatography (low pH), eluting with 35–85% acetonitrile in water. Concentrating the desired fractions yielded the product as a colourless solid (52 mg, 68%).

^{1}H NMR (400 MHz; $CDCl_3$) δ ppm: 7.92–7.87 (2H, m), 7.49–7.44 (2H, m), 7.20–7.15 (2H, m), 7.13–7.08 (2H, m), 6.75 (1H, s), 4.92 (2H, br s), 2.39 (3H, d, $^1J_{C\text{-}H}$ = 127.0 Hz);

^{13}C NMR (100 MHz; $CDCl_3$) δ ppm: 145.4, 144.3 (q, $^2J_{C\text{-}F}$ = 38.2 Hz), 142.8, 141.4, 140.0 (d, $^1J_{C\text{-}C}$ = 44.3 Hz), 129.9, 128.9, 127.7, 125.9, 125.7, 121.2 (q, $^1J_{C\text{-}F}$ = 270.1 Hz), 106.5, 21.5;

^{19}F NMR (376 MHz; $CDCl_3$) δ ppm: −62.5;

HRMS (System C - ESI): m/z calculated for $C_{16}{}^{13}CH_{15}F_3N_3O_2S^+$ [M+H]$^+$: 383.0865; found: 383.0865.

(2*S*,3*R*,4*R*,5*S*,6*R*)-2-(3-(4-ethoxybenzyl)-4-(methyl-^{13}C-d_3)phenyl)-6-(hydroxymethyl)tetrahydro-2H-pyran-3,4,5-triol (32)

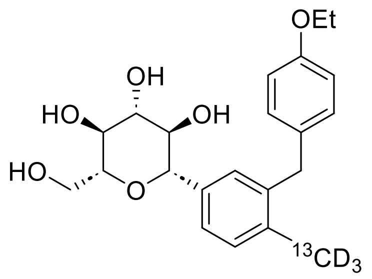

Synthesised according to the general procedure from dapagliflozin (82.0 mg, 0.20 mmol) and **26** (28.0 mg, 0.22 mmol). The crude was purified using reverse phase chromatography (high pH), eluting with 20–60% acetonitrile in water. Concentrating the desired fractions yielded the product as a colourless solid (74 mg, 96%).

^{1}H NMR (400 MHz; DMSO-d_6) δ ppm: 7.13–7.05 (3H, m), 7.02 (2H, d, *J* = 8.6 Hz), 6.81 (2H, d, *J* = 8.6 Hz), 4.89 (2H, dd, *J* = 4.9, 1.7 Hz), 4.69 (1H, d, *J* = 5.7 Hz), 4.41 (1H, t, *J* = 5.8 Hz), 3.96 (2H, q, *J* = 7.0 Hz), 3.94 (1H, d, *J* = 9.4 Hz), 3.86 (2H, d, *J* = 3.7 Hz), 3.69 (1H, ddd, *J* = 11.8, 5.5, 1.7 Hz), 3.43 (1H, dt, *J* = 11.8, 5.9 Hz), 3.29–3.12 (4H, m), 1.29 (3H, t, *J* = 7.0 Hz);

^{13}C NMR (100 MHz; CD_3OD) δ ppm: 158.7, 140.5, 138.5, 137.5 (d, $^1J_{C\text{-}C}$ = 44.3 Hz), 133.9, 131.2, 131.0, 130.8, 127.1, 115.5, 83.8, 82.3, 80.0, 76.5, 72.2, 64.6, 63.4, 39.8, 18.9 (spt, $^1J_{C\text{-}D}$ = 38.1 Hz), 15.4;

HRMS (System B - APCI): m/z calculated for $C_{21}{}^{13}CH_{25}D_3O_6^+$ [M]$^{\bullet+}$:392.2102; found: 392.2094.

*N*1,*N*1-diethyl-*N*4-(7-(methyl-^{13}C-d_3)quinolin-4-yl)pentane-1,4-diamine (33)

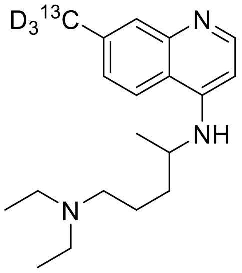

Synthesised according to the general procedure from chloroquine diphosphate (103 mg, 0.20 mmol) and **26** (28.0 mg, 0.22 mmol). The crude was purified using reverse phase chromatography (high pH), eluting with 20–70% acetonitrile in water. Concentrating the desired fractions yielded the product as a colourless solid (52 mg, 85%).

^{1}H NMR (400 MHz; $CDCl_3$) δ ppm: 8.49 (1H, d *J* = 5.4 Hz), 7.73 (1H, dd, *J* = 5.2, 1.7 Hz), 7.60 (1H, d, *J* = 8.6 Hz), 7.23 (1H, ddd, *J* = 8.5, 3.9, 1.9 Hz), 6.37 (1H, d, *J* = 5.4 Hz), 5.02 (1H, br d, *J* = 6.9 Hz), 3.71 (1H, app spt, *J* = 12.9, 6.3 Hz), 2.51 (4H, q, *J* = 7.1 Hz), 2.44 (2H, t, *J* = 6.9 Hz), 1.77–1.54 (4H, m), 1.31 (3H, d, *J* = 6.4 Hz), 1.00 (6H, t, *J* = 7.1 Hz);

^{13}C NMR (100 MHz; $CDCl_3$) δ ppm: 151.2, 149.03 (d, $^2J_{C\text{-}C}$ = 4.6 Hz), 148.98, 139.0 (d, $^1J_{C\text{-}C}$ = 42.7 Hz), 129.3, 126.5 (d, $^2J_{C\text{-}C}$ = 3.1 Hz), 119.3, 119.2, 116.9, 98.7, 52.8, 48.3, 47.0. 34.9, 24.0, 20.9 (spt, $^1J_{C\text{-}D}$ = 38.6 Hz), 11.7;

HRMS (System B - ESI): m/z calculated for $C_{18}{}^{13}CH_{27}D_3N_3^+$ [M+H]$^+$: 304.2656; found: 304.2648.

[$^{13}CD_3$]-methylhaloperidol (34)

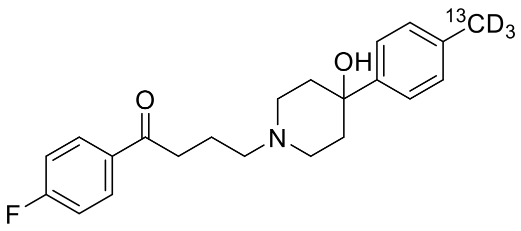

Synthesised according to the general procedure from haloperidol (75.0 mg, 0.20 mmol) and **26** (28.0 mg, 0.22 mmol). The crude was purified using reverse phase chromatography (high pH), eluting with 30–60% acetonitrile in water. Concentrating the desired fractions yielded the product as a colourless solid (63 mg, 88%).

^{1}H NMR (400 MHz; $CDCl_3$) δ ppm: 8.04–7.98 (2H, m), 7.34 (2H, d, *J* = 8.1 Hz), 7.18–7.09 (4H, m), 2.98 (2H, t, *J* = 7.1 Hz), 2.77 (2H, br d, *J* = 11.3 Hz), 2.51–2.38 (4H, m), 2.07–1.94 (4H, m), 1.70 (2H, dd, *J* = 14.2, 2.6 Hz), 1.49 (1H, br s);

^{13}C NMR (100 MHz; $CDCl_3$) δ ppm: 198.6, 165.8 (d, $^1J_{C\text{-}F}$ = 254.8 Hz), 145.6, 136.6 (d, $^1J_{C\text{-}C}$ = 44.3 Hz), 133.9 (d, $^4J_{C\text{-}F}$ = 3.1 Hz), 130.8 (d, $^2J_{C\text{-}F}$ = 9.2 Hz), 129.1 (d, $^3J_{C\text{-}C}$ = 3.1 Hz), 124.6 (d, $^2J_{C\text{-}C}$ = 4.6 Hz), 115.7 (d, $^2J_{C\text{-}F}$ = 22.9 Hz), 71.3, 58.1, 49.7, 38.6, 36.5, 22.2, 20.3 (spt, $^1J_{C\text{-}D}$ = 18.3 Hz);

^{19}F NMR (376 MHz; $CDCl_3$) δ ppm: −105.8;

HRMS (System B - ESI): m/z calculated for $C_{21}{}^{13}CH_{24}D_3FNO_2^+$ [M+H]$^+$: 360.2242; found: 360.2227.

3. High Throughput Chemistry Protocol

In triplicate, 48-well plates containing 2 mL crimp top vials were set up in a glove-box as follows:

		Catalyst					
		RuPhos Pd G3	AmPhos Pd G3	*rac*-BINAP + $Pd(OAc)_2$	dppf $PdCl_2$	SPhos Pd G3	XPhos Pd G3
Base	K_2CO_3	A1	B1	C1	D1	E1	F1
	Cs_2CO_3	A2	B2	C2	D2	E2	F2
	K_3PO_4	A3	B3	C3	D3	E3	F3
	KO^tBu	A4	B4	C4	D4	E4	F4
Base	K_2CO_3	A5	B5	C5	D5	E5	Blank
	Cs_2CO_3	A6	B6	C6	D6	E6	Blank
	K_3PO_4	A7	B7	C7	D7	E7	Blank
	KO^tBu	A8	B8	C8	D8	E8	Blank
		QPhos Pd G3	XantPhos Pd G3	CataCXium PintB + $Pd(OAc)_2$	DTBPF Pd G3	$PtBu_3$ Pd G4	Blank
		Catalyst					

All solids were weighed into the vials using a CHRONECT Quantos automatic powder dosing robot in the following quantities:

Compound	Wells	Mass (mg)	mmol
Potassium carbonate	A1–F1, A5–E5	33.2	0.24
Caesium carbonate	A2–F2, A6–E6	78.2	0.24
Potassium phosphate	A3–F3, A7–E7	50.9	0.24
Potassium *tert*-butoxide	A4–F4, A8–E8	26.9	0.24
RuPhos Pd G3	A1–A4	6.69	0.008
AmPhos Pd G3	B1–B4	5.08	0.008
rac-BINAP	C1–C4	4.98	0.016
dppf $PdCl_2$	D1–D4	5.85	0.008
SPhos Pd G3	E1–E4	6.24	0.008
XPhos Pd G3	F1–F4	6.77	0.008
QPhos Pd G3	A5–A8	8.64	0.008
XantPhos Pd G3	B5–B8	7.59	0.008
CataCXium PIntB	C5–C8	5.40	0.016
DTBPF Pd G3	D5–D8	6.75	0.008
P^tBu_3 Pd G4	E5–E8	4.69	0.008
Palladium(II) acetate	C1–C8	1.80	0.008
Potassium methyltrifluoroborate	A1–F4 & A5-E8 (all wells)	9.75	0.080

Stock solutions of all three substrates combined with internal standard were prepared as follows:

Stock solution A: 1-chloro-4-nitrobenzene (867 mg, 5.50 mmol) and 1,3,5-trimethoxybenzene (185 mg, 1.10 mmol) were dissolved in toluene (25 mL).

Stock solution B: 1-chloro-4-methoxybenzene (784 mg, 5.50 mmol) and 1,3,5-trimethoxybenzene (185 mg, 1.10 mmol) were dissolved in toluene (25 mL).

Stock solution C: 2-chloro-1,3-dimethylbenzene (773 mg, 5.50 mmol) and 1,3,5-trimethoxybenzene (185 mg, 1.10 mmol) were dissolved in toluene (25 mL).

Stock solution A (363.6 μL) was charged to each vial in the first 48-well plate, while the same quantity of stock solutions B and C was dispensed to each vial of the two corresponding plates. To every vial in all three plates was then added water (36.4 μL). The vials were sealed with crimp tops and the plates were placed on shaker plates, where they were shaken vigorously at 80 °C for 24 h. The plates were allowed to cool to room temperature before the crimp-top lids were removed from the vials and a 10:1 mixture of acetonitrile:water (800 μL) was added to each vial. 50 μL aliquots were taken from the bulk organic phase of each reaction vial and diluted with a 10:1 acetonitrile:water mixture (800 μL). All of the samples were then analysed by HPLC using the following method:

Instrument: Agilent 1290.

Column: Waters Aquity UPLC CSH C18, 1.7 micron, 2.1 mm × 100 mm.

Column temperature: 40 °C.

Injection volume: 2 μL.

Flow rate: 0.6 mL/min.

UV detection wavelength: 220 nm.

Mobile phase A: 0.05% TFA in water; Mobile phase B: 0.05% TFA in acetonitrile.

Gradient profile:

Time (mins)	% Mobile Phase B
0	5
15	95
15.1	5
17	5

For quantification, response factors (K_x values) were calculated from reference solutions as follows:

$$K_x = \frac{\frac{\%_{is}}{\%_{product}}}{\frac{Wt_{is}}{Wt_{product}}}$$

Where $\%_{is}$ and $\%_{product}$ refer to the peak area percentage of the internal standard and the product, respectively, in the chromatogram. Wt_{is} and $Wt_{product}$ refer to the know masses of internal standard and product which were added to the reference solutions.

A calculated mass of product ($Wt_{product}$) in subsequent reactions can then be deduced using the formula:

$$Wt_{product} = K_x \times Wt_{is} \times \frac{\%_{product}}{\%_{is}}$$

Spotfire was used for data visualisation. The raw data for all three plates is detailed below:

Plate 1: 1-chloro-4-nitrobenzene

Ligand	Base	%product/%IS	Calculated mass of product	%Yield
RuPhos	K_2CO_3	2.487	7.41	67.53
RuPhos	Cs_2CO_3	2.872	8.56	77.98
RuPhos	K_3PO_4	2.93	8.73	79.56
RuPhos	KO*t*Bu	1.803	5.37	48.96
QPhos	K_2CO_3	2.702	8.05	73.37
QPhos	Cs_2CO_3	2.073	6.18	56.29
QPhos	K_3PO_4	2.688	8.01	72.99
QPhos	KO*t*Bu	0.848	2.53	23.03
AmPhos	K_2CO_3	2.637	7.86	71.60
AmPhos	Cs_2CO_3	1.833	5.46	49.77
AmPhos	K_3PO_4	2.886	8.60	78.36
AmPhos	KO*t*Bu	1.518	4.52	41.22
XantPhos	K_2CO_3	2.291	6.82	62.21
XantPhos	Cs_2CO_3	1.677	5.00	45.54
XantPhos	K_3PO_4	2.765	8.24	75.08
XantPhos	KO*t*Bu	1.315	3.92	35.71
rac-BINAP	K_2CO_3	2.6	7.75	70.60
rac-BINAP	Cs_2CO_3	2.479	7.38	67.31
rac-BINAP	K_3PO_4	2.978	8.87	80.86
rac-BINAP	KO*t*Bu	0.975	2.90	26.47
CataCXium PIntB	K_2CO_3	1.633	4.86	44.34
CataCXium PIntB	Cs_2CO_3	1.018	3.03	27.64
CataCXium PIntB	K_3PO_4	1.974	5.88	53.60
CataCXium PIntB	KO*t*Bu	0.59	1.76	16.02
dppf	K_2CO_3	2.25	6.70	61.09
dppf	Cs_2CO_3	1.646	4.90	44.69
dppf	K_3PO_4	2.331	6.94	63.29
dppf	KO*t*Bu	1.264	3.77	34.32
DTBPF	K_2CO_3	2.823	8.41	76.65
DTBPF	Cs_2CO_3	2.637	7.86	71.60
DTBPF	K_3PO_4	2.908	8.66	78.96

DTBPF	KO*t*Bu	2.297	6.84	62.37
SPhos	K_2CO_3	2.977	8.87	80.83
SPhos	Cs_2CO_3	2.938	8.75	79.78
SPhos	K_3PO_4	3.011	8.97	81.76
SPhos	KO*t*Bu	1.756	5.23	47.68
P^tBu_3	K_2CO_3	2.914	8.68	79.12
P^tBu_3	Cs_2CO_3	2.204	6.57	59.84
P^tBu_3	K_3PO_4	2.665	7.94	72.36
P^tBu_3	KO*t*Bu	1.309	3.90	35.54
XPhos	K_2CO_3	2.235	6.66	60.69
XPhos	Cs_2CO_3	1.342	4.00	36.44
XPhos	K_3PO_4	2.545	7.58	69.10
XPhos	KO*t*Bu	0.927	2.76	25.17

Plate 2: 1-chloro-4-methoxybenzene

Ligand	Base	%PAR Product	%PAR IS	calc. Wt Product	%yield
RuPhos	K_2CO_3	21.757	6.504	11.22	98.26
RuPhos	Cs_2CO_3	4.906	7.138	2.31	20.19
RuPhos	K_3PO_4	21.094	6.46	10.95	95.91
RuPhos	KO*t*Bu	14.096	5.55	8.52	74.60
QPhos	K_2CO_3	16.095	6.612	8.17	71.50
QPhos	Cs_2CO_3	15.585	6.597	7.92	69.39
QPhos	K_3PO_4	16.72	6.808	8.24	72.14
QPhos	KO*t*Bu	15.282	6.94	7.39	64.68
AmPhos	K_2CO_3	20.771	6.734	10.35	90.60
AmPhos	Cs_2CO_3	24.214	7.312	11.11	97.27
AmPhos	K_3PO_4	15.431	7.155	7.23	63.35
AmPhos	KO*t*Bu	4.716	7.468	2.12	18.55
XantPhos	K_2CO_3	0.794	6.005	0.44	3.88
XantPhos	Cs_2CO_3	0.786	6.098	0.43	3.79
XantPhos	K_3PO_4	1.2	6.034	0.67	5.84
XantPhos	KO*t*Bu	0.954	5.799	0.55	4.83
rac-BINAP	K_2CO_3	0.375	5.494	0.23	2.00
rac-BINAP	Cs_2CO_3	0.39	5.388	0.24	2.13
rac-BINAP	K_3PO_4	0.639	5.708	0.38	3.29
rac-BINAP	KO*t*Bu	0	6.58	0	0
CataCXium PIntB	K_2CO_3	9.884	7.584	4.37	38.28

CataCXium PIntB	Cs_2CO_3	10.611	6.07	5.86	51.35
CataCXium PIntB	K_3PO_4	11.047	5.538	6.69	58.59
CataCXium PIntB	KO*t*Bu	1.034	5.833	0.59	5.21
dppf	K_2CO_3	0.077	5.64	0.05	0.40
dppf	Cs_2CO_3	0	5.23	0	0
dppf	K_3PO_4	0.295	5.935	0.17	1.46
dppf	KO*t*Bu	NA	NA	NA	NA*
DTBPF	K_2CO_3	9.425	5.753	5.50	48.12
DTBPF	Cs_2CO_3	11.061	5.758	6.44	56.43
DTBPF	K_3PO_4	11.812	5.746	6.90	60.38
DTBPF	KO*t*Bu	6.715	5.78	3.90	34.12
SPhos	K_2CO_3	18.999	6.717	9.49	83.08
SPhos	Cs_2CO_3	21.708	6.832	10.66	93.33
SPhos	K_3PO_4	18.767	6.035	10.43	91.34
SPhos	KO*t*Bu	16.492	6.757	8.19	71.69
P^tBu_3	K_2CO_3	7.896	6.662	3.98	34.81
P^tBu_3	Cs_2CO_3	10.307	6.51	5.31	46.51
P^tBu_3	K_3PO_4	12.306	7.3	5.65	49.52
P^tBu_3	KO*t*Bu	1.206	5.898	0.69	6.01
XPhos	K_2CO_3	16.856	6.413	8.82	77.21
XPhos	Cs_2CO_3	17.744	6.401	9.30	81.42
XPhos	K_3PO_4	21.295	6.923	10.32	90.35
XPhos	KO*t*Bu	16.182	6.301	8.61	75.44

*Dosing error – reaction invalid.

Plate 3: 2-chloro-1,3-dimethylbenzene

Ligand	Base	%PAR Product	%PAR IS	calc. Wt Product	%yield
RuPhos	K_2CO_3	12.981	5.603	8.11	84.38
RuPhos	Cs_2CO_3	16.832	6.407	9.20	95.69
RuPhos	K_3PO_4	16.329	5.79	9.88	102.72
RuPhos	KO*t*Bu	14.969	6.098	8.60	89.41
QPhos	K_2CO_3	7.061	5.656	4.37	45.47
QPhos	Cs_2CO_3	4.887	5.62	3.05	31.67
QPhos	K_3PO_4	7.405	5.508	4.71	48.97
QPhos	KO*t*Bu	5.077	5.243	3.39	35.27
AmPhos	K_2CO_3	13.433	5.033	9.35	97.21

AmPhos	Cs_2CO_3	17.397	6.576	9.27	96.36
AmPhos	K_3PO_4	12.564	4.84	9.09	94.55
AmPhos	KO*t*Bu	7.04	4.686	5.26	54.72
XantPhos	K_2CO_3	1.959	5.934	1.16	12.02
XantPhos	Cs_2CO_3	1.899	5.66	1.18	12.22
XantPhos	K_3PO_4	2.373	5.869	1.41	14.73
XantPhos	KO*t*Bu	2.034	5.042	1.41	14.69
rac-BINAP	K_2CO_3	2.495	5.454	1.60	16.66
rac-BINAP	Cs_2CO_3	3.327	5.473	2.13	22.14
rac-BINAP	K_3PO_4	1.415	5.298	0.94	9.73
rac-BINAP	KO*t*Bu	0	5.884	0	0
CataCXium PIntB	K_2CO_3	2.401	5.423	1.55	16.13
CataCXium PIntB	Cs_2CO_3	0.921	5.54	0.58	6.06
CataCXium PIntB	K_3PO_4	2.557	5.626	1.59	16.55
CataCXium PIntB	KO*t*Bu	5.578	5.413	3.61	37.53
dppf	K_2CO_3	0.093	4.651	0.07	0.73
dppf	Cs_2CO_3	0.145	5.729	0.09	0.92
dppf	K_3PO_4	0.122	6.012	0.07	0.74
dppf	KO*t*Bu	0.056	6.239	0.03	0.33
DTBPF	K_2CO_3	8.652	5.185	5.84	60.78
DTBPF	Cs_2CO_3	8.971	5.211	6.03	62.70
DTBPF	K_3PO_4	9.03	5.121	6.18	64.22
DTBPF	KO*t*Bu	12.092	5.363	7.90	82.12
SPhos	K_2CO_3	16.026	5.848	9.60	99.81
SPhos	Cs_2CO_3	15.277	5.877	9.10	94.68
SPhos	K_3PO_4	17.179	6.227	9.66	100.48
SPhos	KO*t*Bu	16.038	5.921	9.49	98.66
P^tBu_3	K_2CO_3	7.796	5.067	5.39	56.04
P^tBu_3	Cs_2CO_3	10.452	6.133	5.97	62.07
P^tBu_3	K_3PO_4	8.431	6.276	4.70	48.93
P^tBu_3	KO*t*Bu	3.64	4.983	2.56	26.61
XPhos	K_2CO_3	11.897	5.944	7.01	72.90
XPhos	Cs_2CO_3	10.556	5.85	6.32	65.72
XPhos	K_3PO_4	11.733	5.923	6.94	72.15
XPhos	KO*t*Bu	12.12	5.728	7.41	77.07

4. Additive Screen Protocol

Method details

All analysis was conducted using an Agilent 7890 and an FID as the detector. The exact method details are as follows:

Column: Agilent HP-5; 30m × 0.32 mm × 0.25 μm.
Carrier gas: Helium.
Carrier gas flow: 1.2 mL/min.
Injector temperature: 300 °C.
Injection volume: 0.5 μL.
Detector temperature: 320 °C.
Run time: 19.5 min.
Oven temperature program:

Ramp Rate (°C/min)	Final temperature (°C)	Hold time (min)
-	80	3
5	90	0
10	130	0
20	280	3

Calibration

The calibration curve below was used to determine product yield based on FID response relative to internal standard (1,3,5-trimethoxybenzene).

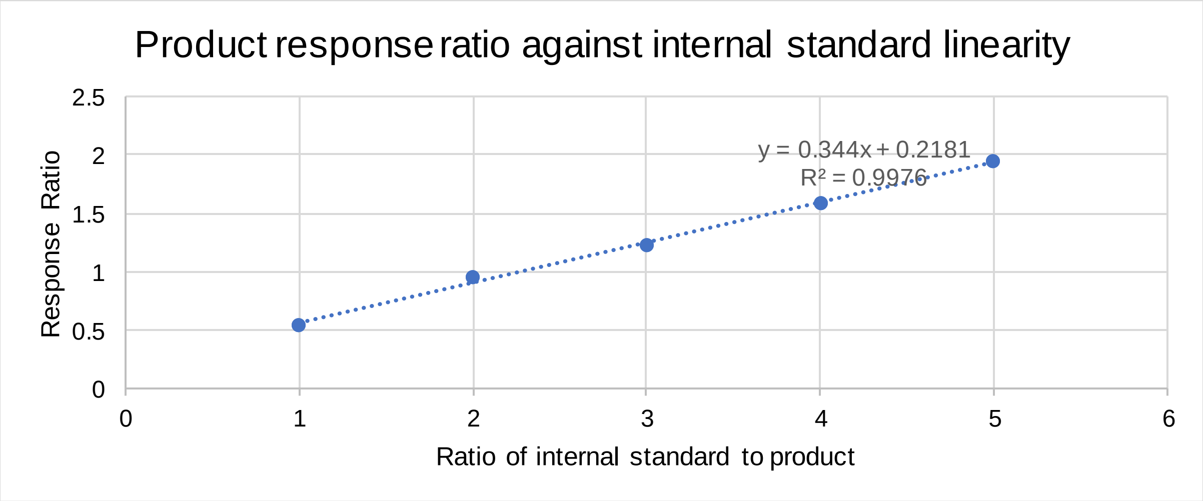

The solution yield of product in each reaction could then be calculated using the following equation:

$$\%yield = \left(\frac{0.344}{PAR_{\frac{internal\ standard}{product}} - 0.2181}\right) \times 100$$

A simplified batch calibration method was used for the additives as detailed by Collins and Glorius.[16] After analysing each additive separately to find their respective retention times, two stock solutions were made up, each containing 8 additives (0.1 mmol of each additive),

and 1,3,5-trimethoxybenzene (0.2 mmol) in 20 mL toluene. The retention times were as follows:

Stock Solution A		Stock Solution B	
Additive	Retention time	Additive	Retention time
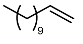 **A**	9.68	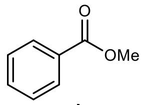 **I**	8.13
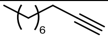 **B**	6.71	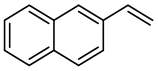 **J**	12.38
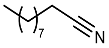 **C**	10.90	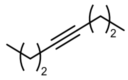 **K**	3.91
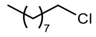 **D**	9.28	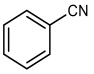 **L**	6.11
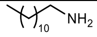 **E**	11.55	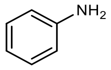 **M**	5.95
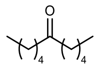 **F**	10.74	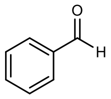 **N**	5.68
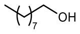 **G**	9.36	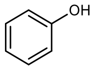 **O**	5.84
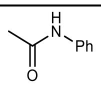 **H**	11.87	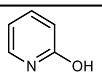 **P**	8.85
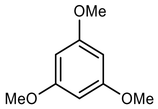	12.14	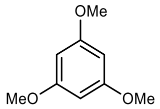	12.14

The raw data for the stock solution chromatograms is detailed below:

Run	Reagent name	Reagent area uV*sec	1,3,5-trimethoxybenzene (IS)area uV*sec	Peak area ratio of reagent to internal standard
Stock Solution A – Run 1	1- dodecene	200.781977	105.81705	1.897444422
	1-decyne	213.296013		2.015705474
	decanenitrile	174.404597		1.64817099
	1-chlorononane	157.685203		1.490168158
	dodecylamine	167.882922		1.586539383
	6-undecanone	168.471620		1.59210274
	1-nonanol	155.678391		1.471203238
	acetanilide	92.571679		0.874827605
Stock Solution A – Run 2	1- dodecene	201.892249	106.56336	1.894574748
	1-decyne	214.310949		2.011112929
	decanenitrile	175.455784		1.646492619
	1-chlorononane	158.398430		1.486424898
	dodecylamine	169.067598		1.586545315
	6-undecanone	169.437986		1.590021069
	1-nonanol	157.161857		1.474820787
	acetanilide	96.083880		0.901659641
Stock Solution B – Run 1	methyl benzoate	83.085549	111.82861	0.742972239
	2-vinylnapthanlene	132.928484		1.188680517
	4-octyne	80.146579		0.716691217
	benzonitrile	83.881968		0.750094021
	aniline	61.913092		0.553642711
	benzaldehyde	68.285419		0.610625689
	phenol	69.978194		0.625762916
	2-hydroxylpyridine	50.184619		0.448763704
Stock Solution B – Run 2	methyl benzoate	82.495838	110.79171	0.744602972
	2-vinylnapthanlene	131.661741		1.188371753
	4-octyne	79.790585		0.720185504
	benzonitrile	83.559106		0.754199971
	aniline	61.530058		0.555366975
	benzaldehyde	68.026452		0.614003076
	phenol	69.647420		0.628633846
	2-hydroxylpyridine	50.152551		0.452674213

The mean response ratios for 2:1 internal standard:additive:

Reagent	Mean peak area ratio of additive to internal standard.
A	1.8960
B	2.0134
C	1.6473
D	1.4883
E	1.5865
F	1.5911
G	1.4730
H	0.8882
I	0.7438
J	1.1885
K	0.7184
L	0.7521
M	0.5545
N	0.6123
O	0.6272
P	0.4507

Given that the additve:internal standard ratio in the screen was 1:1, the %additive remaining in each reaction could then be calculated as follows:

$$\%_{Additive\ Remaining} = \left(\frac{Peak\ area(additive)}{2 \times Peak\ area\ (internal\ standard) \times PAR_{calibration}}\right) \times 100$$

Procedure for screening

In the glovebox, 16 vials equipped with stirrer bars were charged with tribasic potassium phosphate (64.0 mg, 0.30 mmol, 3.0 equiv), SPhos Pd G3 (7.80 mg, 0.010 mmol, 0.10 equiv), and potassium methyl trifluoroborate (13.0 mg, 0.11 mmol, 1.1 equiv). To each vial was then added a 1M stock solution containing 4-chlorobiphenyl and 1,3,5-trimethoxybenzene (100 µL, 0.10 mmol, 1.0 equiv) in toluene. Each vial was then charged with a 1M stock solution of additives A-P (100 µL, 1.0 mmol, 1.0 equiv) in toluene. The vials were then charged with 250 µL toluene and 50 µL water before being sealed with a teflon coated crimp cap and removed from the glovebox. The 16 reaction mixtures were then stirred at 80 °C for 24 hours before being cooled to room temperature, and diluted with 550 µL toluene. An 80 µL aliquot of each reaction mixture was taken and diluted with a further 1.5 mL of toluene. These samples were analysed by GC-FID.

Results

The raw data for the peak area of product, internal standard, and additive from each of the 16 reactions is given below:

	Peak Area		
**Reaction**	**internal standard**	**product**	**additive**
**A**	64.94	120.63	64.18
**B**	72.06	84.10	0
**C**	69.26	130.72	172.82
**D**	69.71	132.34	159.54
**E**	65.52	121.43	156.03
**F**	64.24	120.81	168.15
**G**	70.60	121.93	151.12
**H**	72.58	136.08	127.96
**I**	64.16	116.66	79.05
**J**	68.51	132.85	125.87
**K**	67.30	82.57	5.56
**L**	67.61	126.53	80.09
**M**	66.82	117.72	61.96
**N**	66.69	126.44	61.18
**O**	66.03	126.96	61.65
**P**	63.26	0	26.94

Inputting the obtained date into the formulas above gave %yields of product and remaining additive as detailed below:

Reaction	%yield (product)	%additive remaining
A	95.3	26.1
B	55.2	0
C	97.0	75.7
D	97.7	76.9
E	95.1	75.1
F	96.6	82.2
G	87.7	72.7
H	96.3	99.2
I	93.0	82.8
J	100.1	77.3
K	58.7	5.8
L	96.1	78.7
M	89.7	83.6
N	97.5	74.9
O	99.1	74.4
P	0	47.2

5. References

(1) Velasco, R.; Feberero, C.; Sanz, R. α-Lithiated Aryl Benzyl Ethers: Inhibition of [1,2]-Wittig Rearrangement and Application to the Synthesis of Benzo[*b*]furan Derivatives. *Org. Lett.* **2015**, *17*, 4416-4419.

(2) Mori, A.; Mizusaki, T.; Ikawa, T.; Maegawa, T.; Monguchi, Y.; Sajiki, H. Mechanistic Study of a Pd/C-Catalyzed Reduction of Aryl Sulfonates Using the Mg–MeOH–NH_4OAc System. *Chem. Eur. J.* **2007**, *13*, 1432-1441.

(3) Glasspoole, B. W.; Ghozati, K.; Moir, J. W.; Crudden, C. M. Suzuki–Miyaura cross-couplings of secondary allylic boronic esters. *Chem. Commun.* **2012**, *48*, 1230-1232.

(4) Clegg, W.; Johann, T. R. F.; Marder, T. B.; Norman, N. C.; Orpen, A. G.; Peakman, T. M.; Quayle, M. J.; Rice, C. R.; Scott, A. J. Platinum-catalysed 1,4-diboration of 1,3-dienes. *J. Chem. Soc., Dalton Trans.* **1998**, 1431-1438.

(5) Lennox, A. J. J.; Lloyd-Jones, G. C. Preparation of Organotrifluoroborate Salts: Precipitation-Driven Equilibrium under Non-Etching Conditions. *Angew. Chem. Int. Ed.* **2012**, *51*, 9385-9388.

(6) Penning, T. D.; Talley, J. J.; Bertenshaw, S. R.; Carter, J. S.; Collins, P. W.; Docter, S.; Graneto, M. J.; Lee, L. F.; Malecha, J. W.; Miyashiro, J. M.; Rogers, R. S.; Rogier, D. J.; Yu, S. S.; Anderson, G. D.; Burton, E. G.; Cogburn, J. N.; Gregory, S. A.; Koboldt, C. M.; Perkins, W. E.; Seibert, K.; Veenhuizen, A. W.; Zhang, Y. Y.; Isakson, P. C. Synthesis and Biological Evaluation of the 1,5-Diarylpyrazole Class of Cyclooxygenase-2 Inhibitors: Identification of 4-[5-(4-Methylphenyl)-3-(trifluoromethyl)-1*H*-pyrazol-1-yl]benzenesulfonamide (SC-58635, Celecoxib). *J. Med. Chem.* **1997**, *40*, 1347-1365.

(7) Chatterjee, N.; Bhatt, D.; Goswami, A. A novel transition metal free [bis-(trifluoroacetoxy)iodo]benzene (PIFA) mediated oxidative *ipso* nitration of organoboronic acids. *Org. Biomol. Chem.* **2015**, *13*, 4828-4832.

(8) Dilauro, G.; Azzollini, C. S.; Vitale, P.; Salomone, A.; Perna, F. M.; Capriati, V. Scalable Negishi Coupling between Organozinc Compounds and (Hetero)Aryl Bromides under Aerobic Conditions when using Bulk Water or Deep Eutectic Solvents with no Additional Ligands. *Angew. Chem. Int. Ed.* **2021**, *60*, 10632-10636.

(9) Lin, R.; Chen, F.; Jiao, N. Metal-Free, NHPI Catalyzed Oxidative Cleavage of C–C Double Bond Using Molecular Oxygen as Oxidant. *Org. Lett.* **2012**, *14*, 4158-4161.

(10) Shu, Z.; Ye, Y.; Deng, Y.; Zhang, Y.; Wang, J. Palladium(II)-Catalyzed Direct Conversion of Methyl Arenes into Aromatic Nitriles. *Angew. Chem. Int. Ed.* **2013**, *52*, 10573-10576.

(11) Majek, M.; Jacobi von Wangelin, A. Metal-Free Carbonylations by Photoredox Catalysis. *Angew. Chem. Int. Ed.* **2015**, *54*, 2270-2274.

(12) Kariofillis, S. K.; Shields, B. J.; Tekle-Smith, M. A.; Zacuto, M. J.; Doyle, A. G. Nickel/Photoredox-Catalyzed Methylation of (Hetero)aryl Chlorides Using Trimethyl Orthoformate as a Methyl Radical Source. *J. Am. Chem. Soc.* **2020**, *142*, 7683-7689.

(13) Haydl, A. M.; Hartwig, J. F. Palladium-Catalyzed Methylation of Aryl, Heteroaryl, and Vinyl Boronate Esters. *Org. Lett.* **2019**, *21*, 1337-1341.

(14) Diebold, C.; Becht, J.-M.; Lu, J.; Toy, P. H.; Le Drian, C. An Efficient and Reusable Palladium Catalyst Supported on a Rasta Resin for Suzuki–Miyaura Cross-Couplings. *Eur. J. Org. Chem.* **2012**, *2012*, 893-896.

(15) Jiang, H.; Yang, J.; Tang, X.; Li, J.; Wu, W. Cu-Catalyzed Three-Component Cascade Annulation Reaction: An Entry to Functionalized Pyridines. *J. Org. Chem.* **2015**, *80*, 8763-8771.

(16) Collins, K. D.; Glorius, F. A robustness screen for the rapid assessment of chemical reactions. *Nat. Chem.* **2013**, *5*, 597-601.

6. NMR Spectra

^1H NMR (400 MHz, $CDCl_3$) of **13**

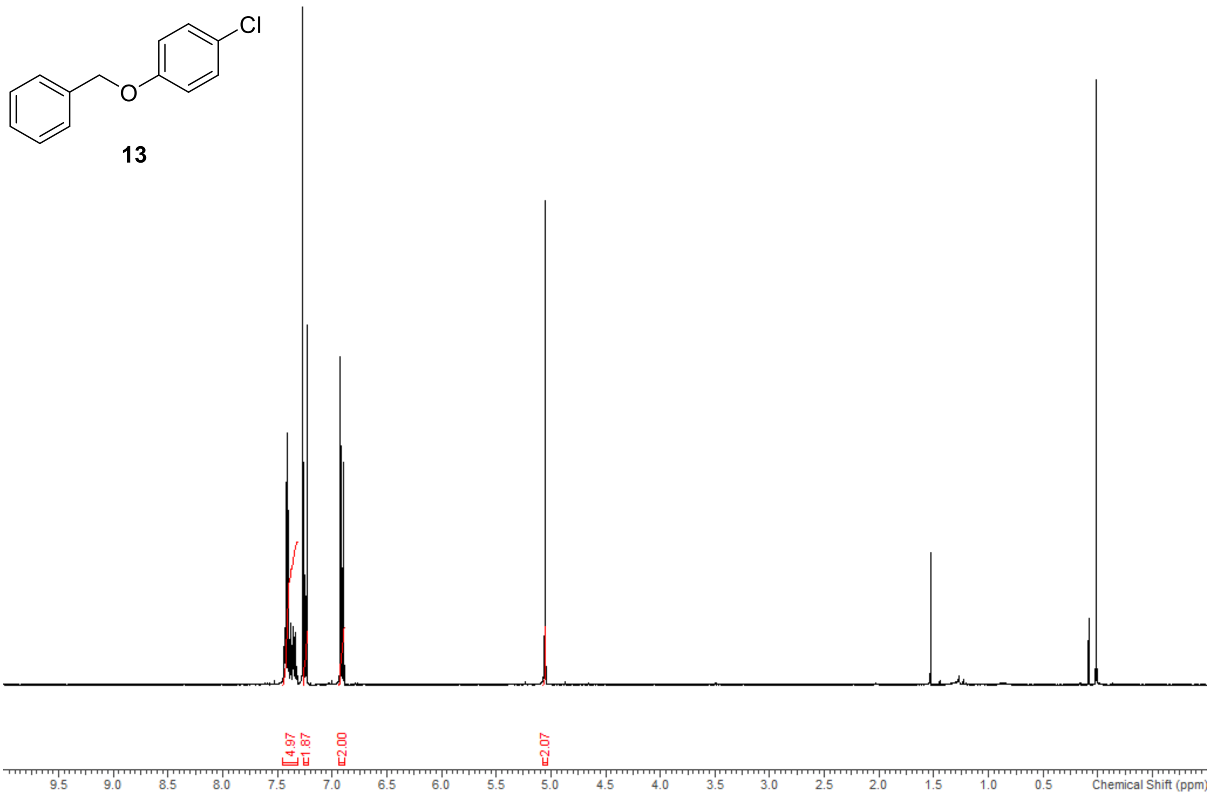

^{13}C NMR (100 MHz, $CDCl_3$) of **13**

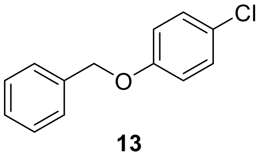

13

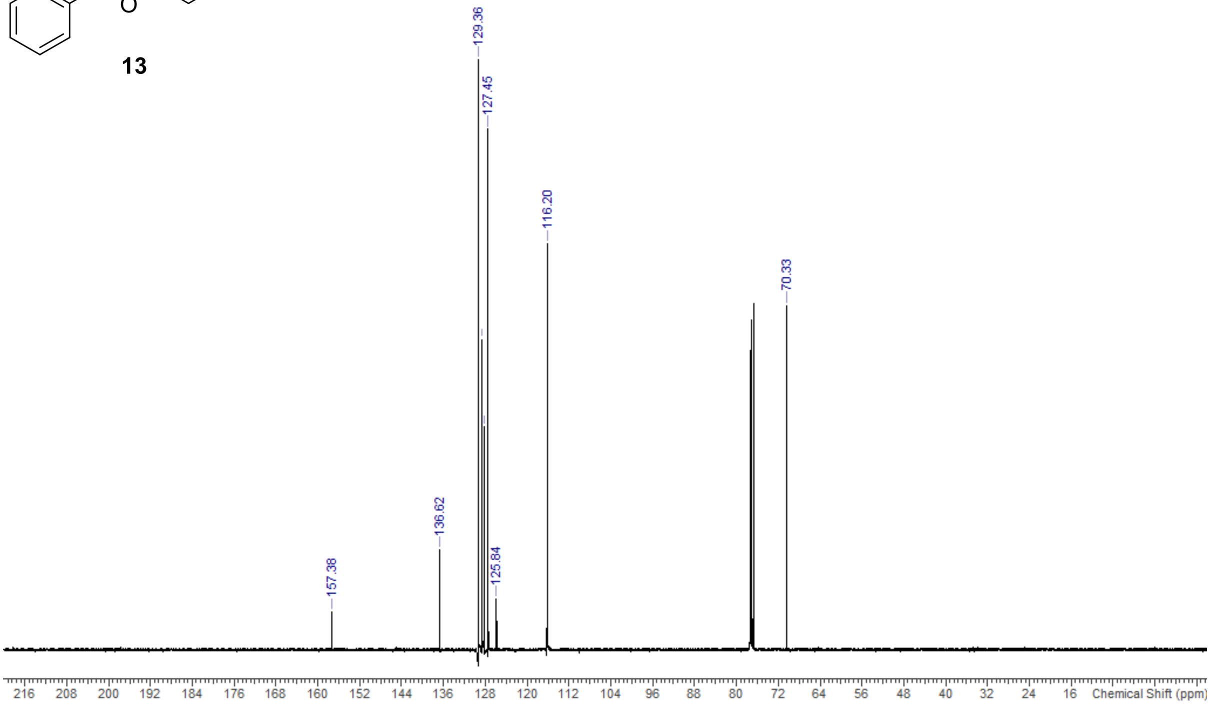

^{1}H NMR (400 MHz, $CDCl_3$) of **16**

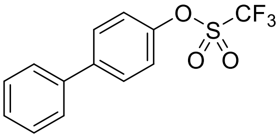

16

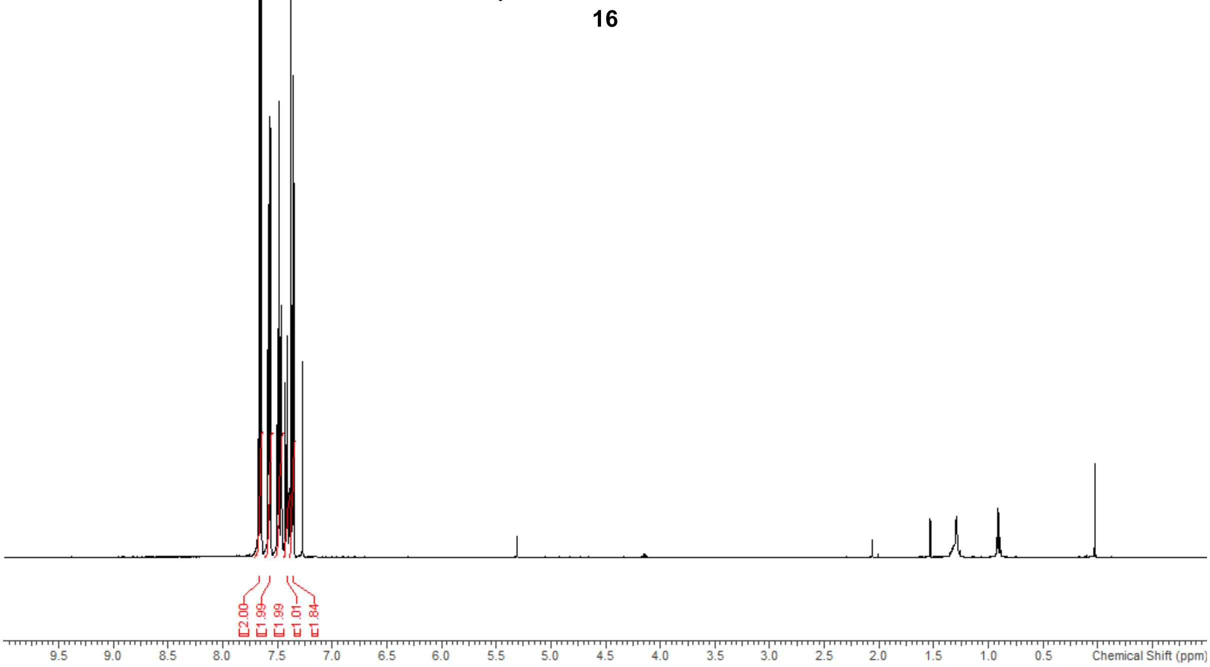

^{13}C NMR (100 MHz, $CDCl_3$) of **16**

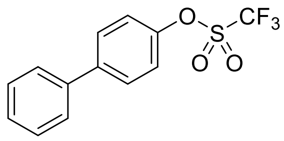

16

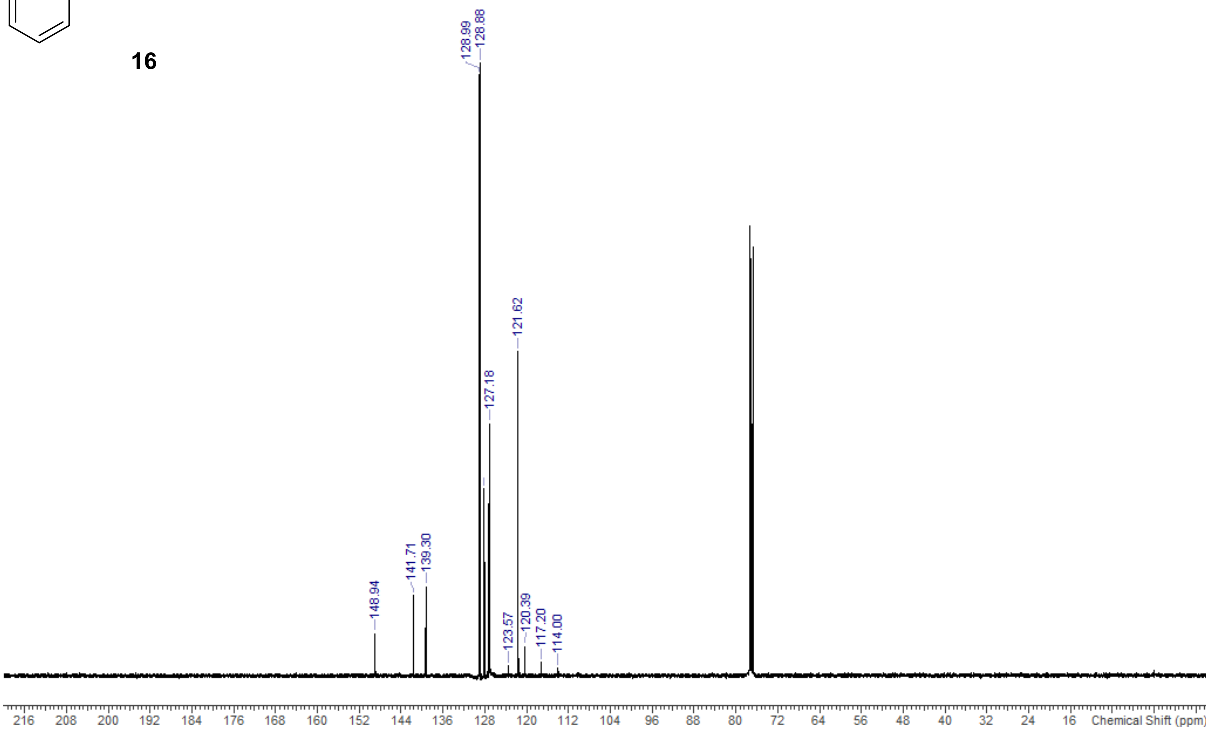

^{19}F NMR (376 MHz, $CDCl_3$) of **16**

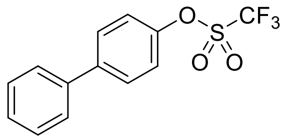

16

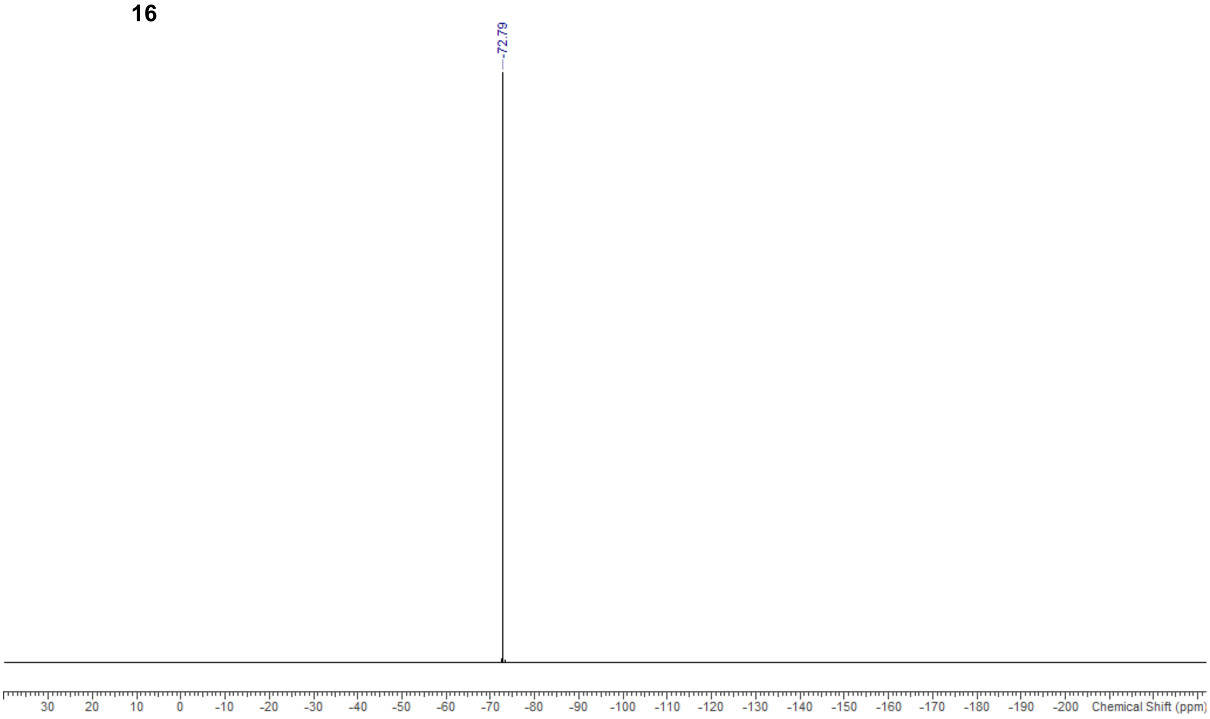

^{1}H NMR (400 MHz, $CDCl_3$) of **22**

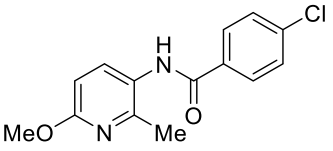

22

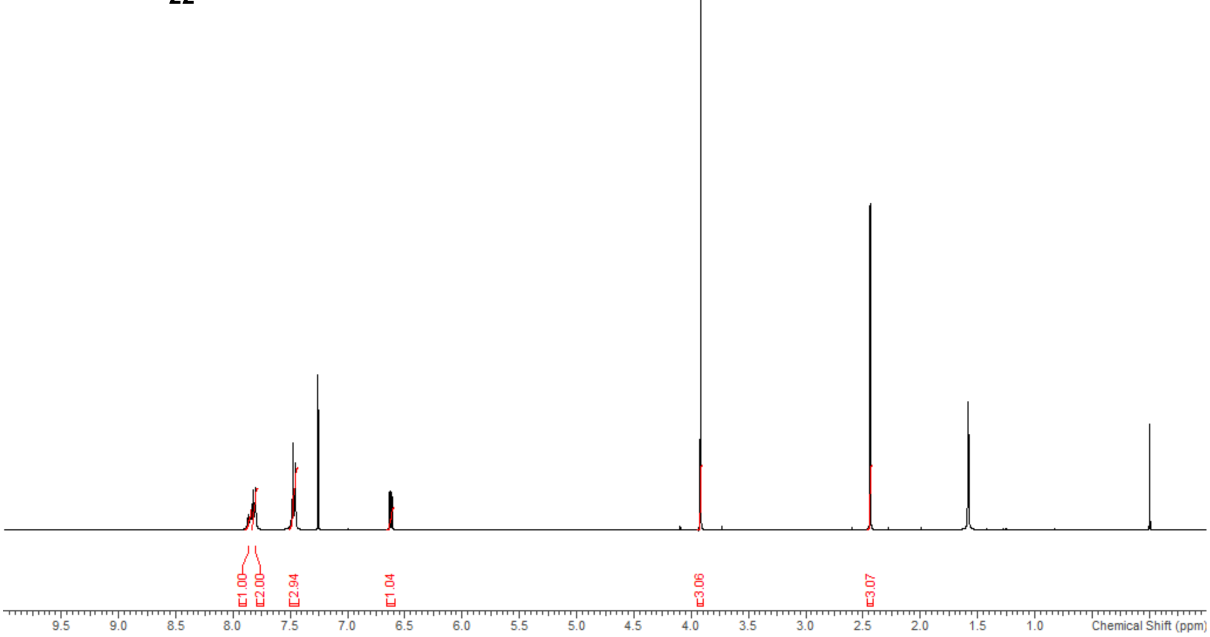

^{13}C NMR (100 MHz, $CDCl_3$) of **22**

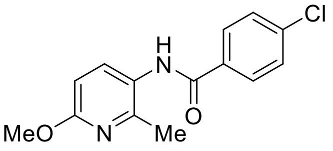

22

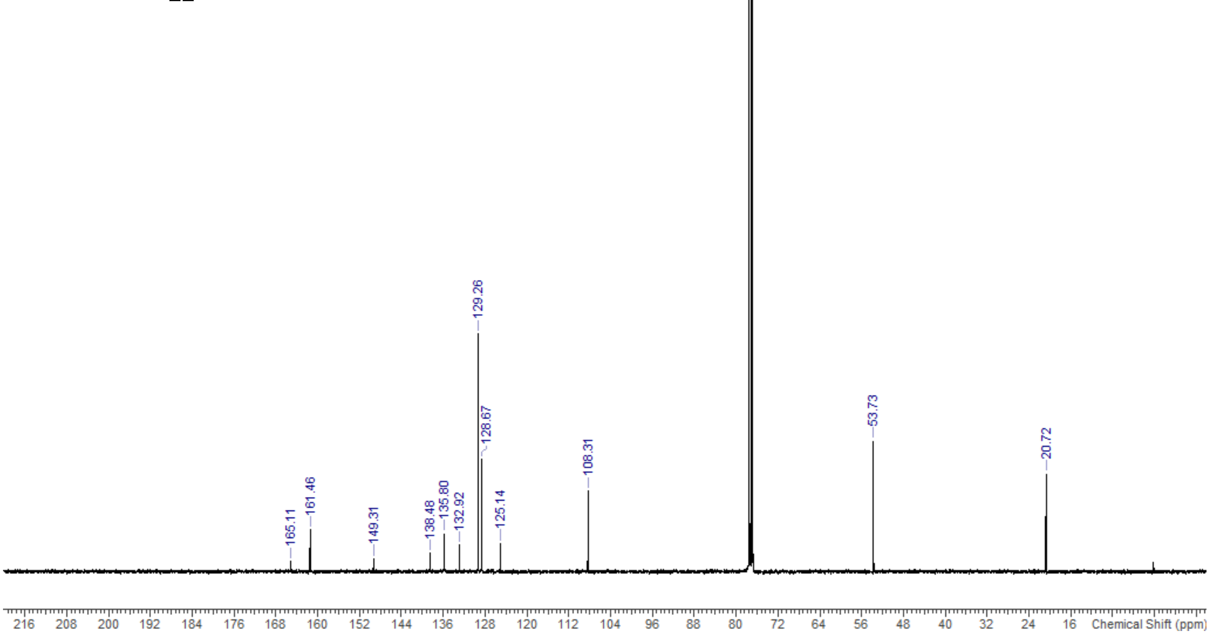

^{1}H NMR (400 MHz, $CDCl_3$) of **23** *in situ*

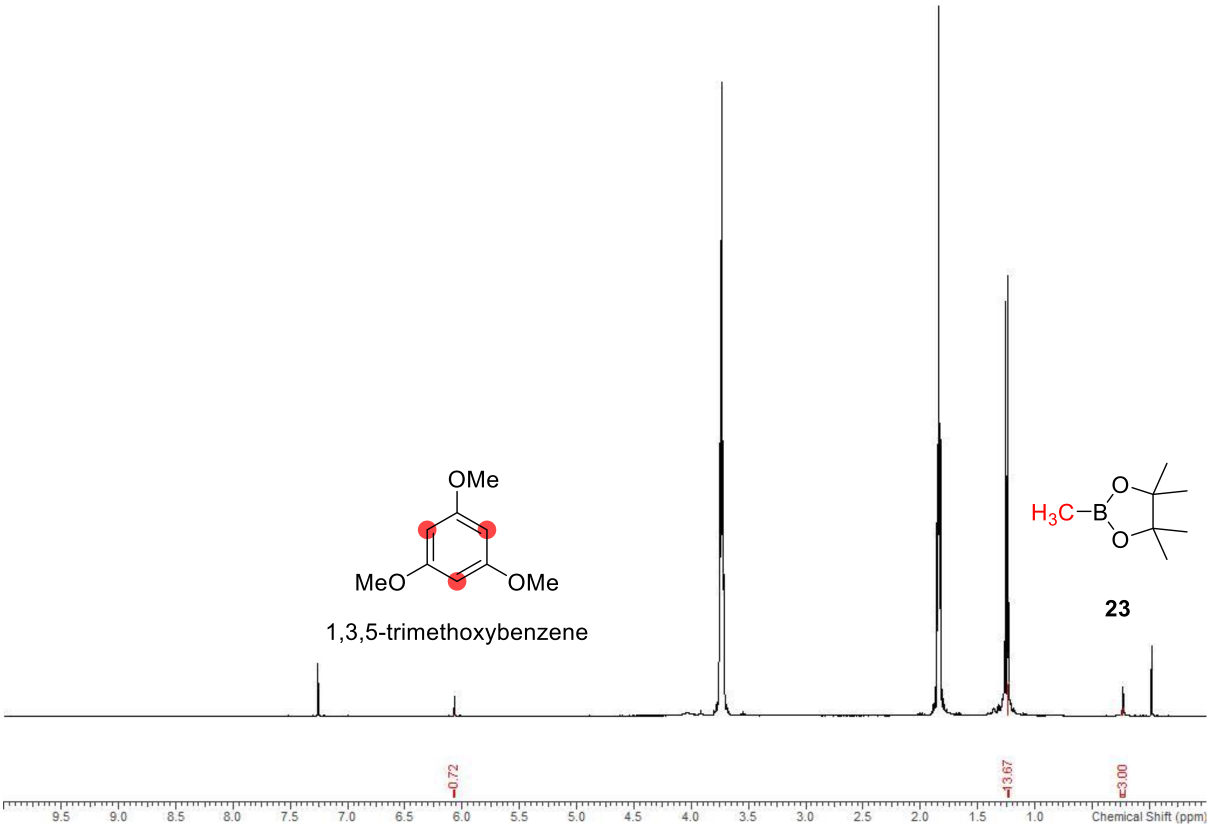

^{1}H NMR (400 MHz, $CDCl_3$) of **24**

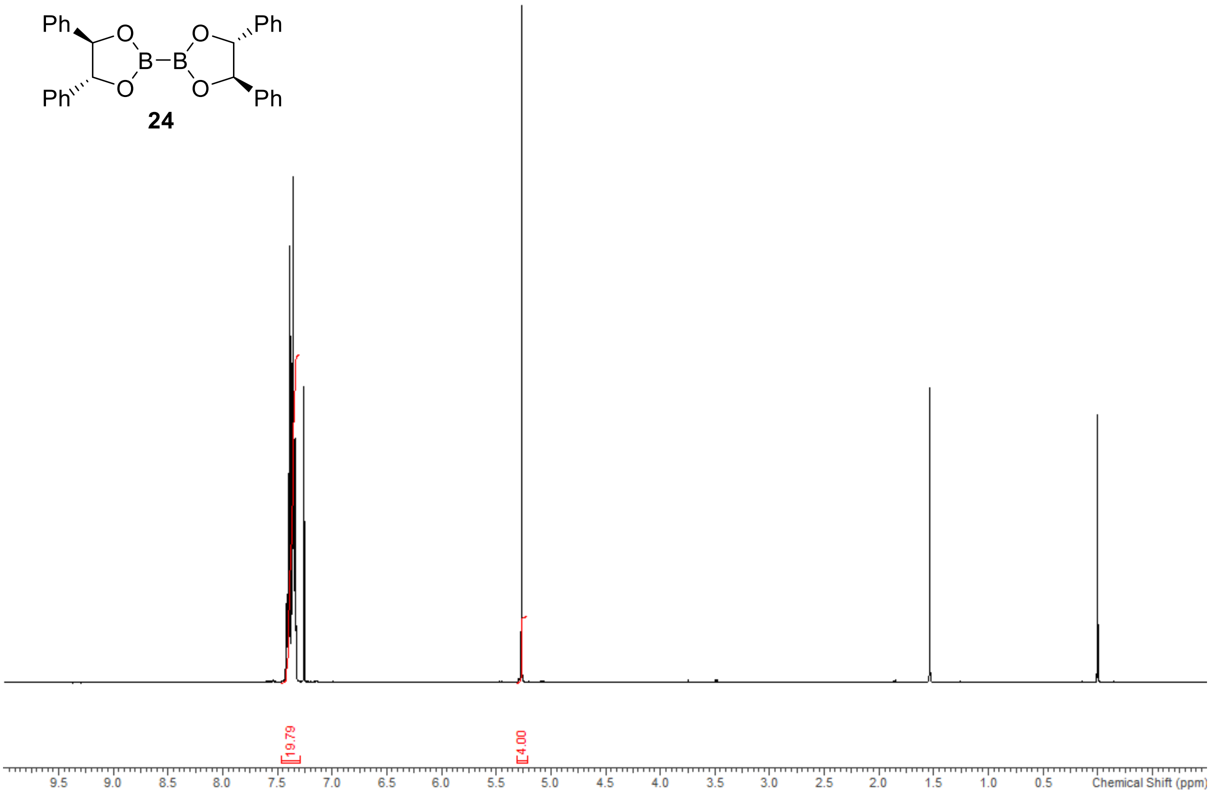

^{13}C NMR (100 MHz, $CDCl_3$) of **24**

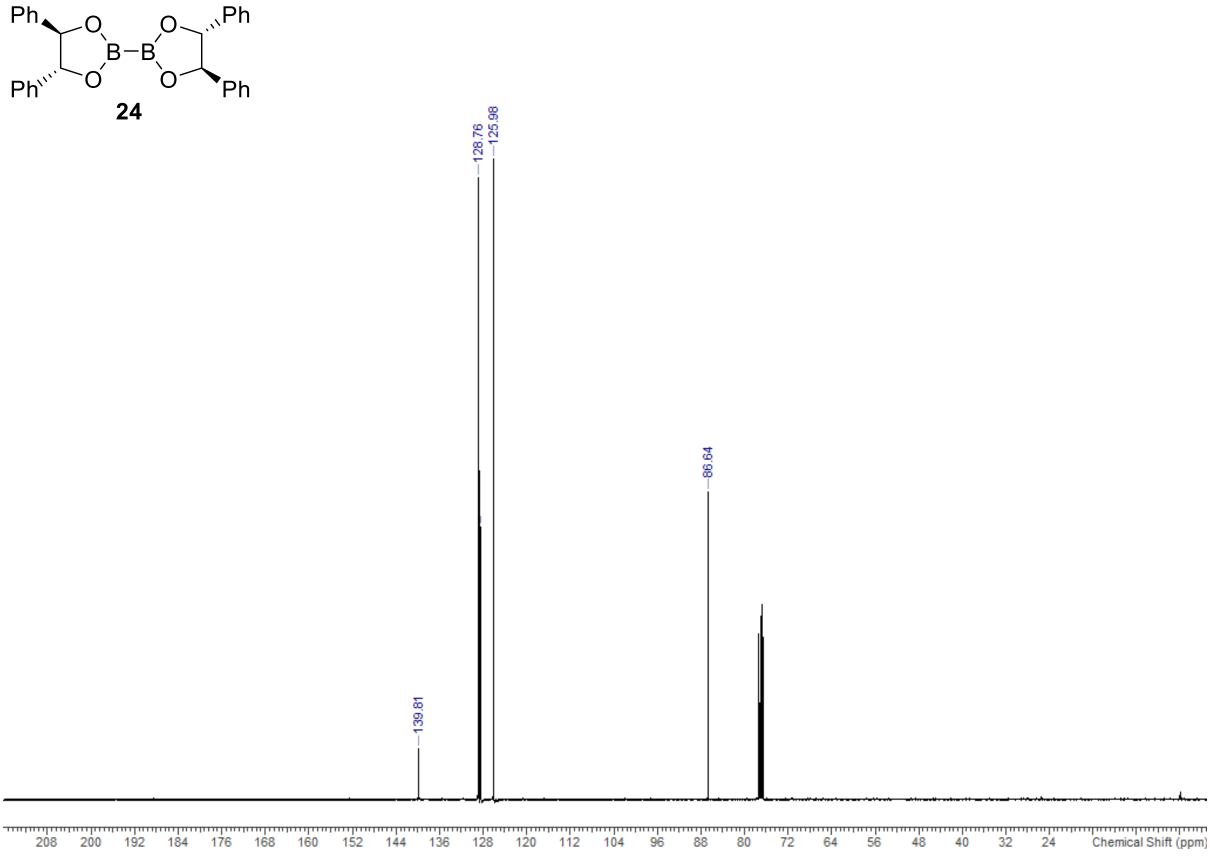

^{11}B NMR (128 MHz, $CDCl_3$) of **24**

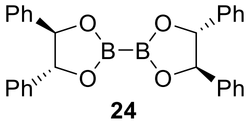

24

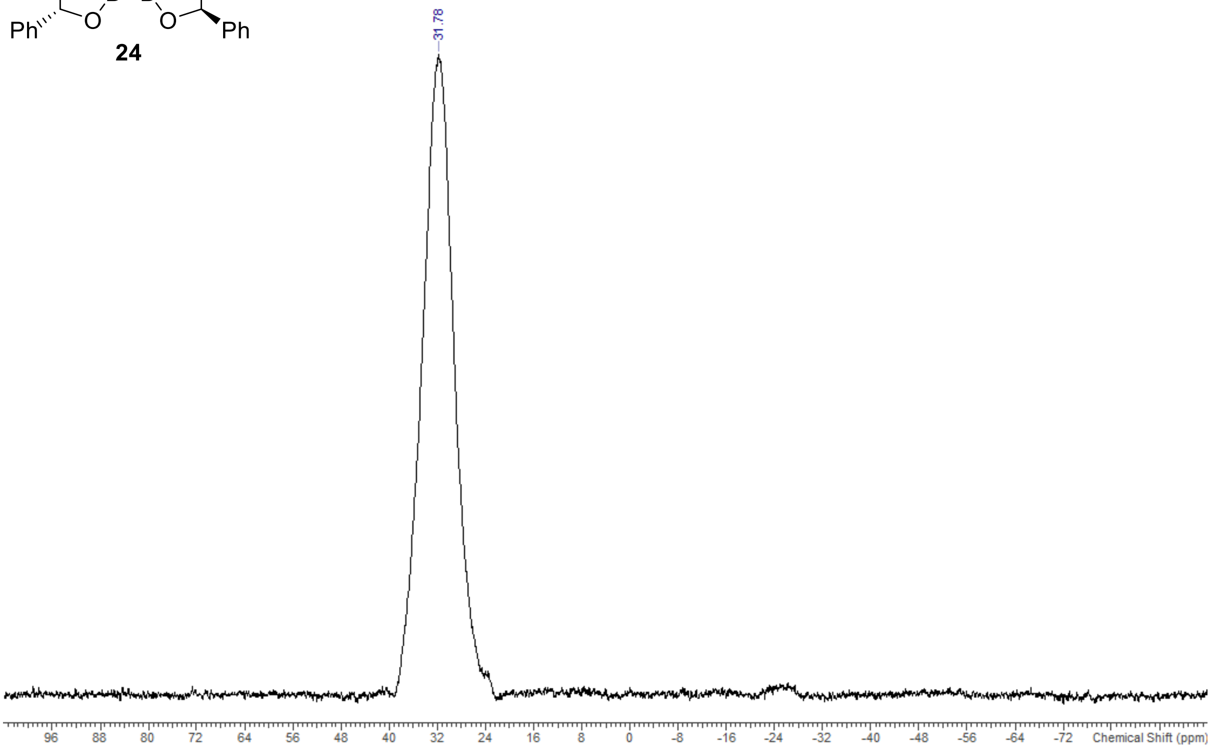

^{1}H NMR (400 MHz, $CDCl_3$) of **25a**

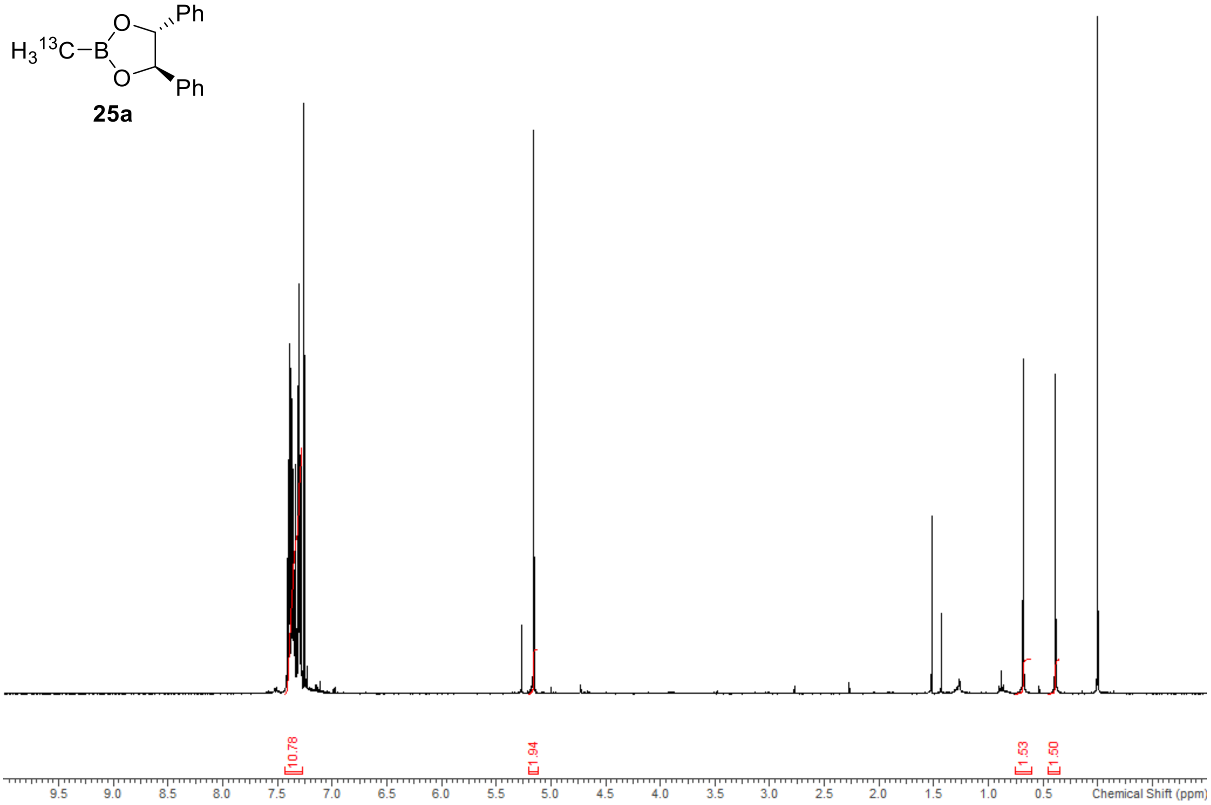

^{1}H NMR (400 MHz, $CDCl_3$) of **25**

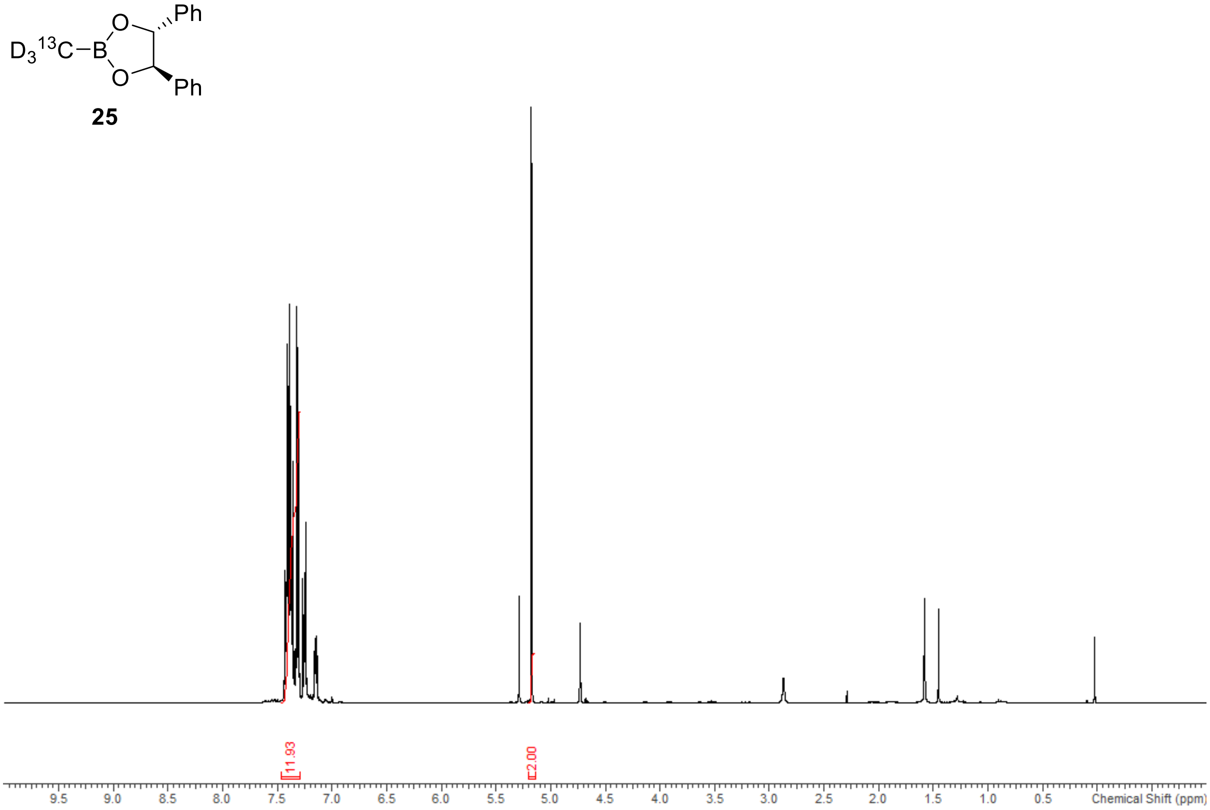

^{11}B NMR (128 MHz, $CDCl_3$) of **25**

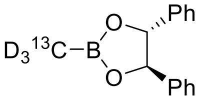

25

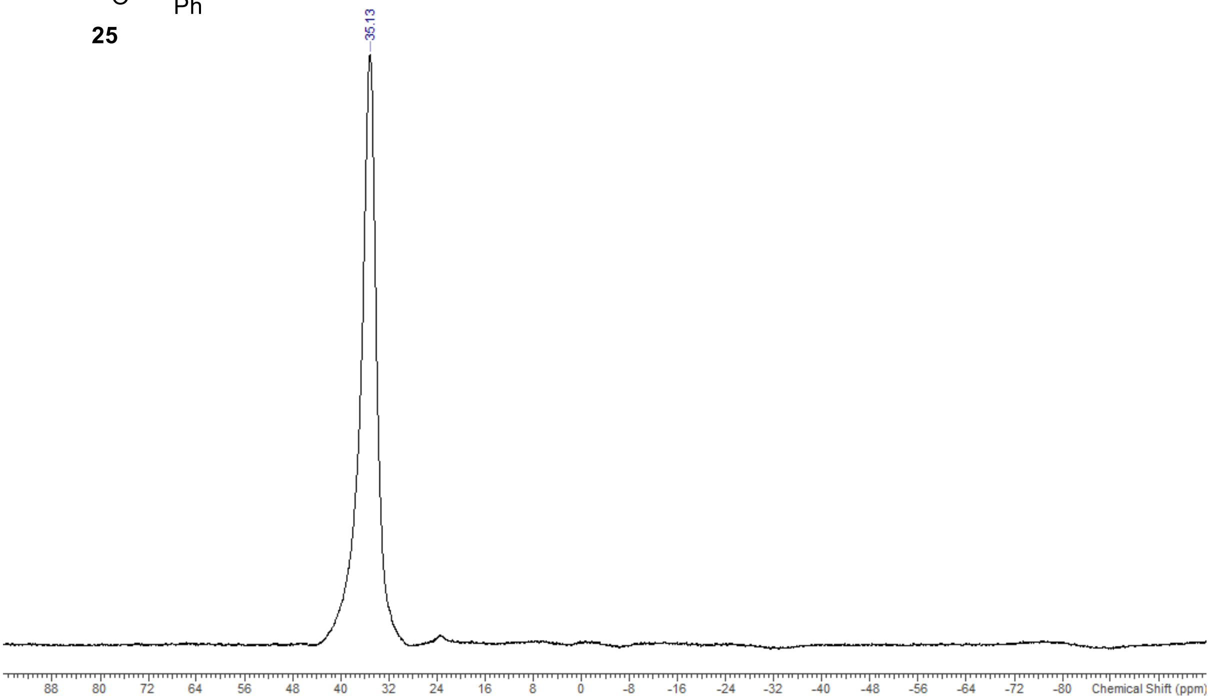

^{1}H NMR (400 MHz, DMSO-d_6) of **26a**

$H_3{}^{13}C-BF_3K$

26a

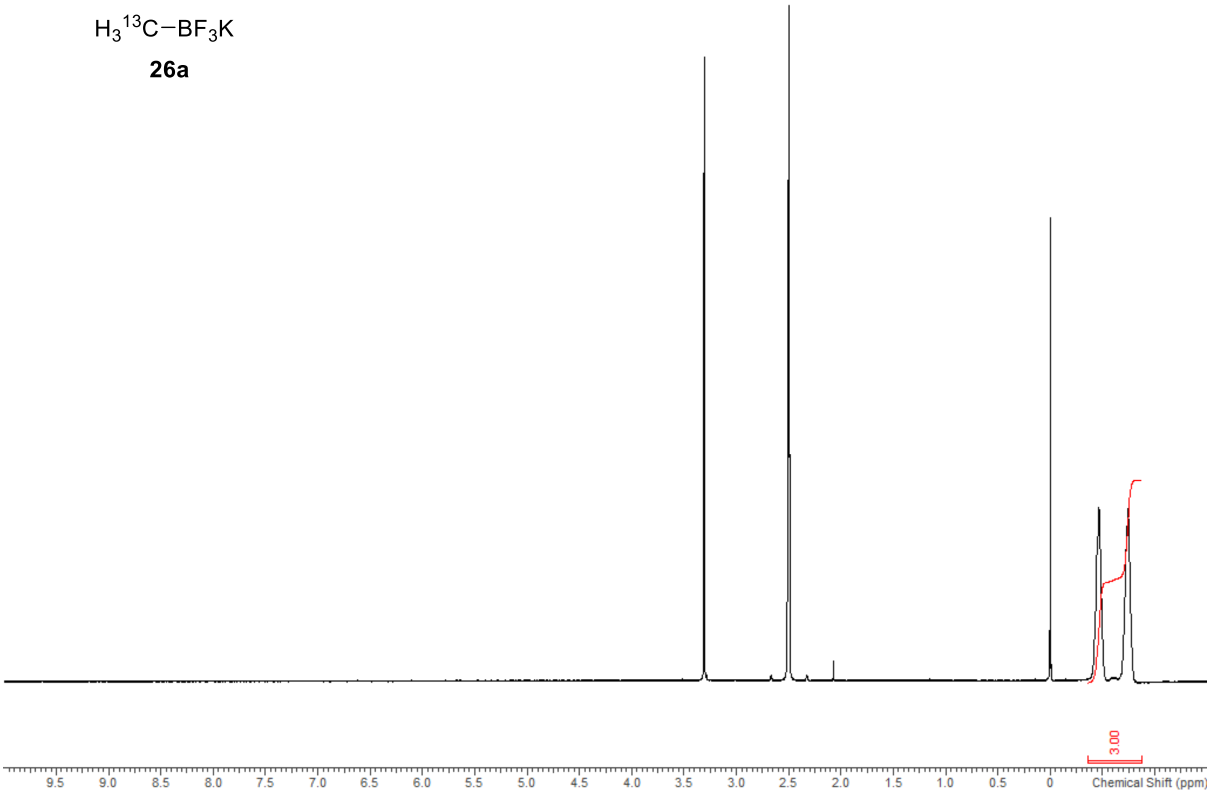

^{13}C NMR (100 MHz, DMSO-d_6) of **26a**

$H_3{}^{13}C-BF_3K$

26a

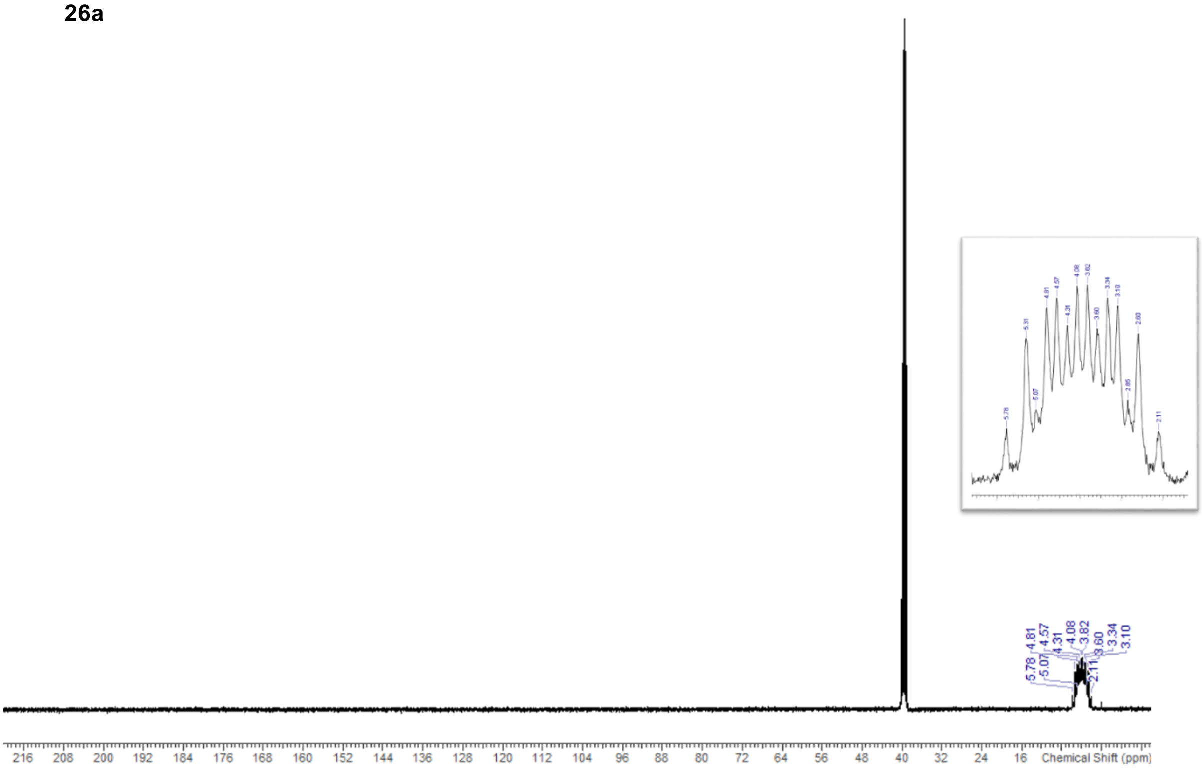

^{11}B NMR (128 MHz, DMSO-d_6) of **26a**

$H_3{}^{13}C-BF_3K$

26a

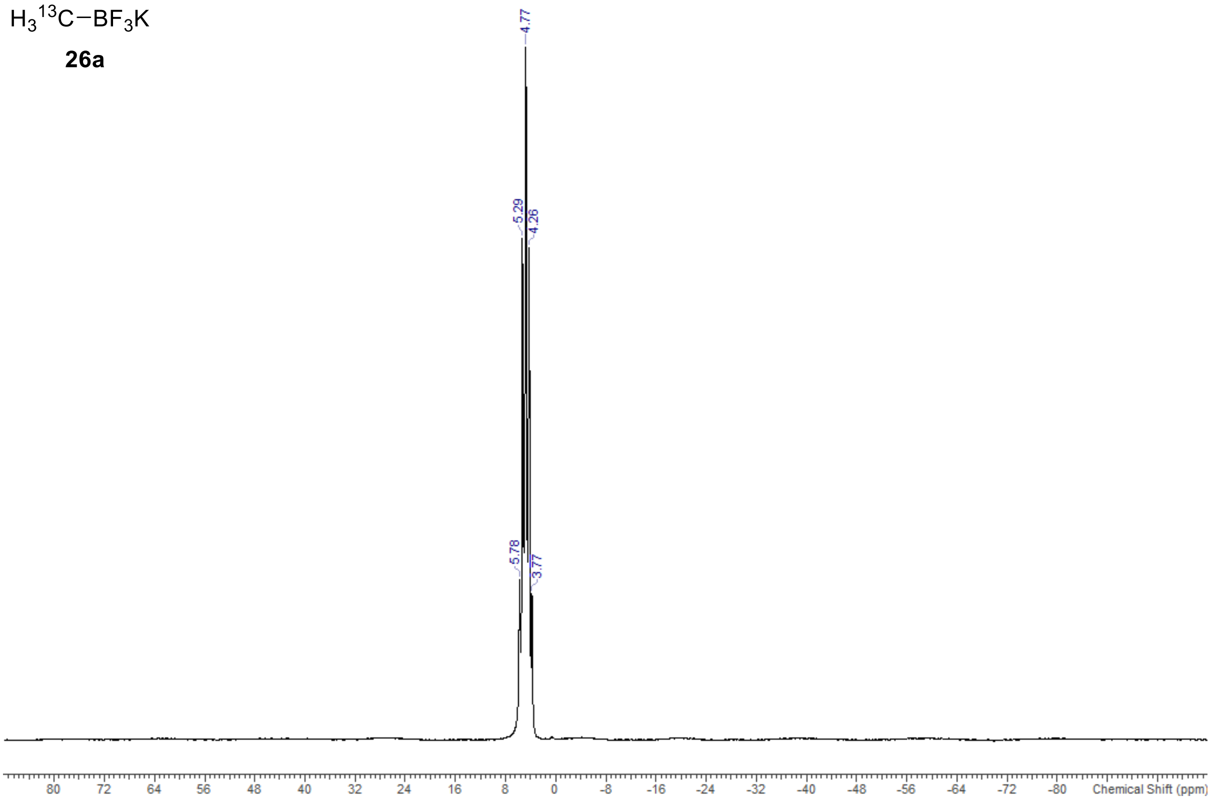

^{19}F NMR (376 MHz, DMSO-d_6) of **26a**

$H_3{}^{13}C-BF_3K$

26a

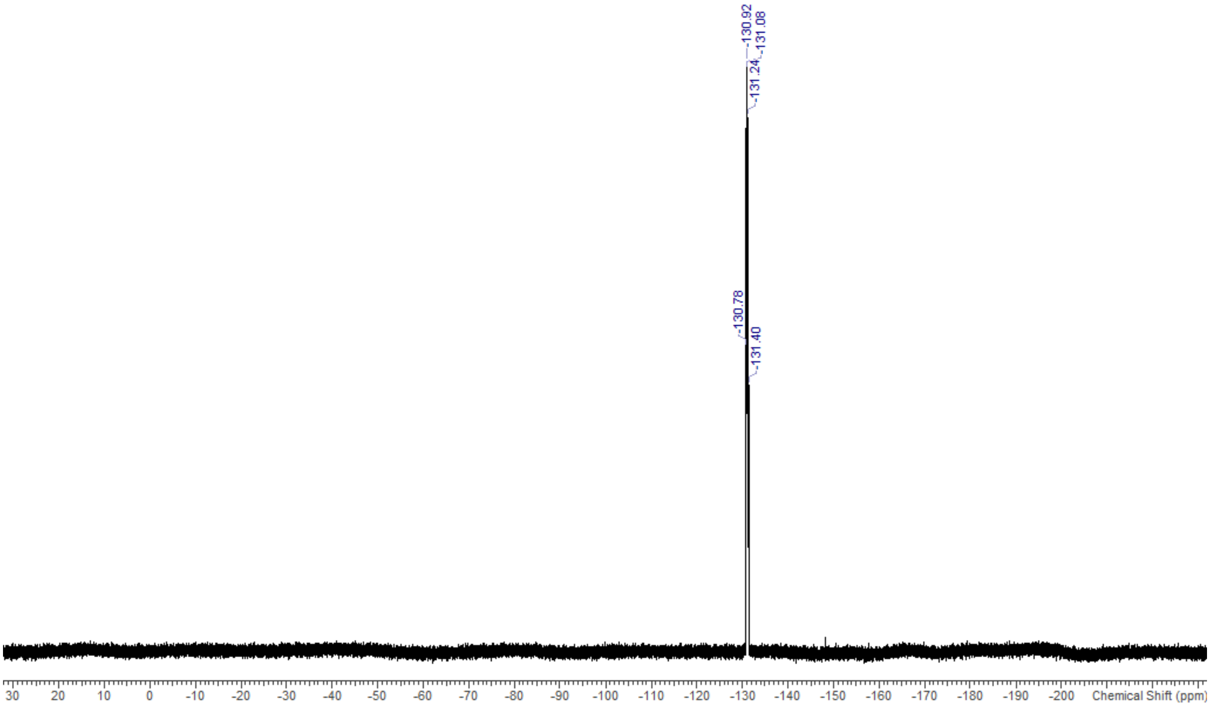

^{13}C NMR (100 MHz, DMSO-*d*6) of **26**

$D_3{}^{13}C-BF_3K$

26

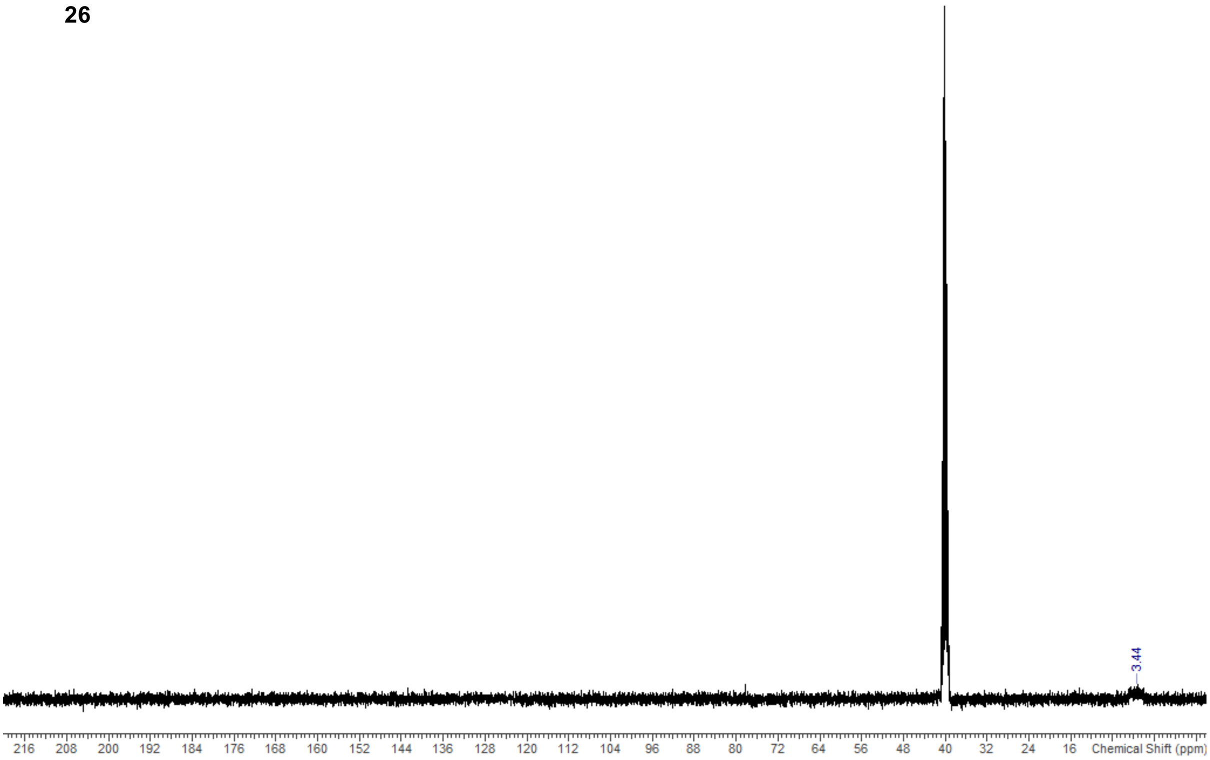

^{11}B NMR (128 MHz, DMSO-*d*6) of **26**

$D_3{}^{13}C-BF_3K$

26

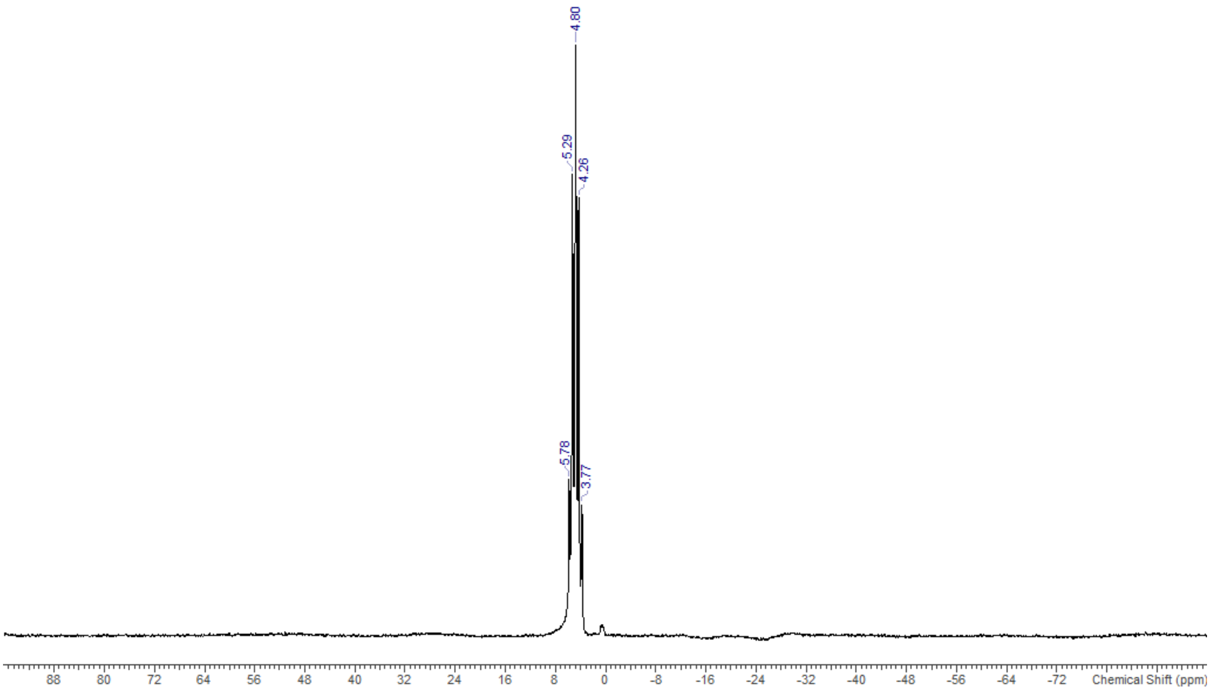

^{19}F NMR (376 MHz, DMSO-d_6) of **26**

$D_3{}^{13}C-BF_3K$

26

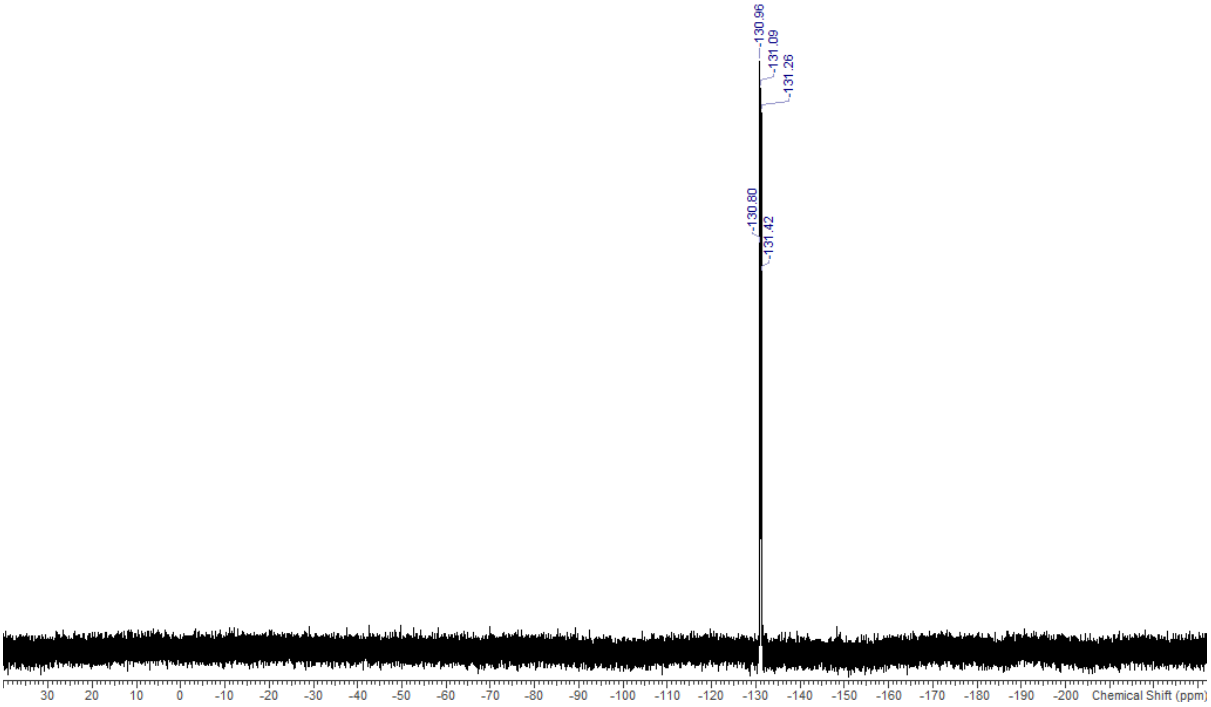

^{1}H NMR (400 MHz, $CDCl_3$) of **31a**

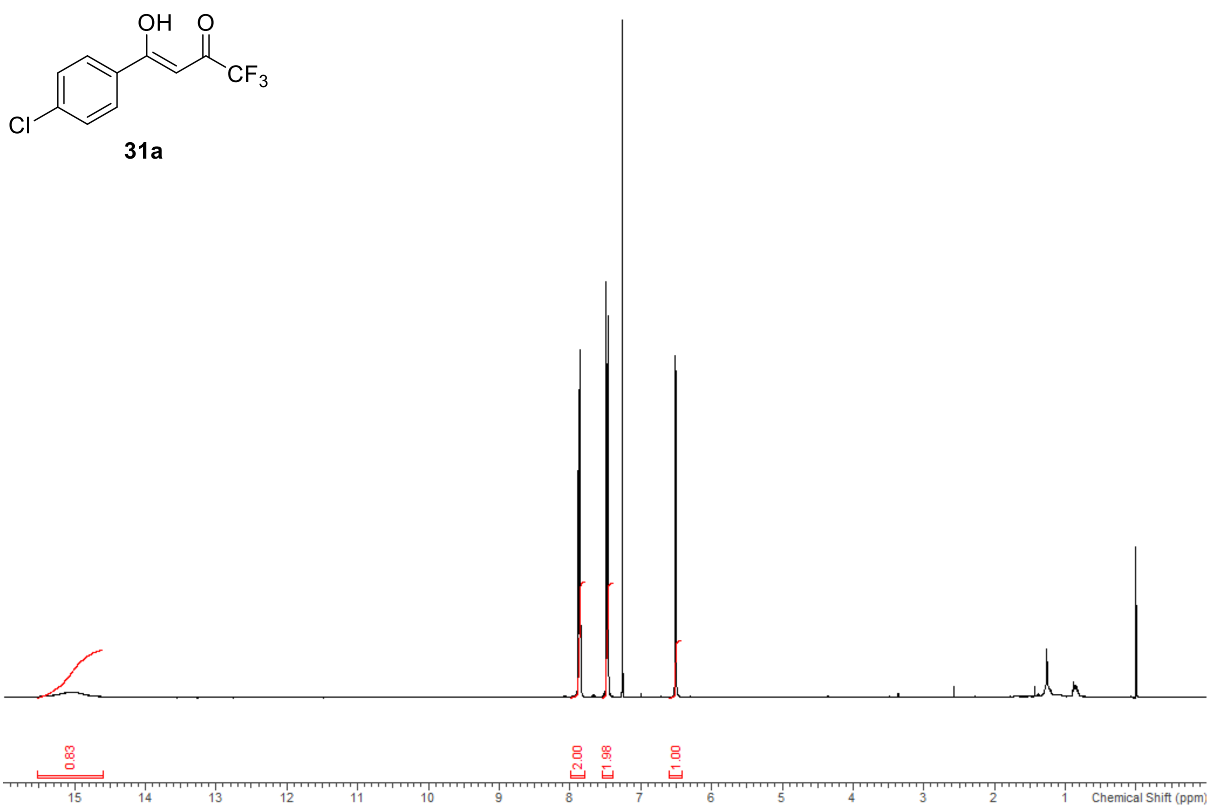

^{13}C NMR (100 MHz, $CDCl_3$) of **31a**

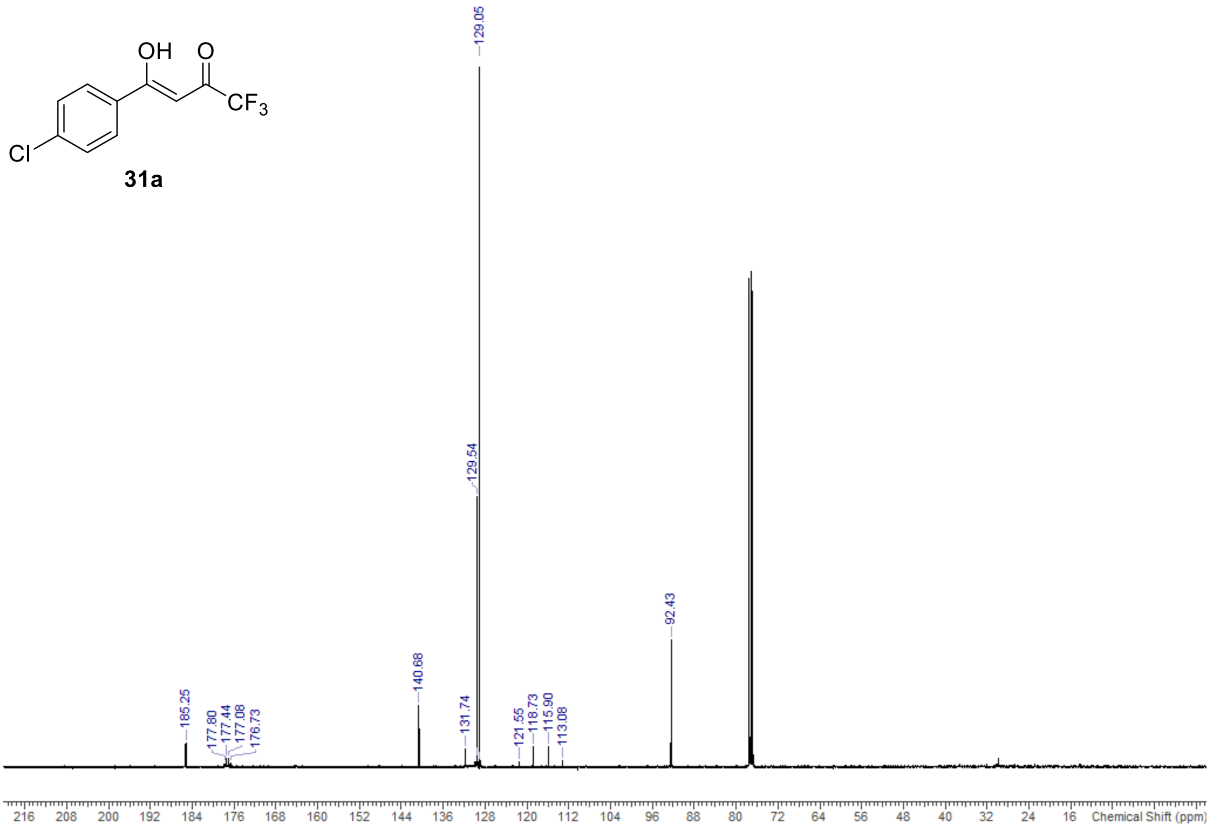

^{19}F NMR (376 MHz, $CDCl_3$) of **31a**

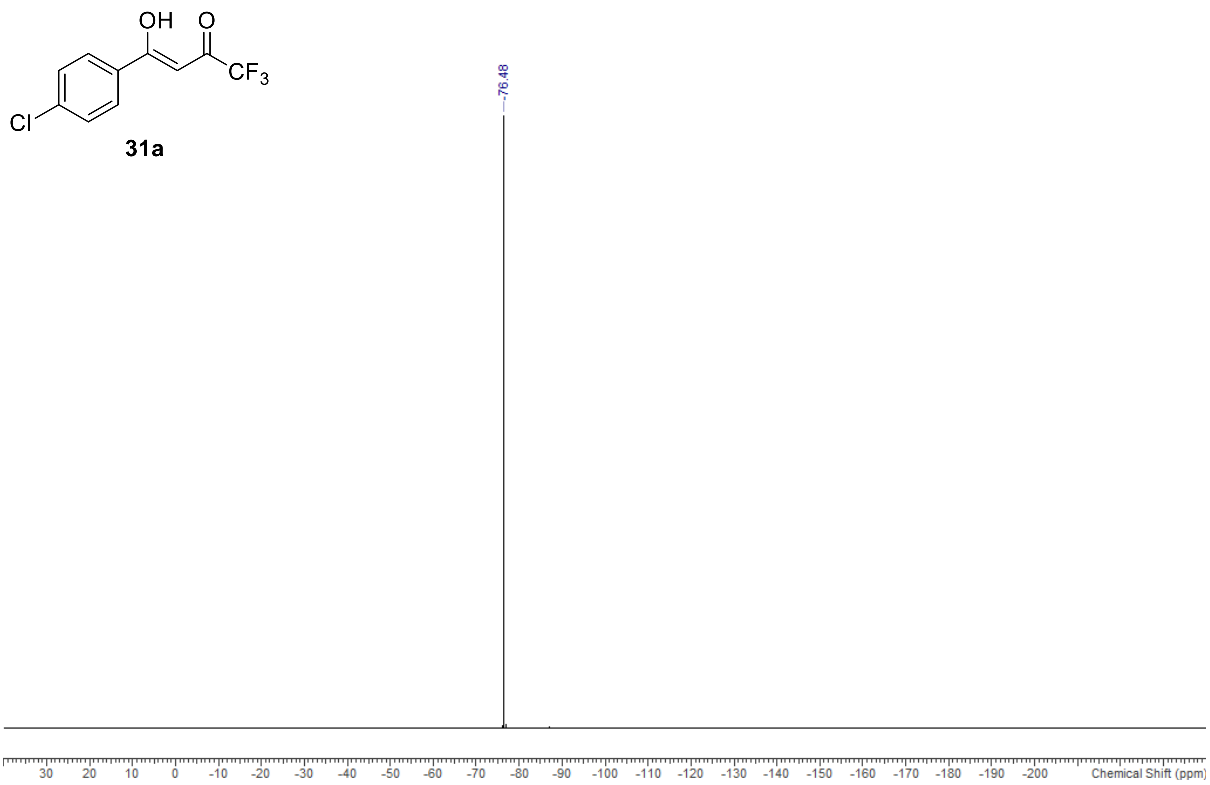

^{1}H NMR (400 MHz, DMSO-d_6) of **31b**

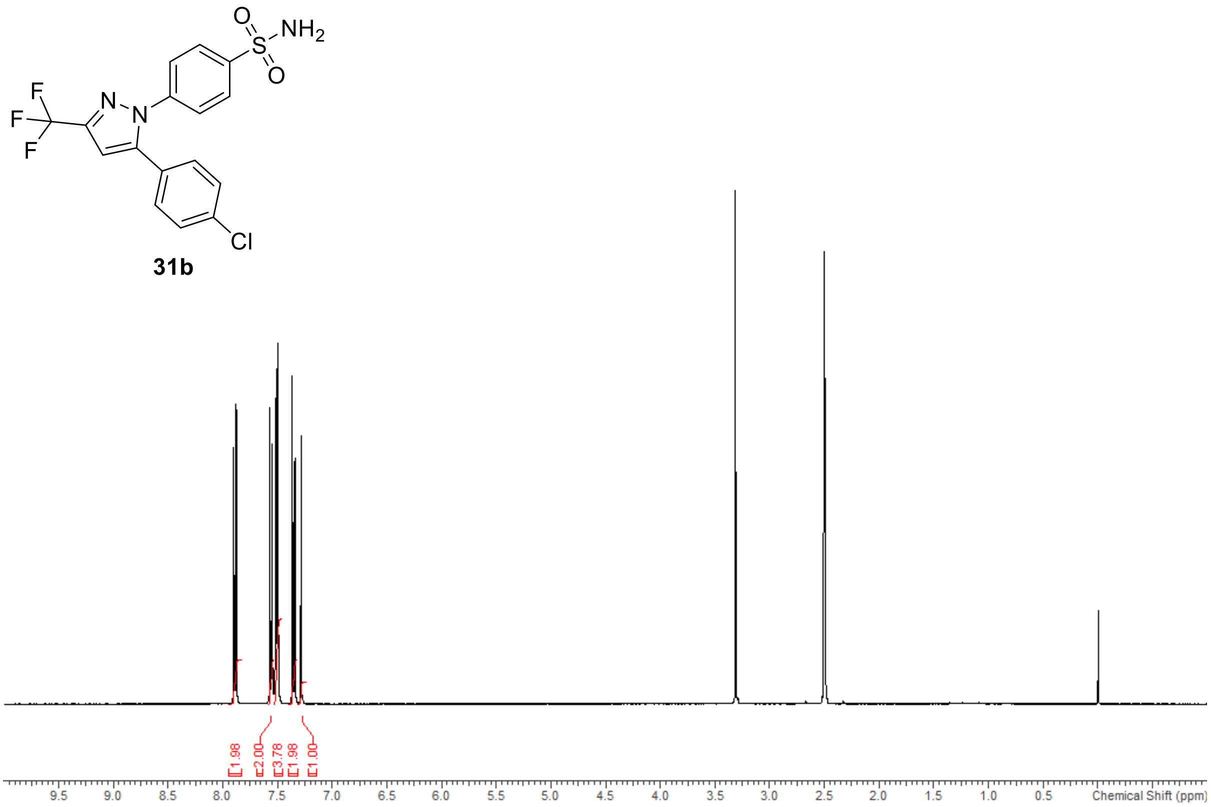

^{13}C NMR (100 MHz, DMSO-d_6) of **31b**

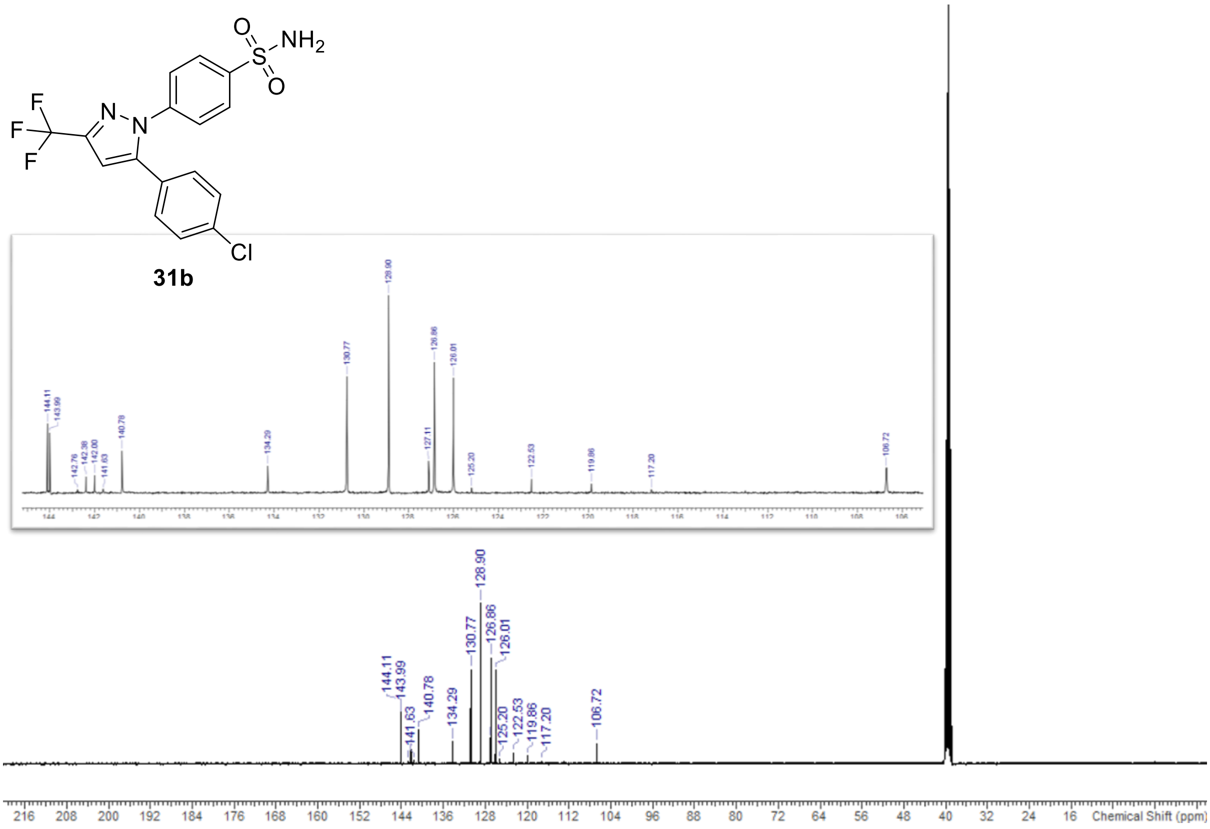

^{19}F NMR (376 MHz, DMSO-d_6) of **31b**

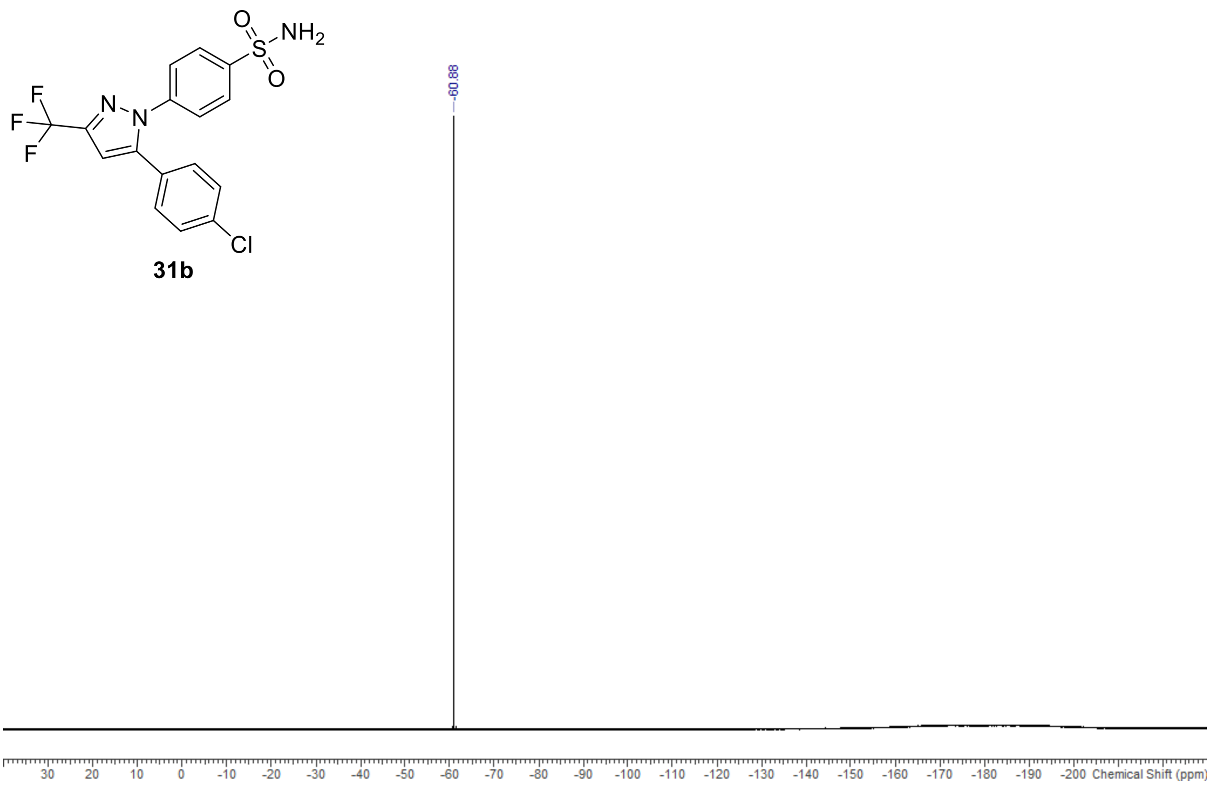

^{1}H NMR (400 MHz, $CDCl_3$) of **2a**

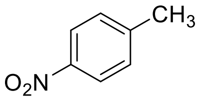

2a

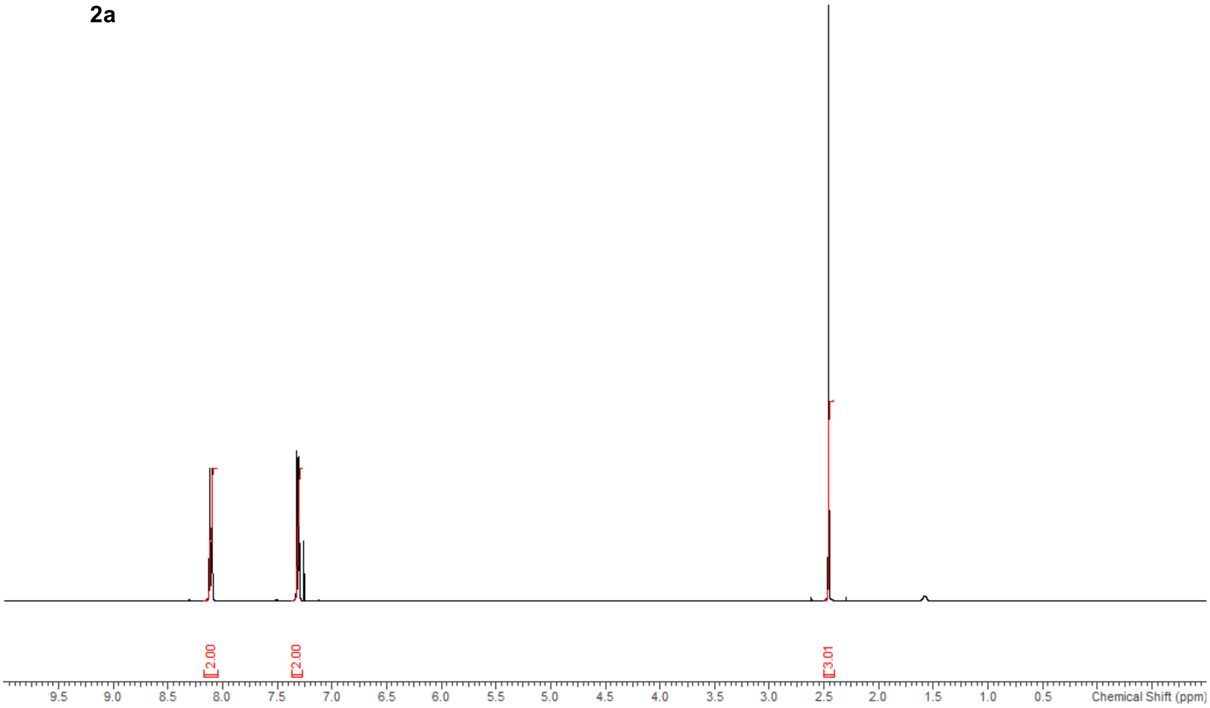

^{13}C NMR (100 MHz, $CDCl_3$) of **2a**

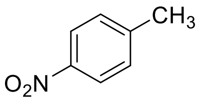

2a

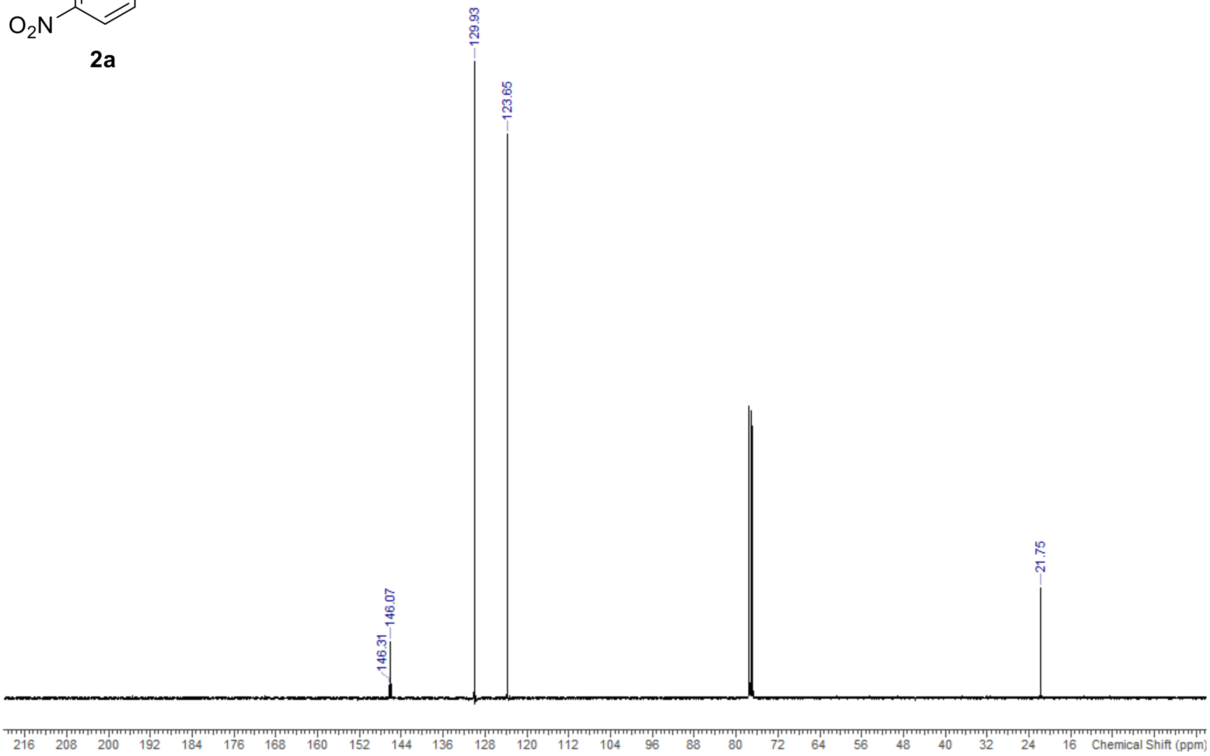

^{1}H NMR (400 MHz, $CDCl_3$) of **5a**

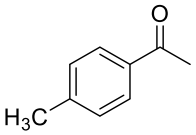

5a

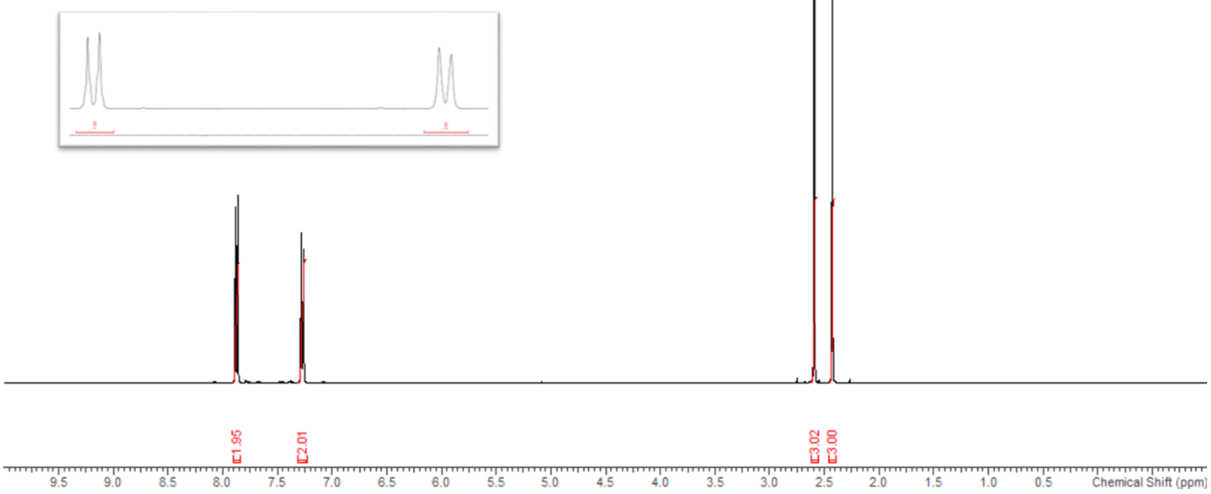

^{13}C NMR (100 MHz, $CDCl_3$) of **5a**

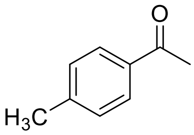

5a

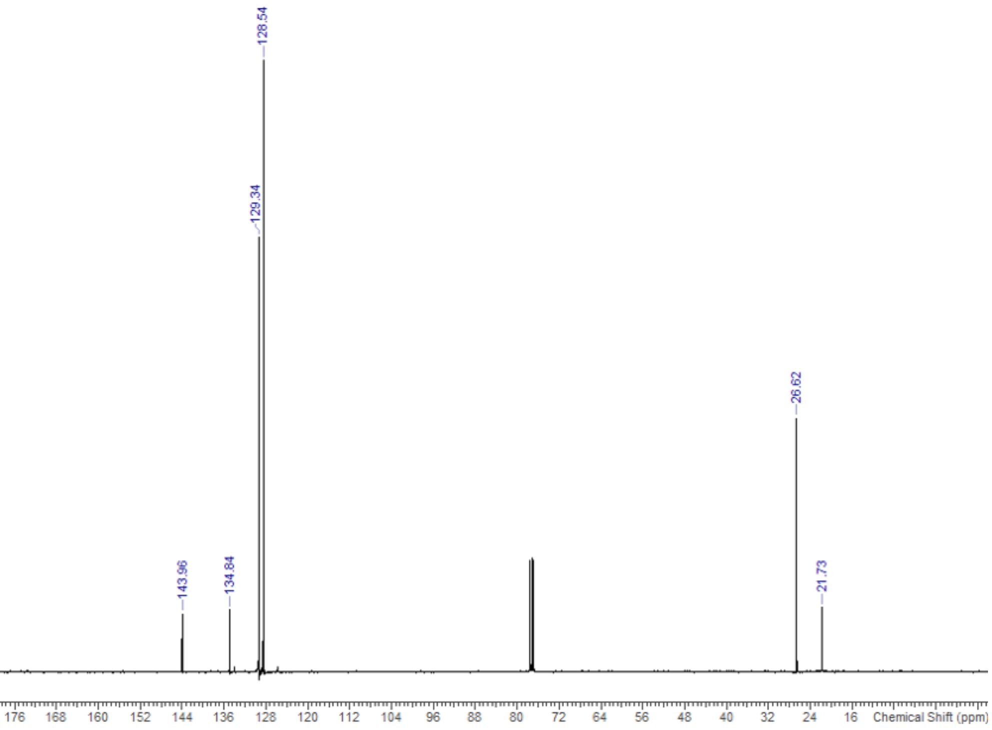

^{1}H NMR (400 MHz, $CDCl_3$) of **6a**

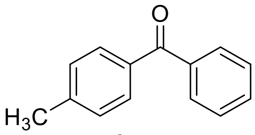

6a

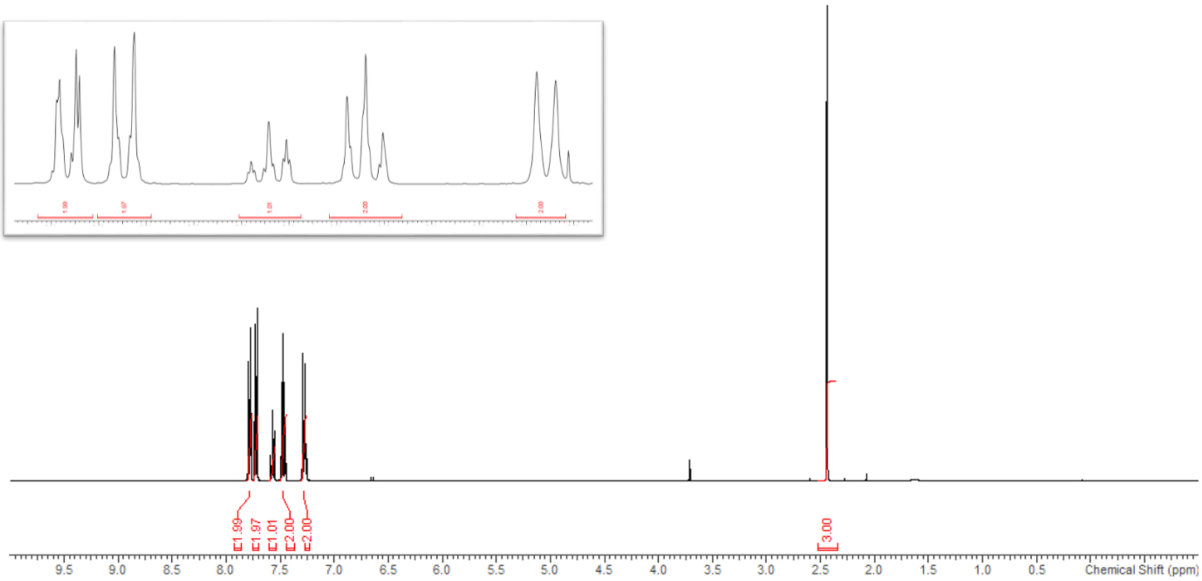

^{13}C NMR (100 MHz, $CDCl_3$) of **6a**

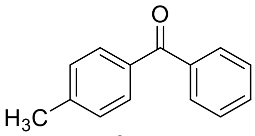

6a

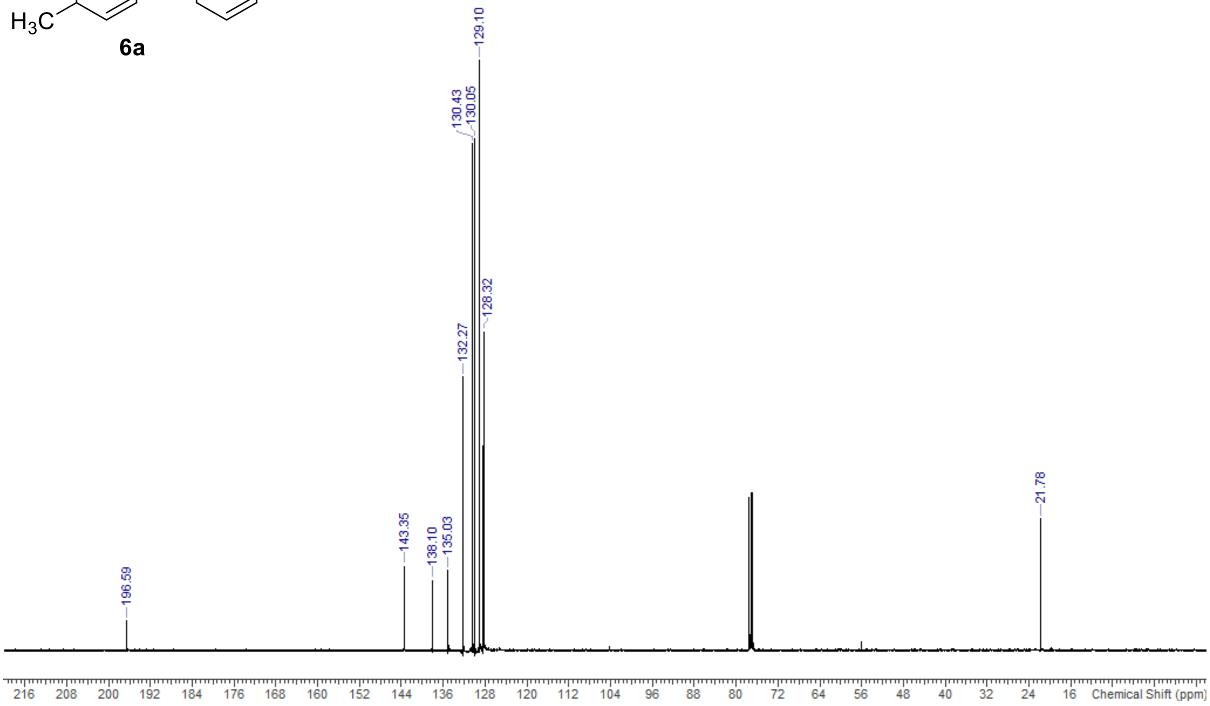

^{1}H NMR (400 MHz, $CDCl_3$) of **7a**

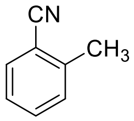

7a

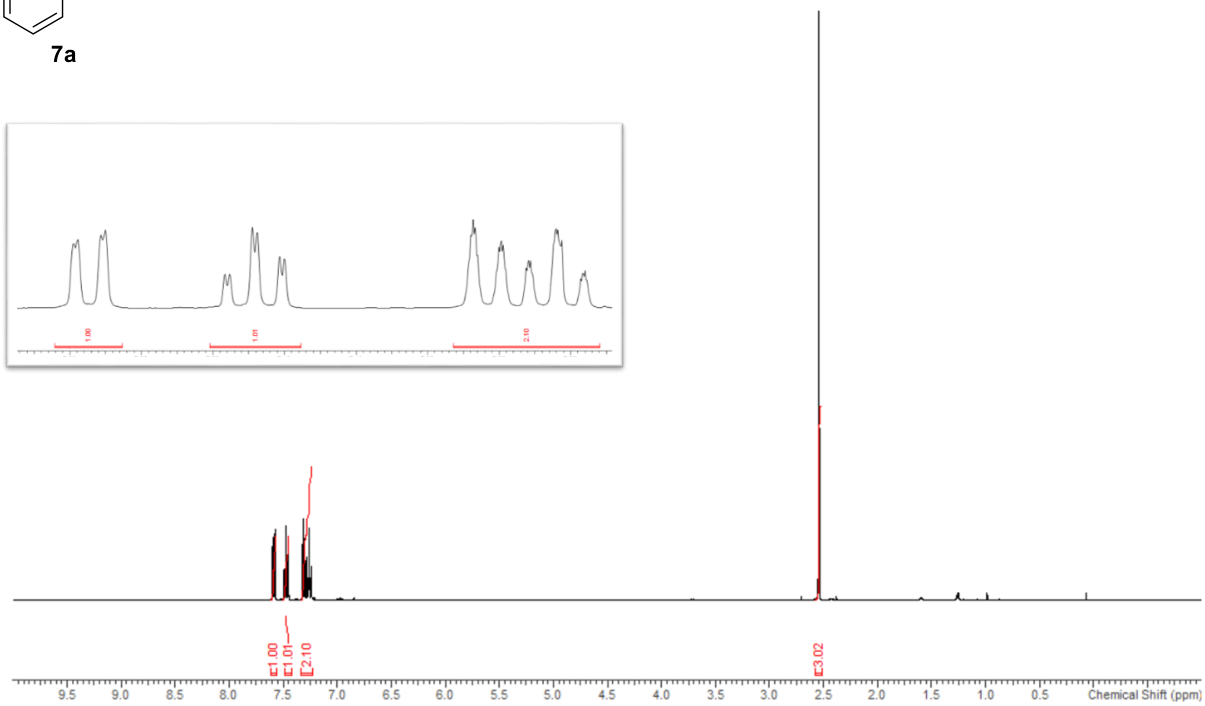

^{13}C NMR (100 MHz, $CDCl_3$) of **7a**

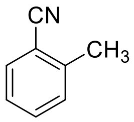

7a

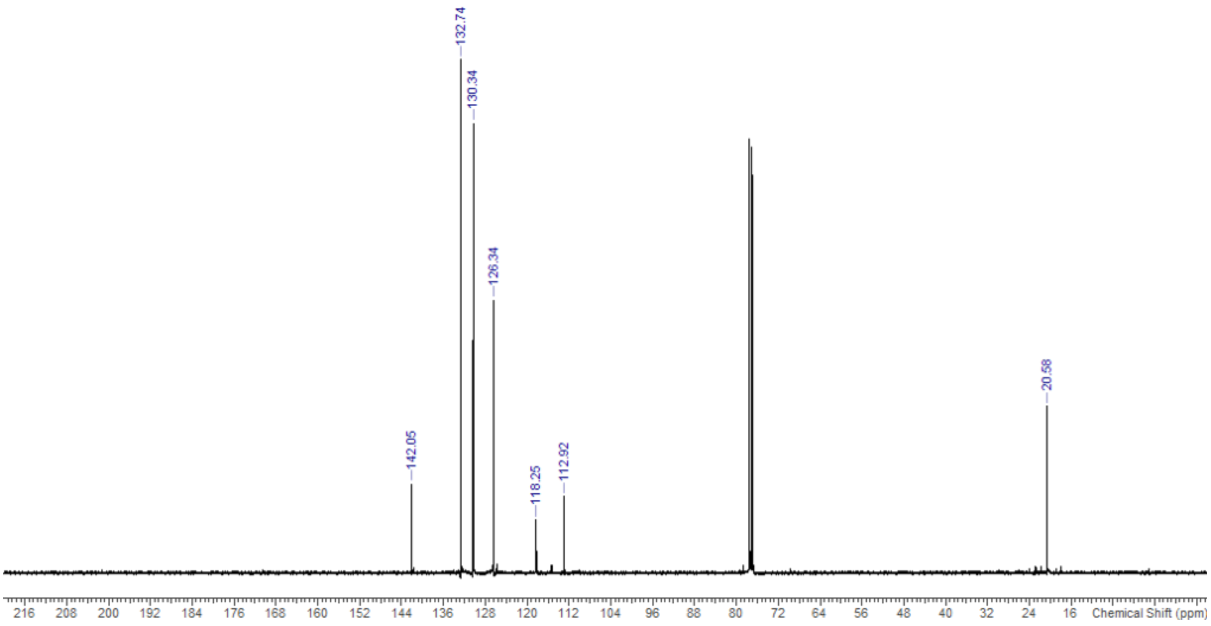

^{1}H NMR (400 MHz, DMSO-d_6) of **8a·HCl**

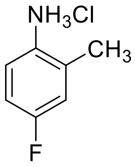

8a·HCl

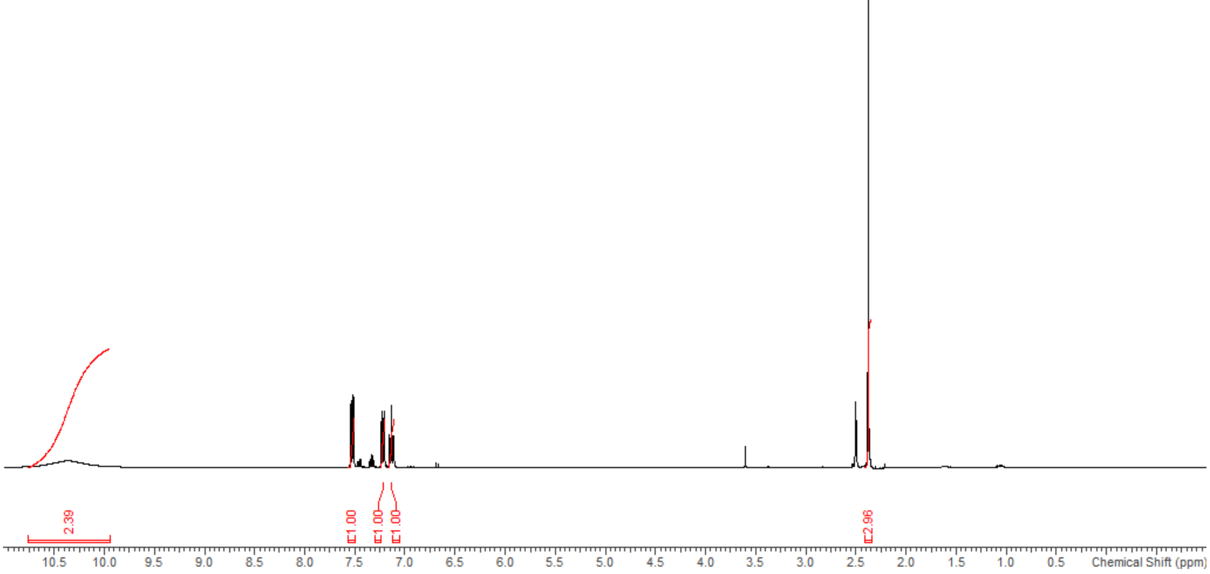

^{13}C NMR (100 MHz, DMSO-d_6) of **8a·HCl**

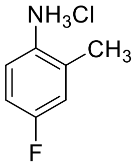

8a·HCl

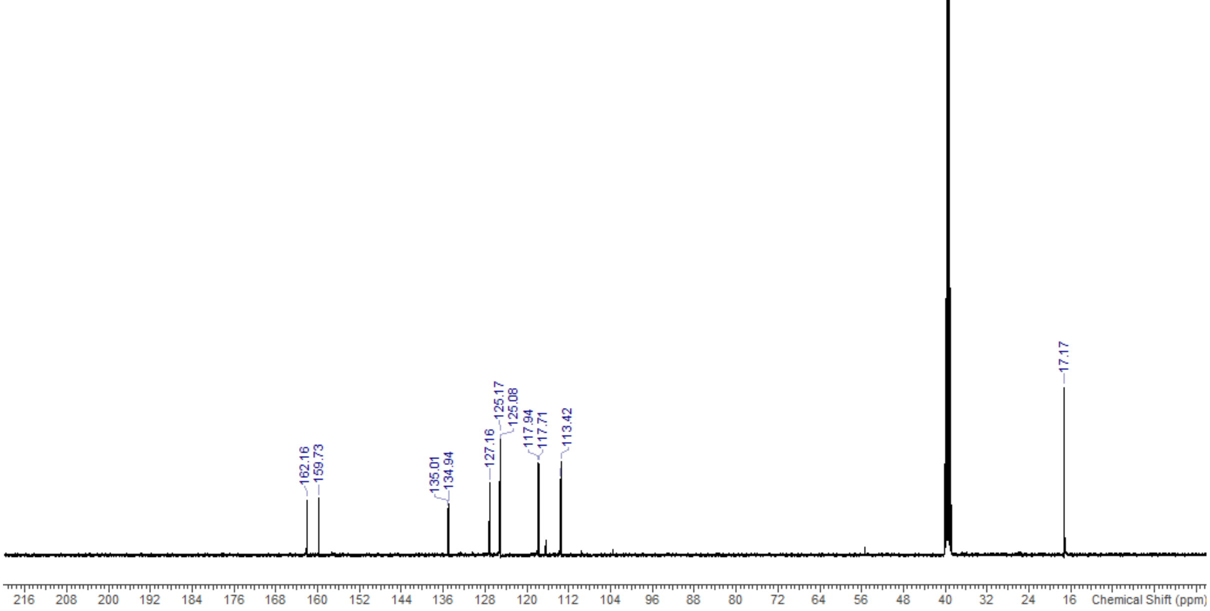

^{19}F NMR (376 MHz, DMSO-d_6) of **8a·HCl**

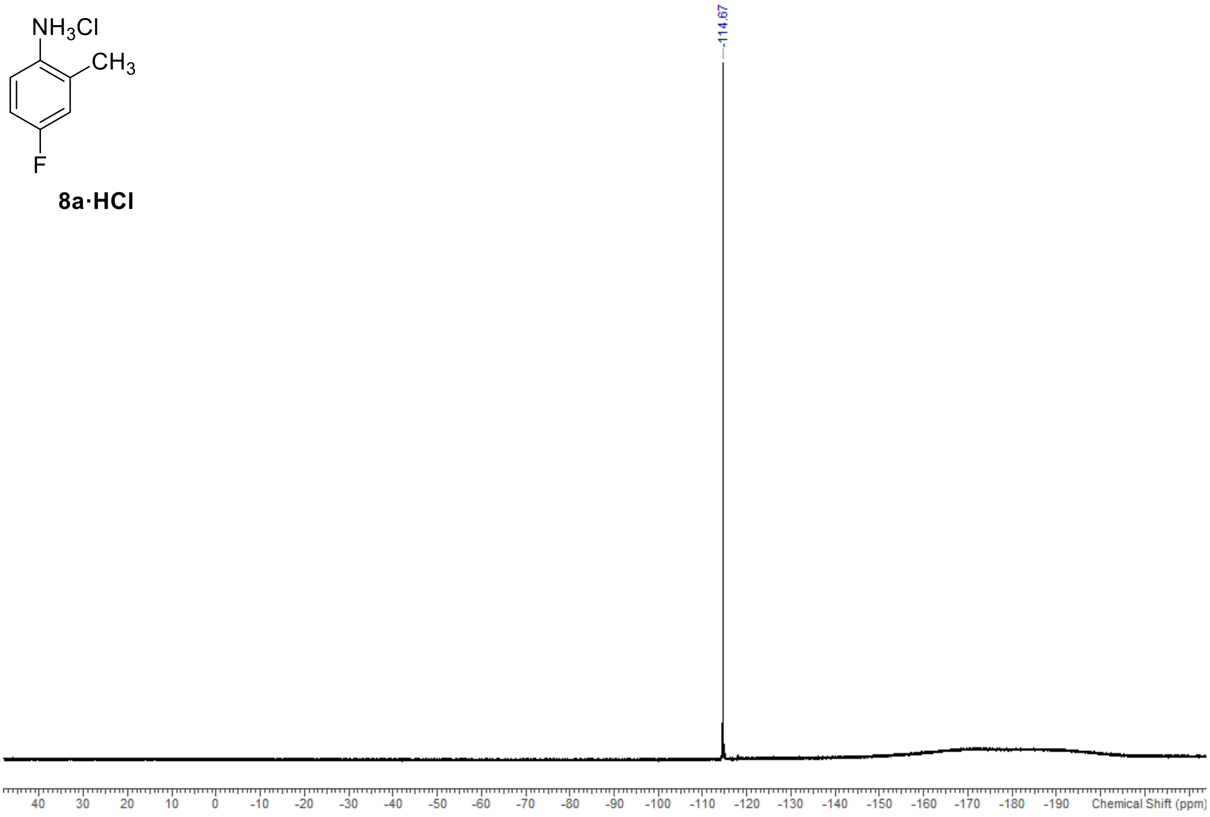

^{1}H NMR (400 MHz, $CDCl_3$) of **9a**

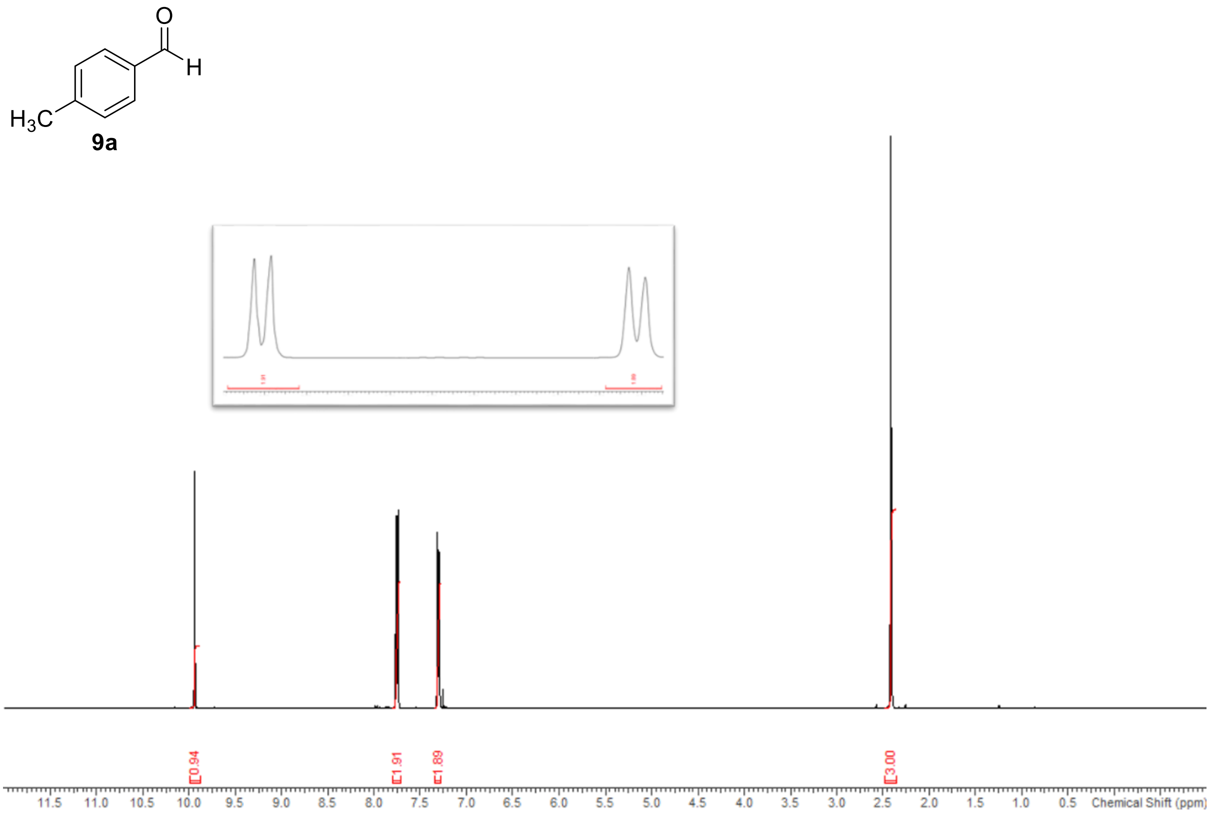

^{13}C NMR (100 MHz, $CDCl_3$) of **9a**

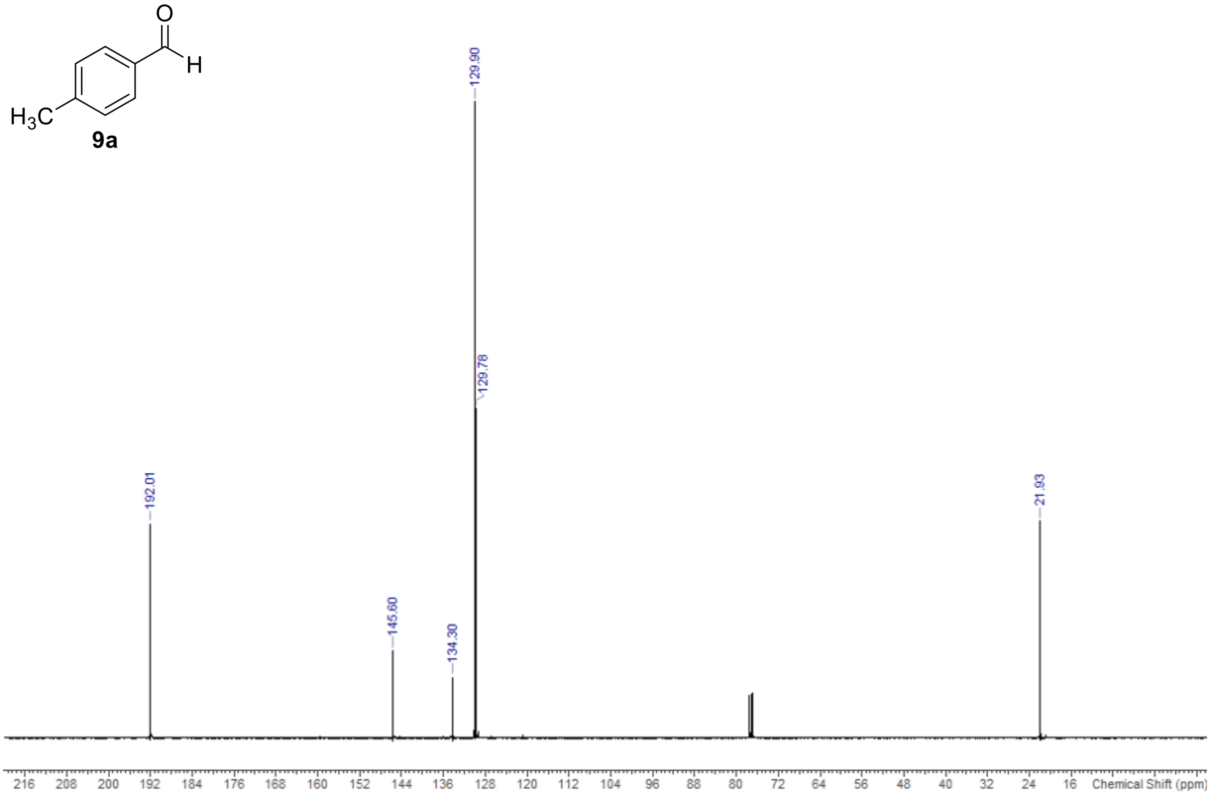

^{1}H NMR (400 MHz, $CDCl_3$) of **10a**

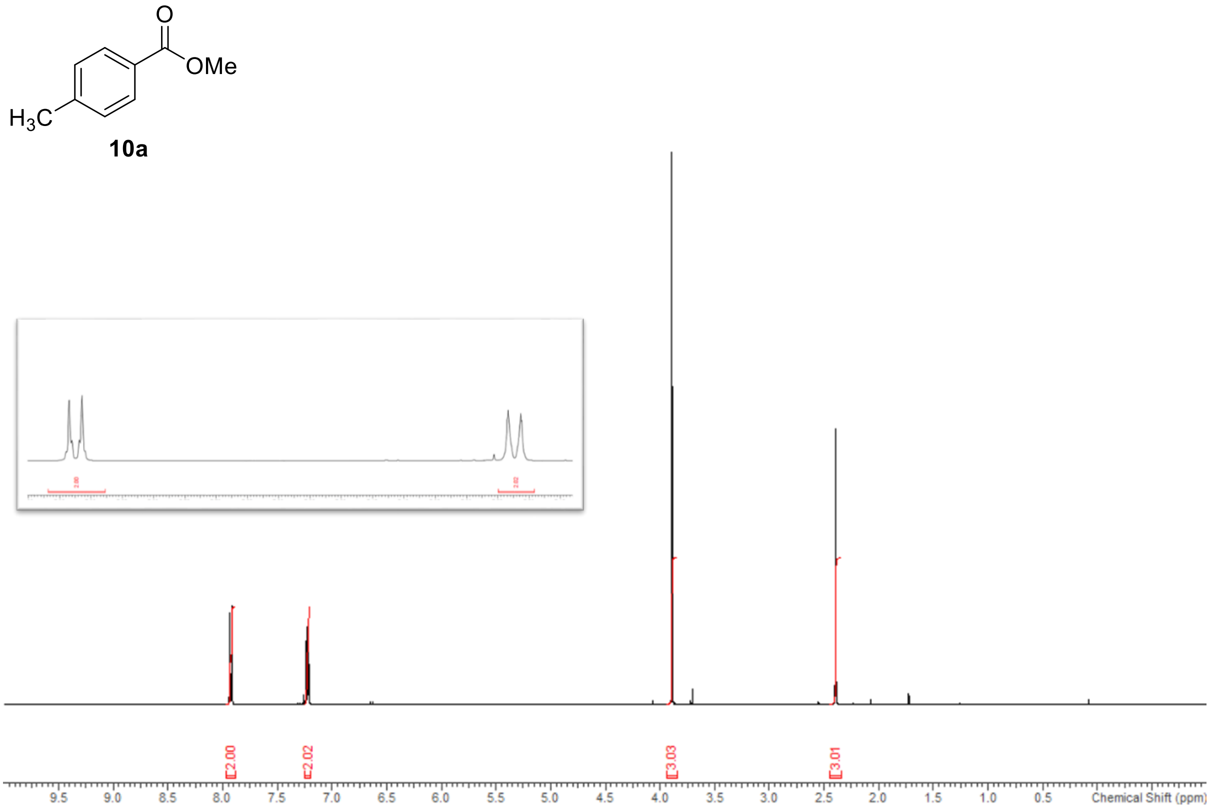

^{13}C NMR (100 MHz, $CDCl_3$) of **10a**

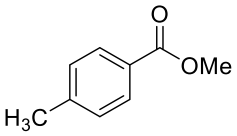

10a

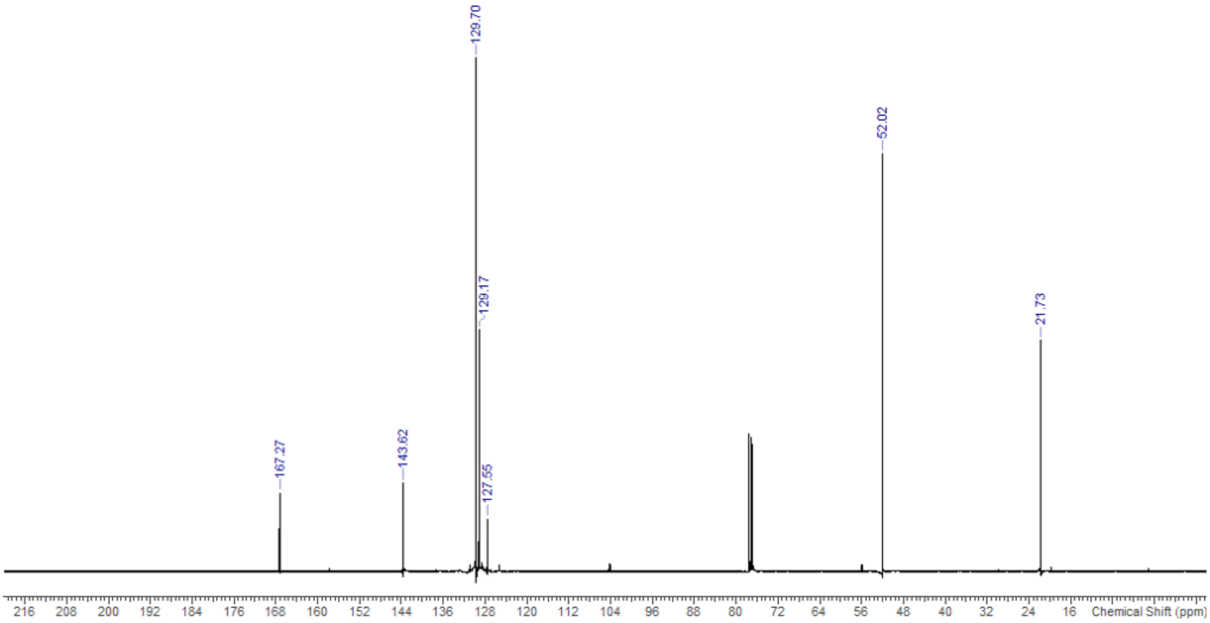

^{19}F NMR (376 MHz, $CDCl_3$) of **11a**

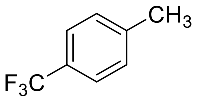

11a

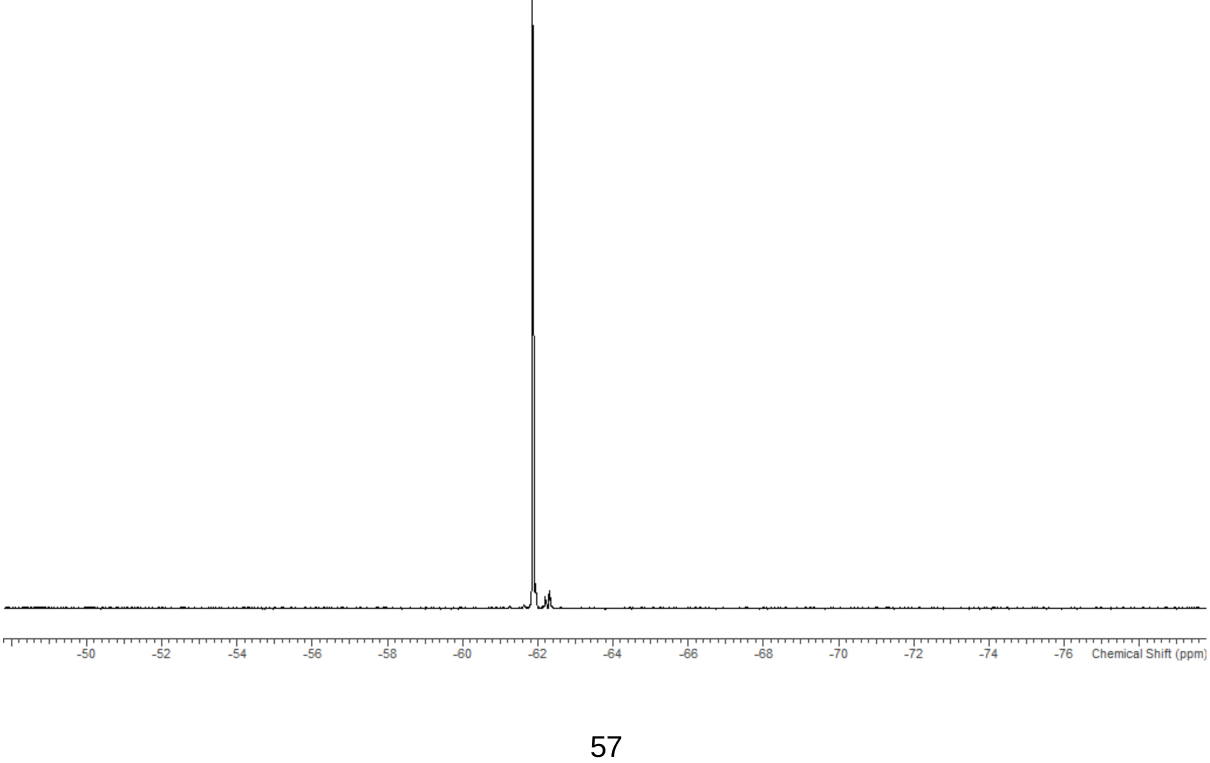

^{1}H NMR (400 MHz, $CDCl_3$) of **13a**

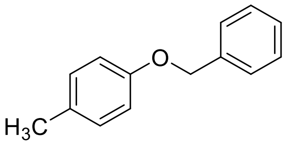

13a

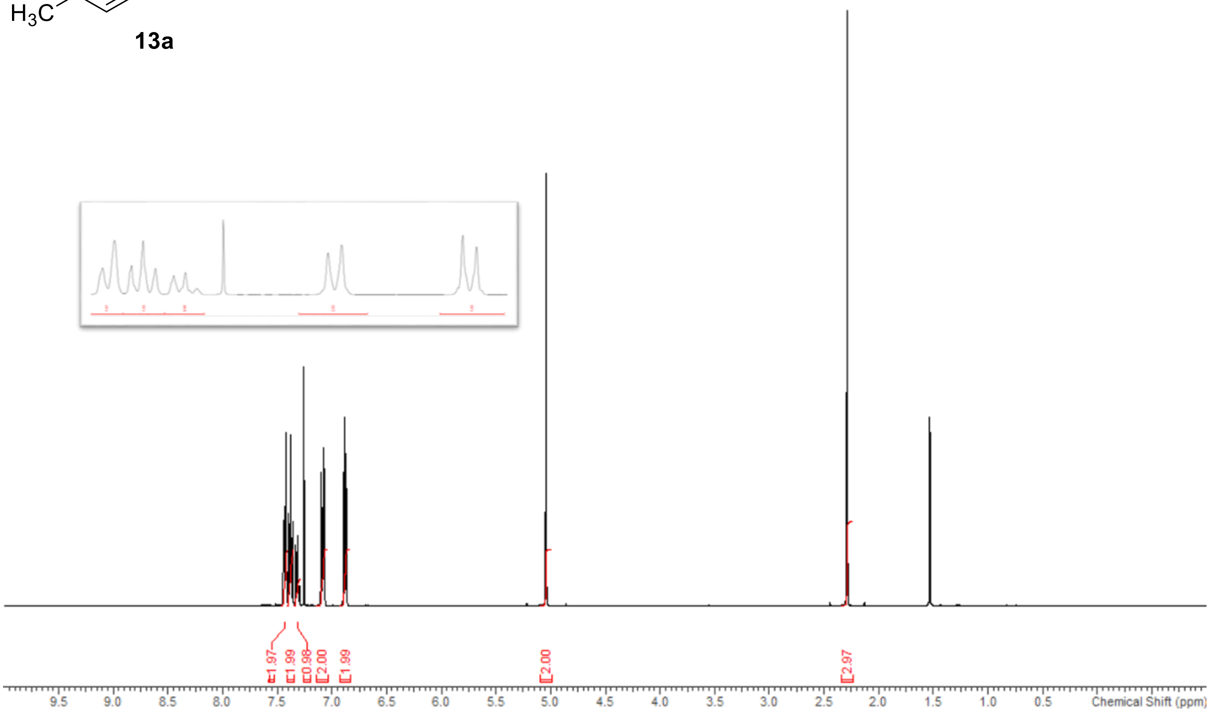

^{13}C NMR (100 MHz, $CDCl_3$) of **13a**

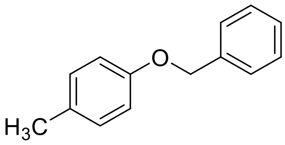

13a

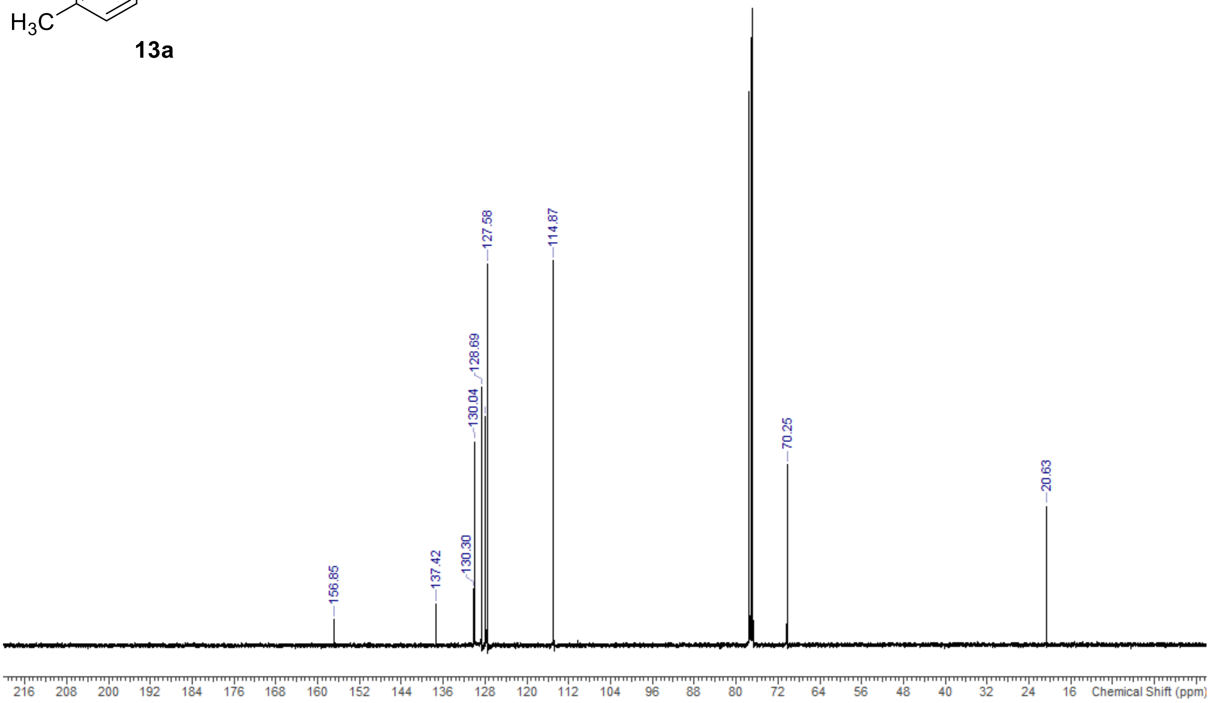

^{1}H NMR (400 MHz, $CDCl_3$) of **14a**

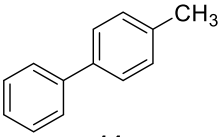

14a

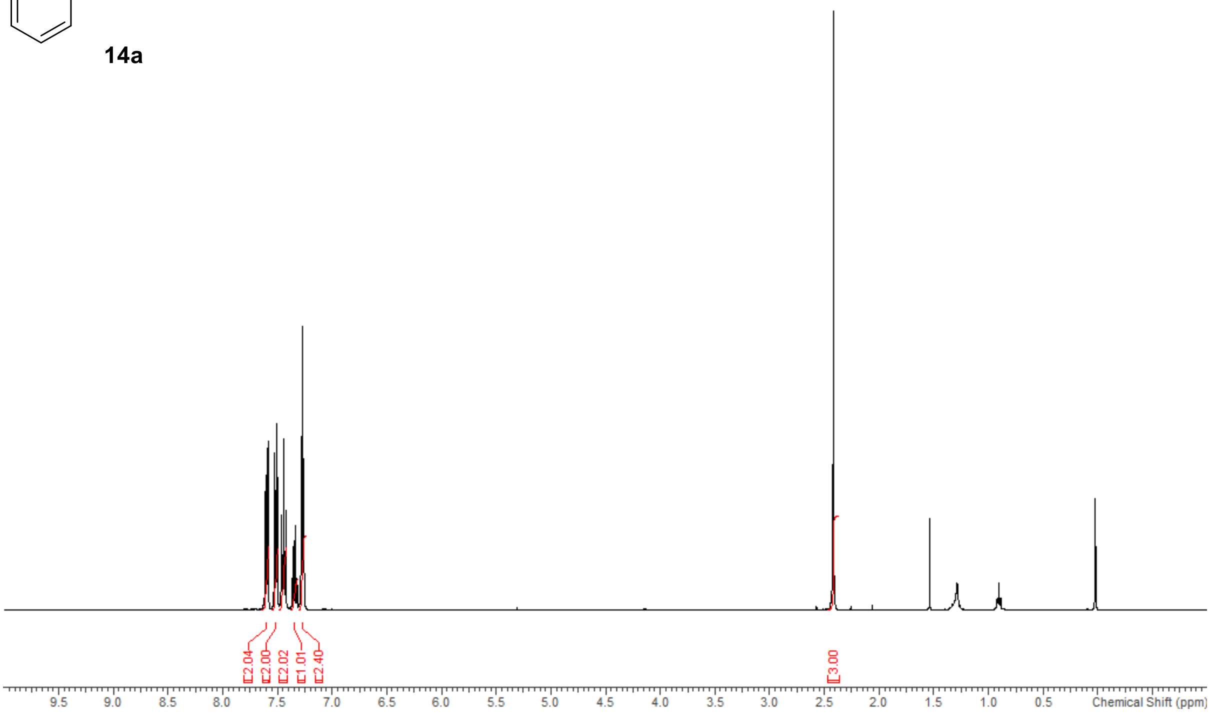

^{13}C NMR (100 MHz, $CDCl_3$) of **14a**

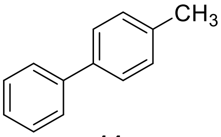

14a

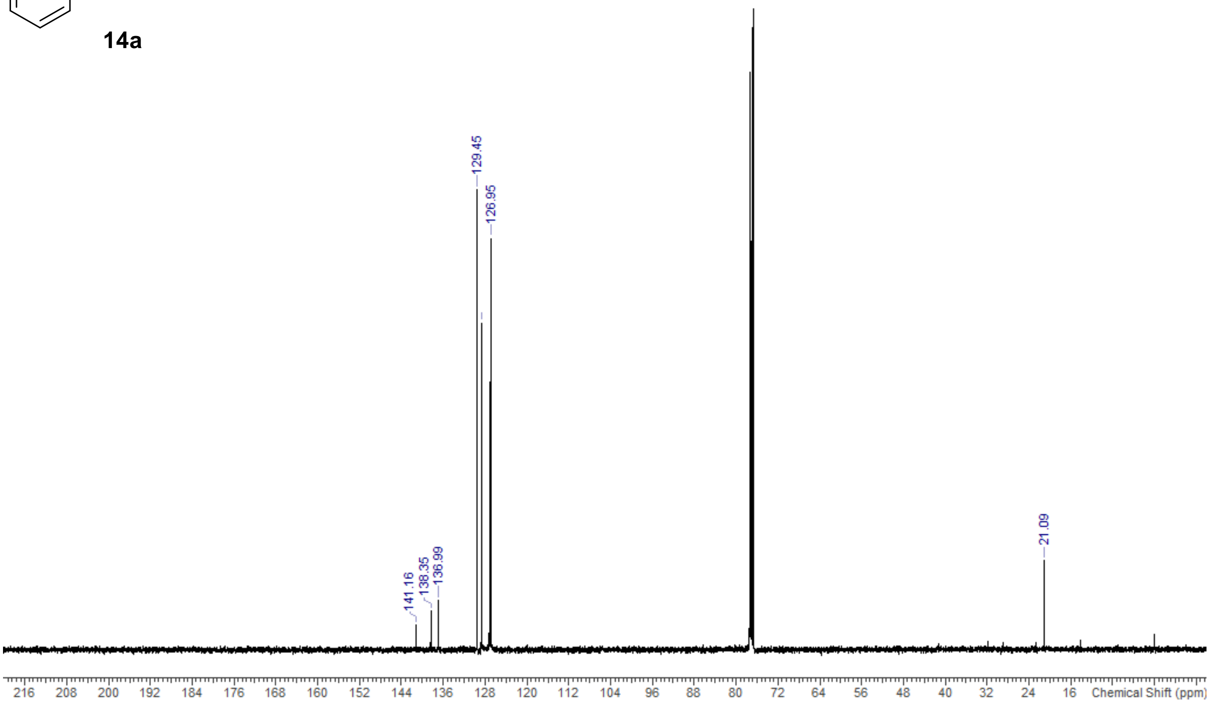

^{1}H NMR (400 MHz, $CDCl_3$) of **17a**

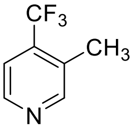

17a

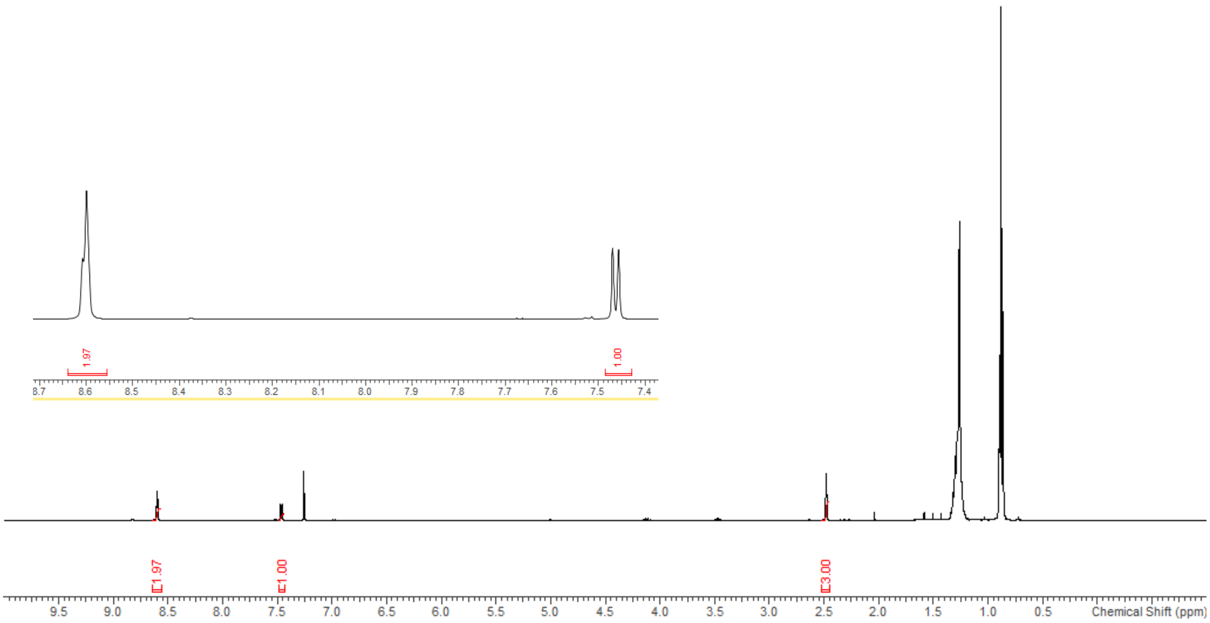

^{13}C NMR (100 MHz, $CDCl_3$) of **17a**

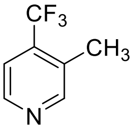

17a

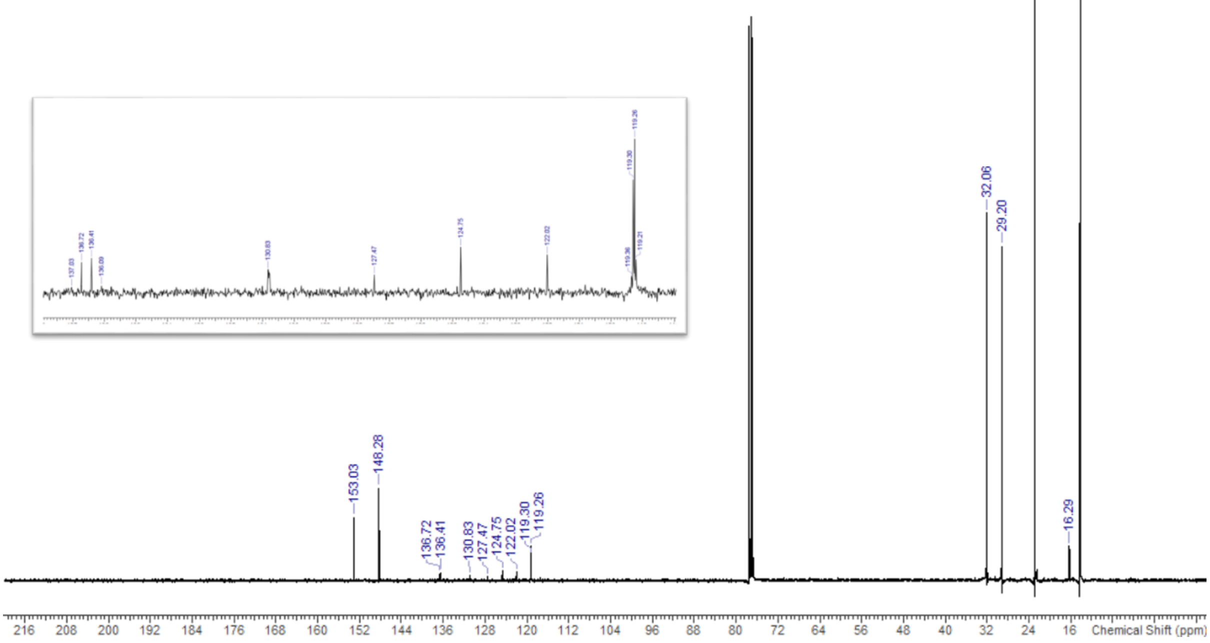

^{19}F NMR (376 MHz, $CDCl_3$) of **17a**

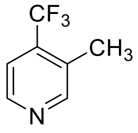

17a

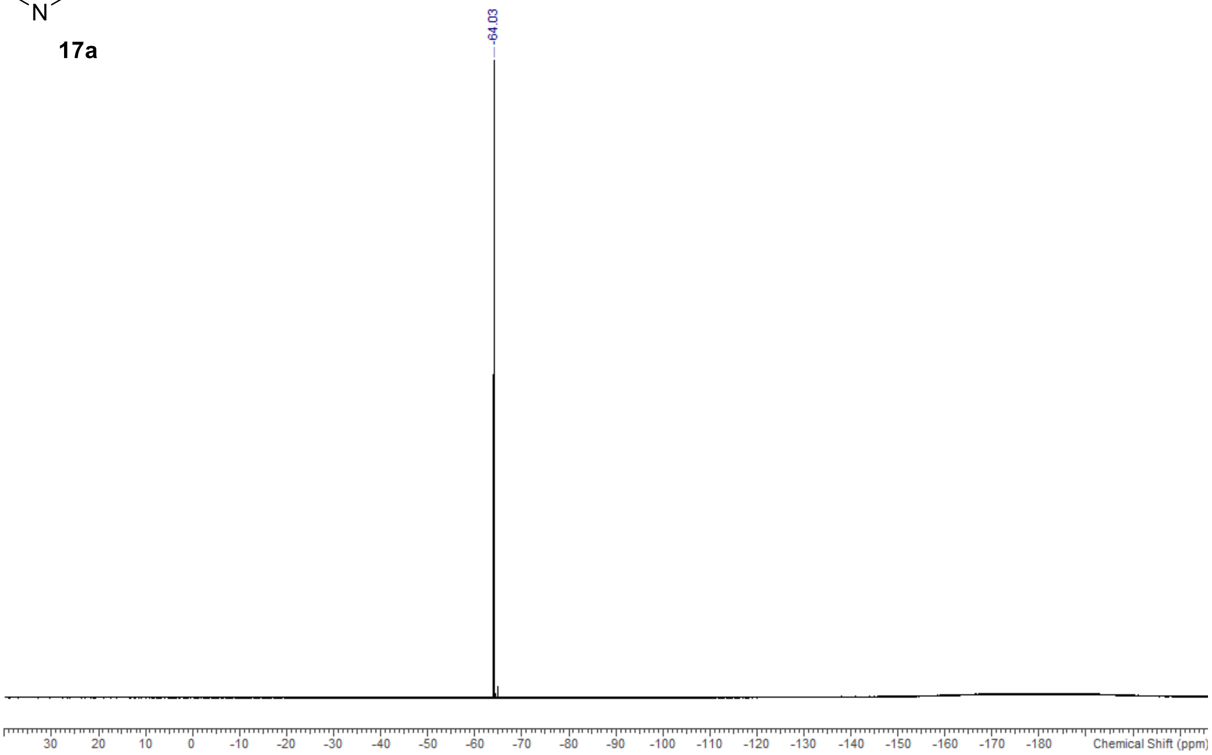

^{1}H NMR (400 MHz, $CDCl_3$) of **18a**

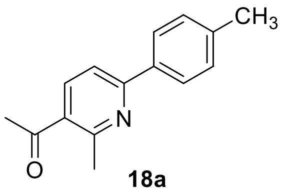

18a

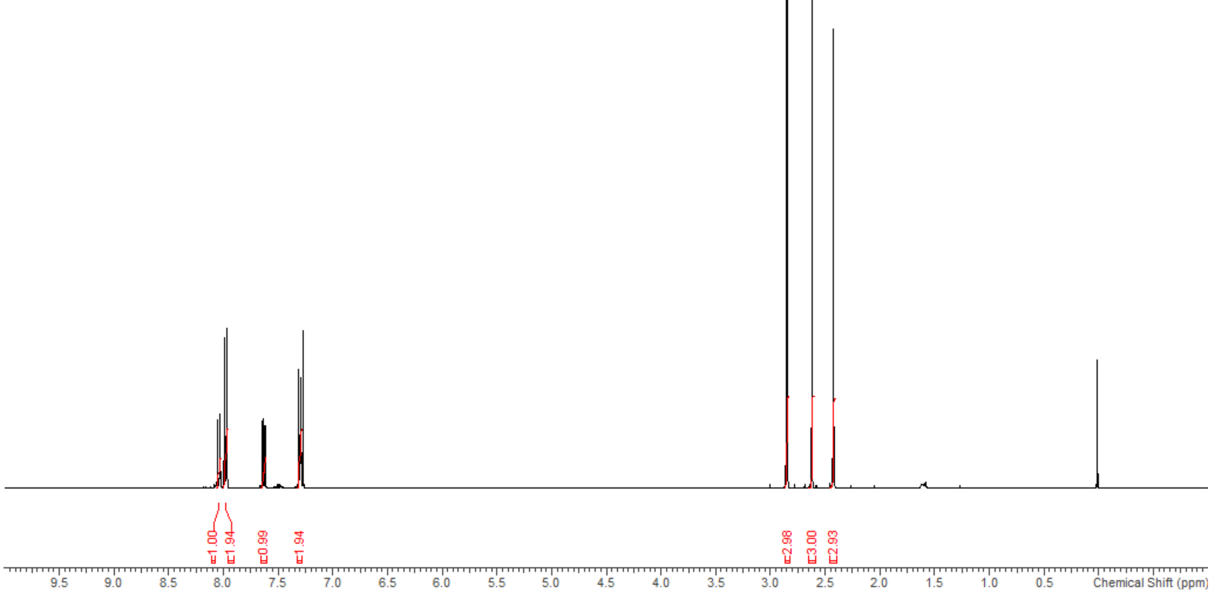

^{13}C NMR (100 MHz, $CDCl_3$) of **18a**

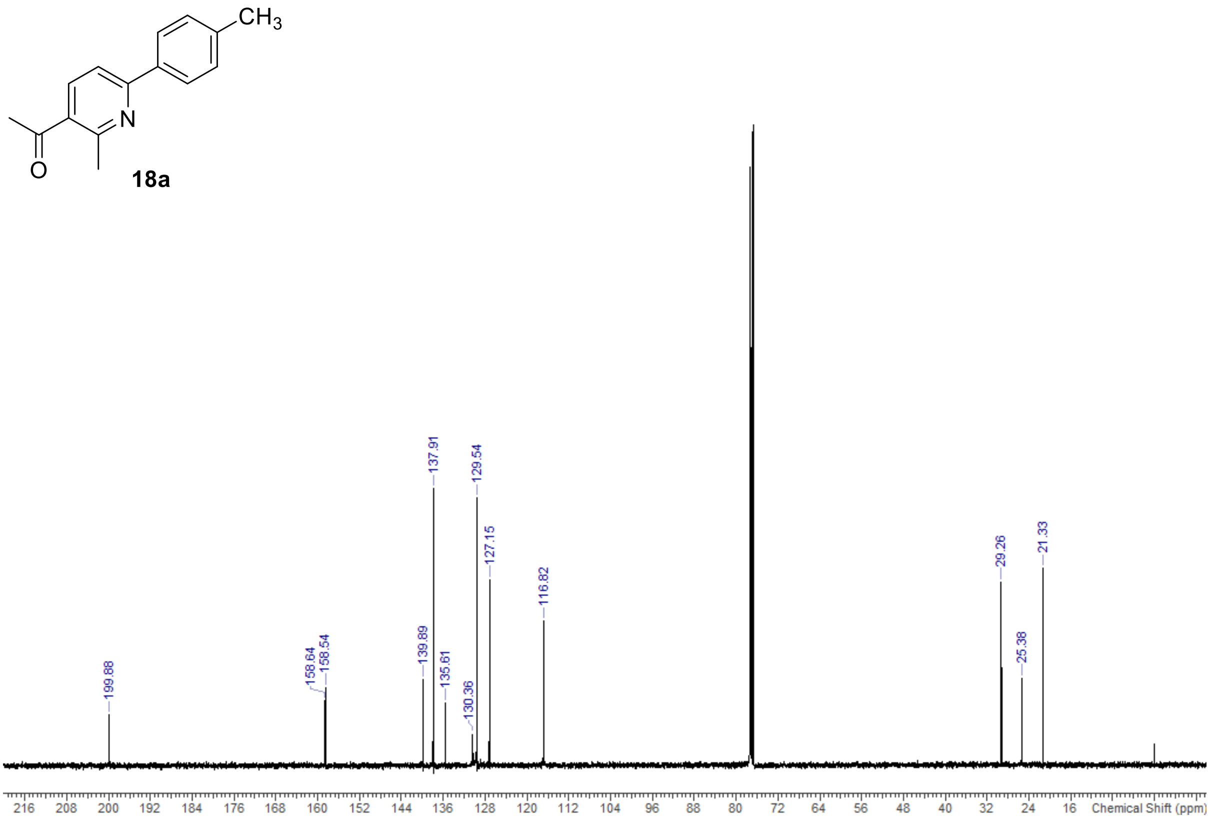

^{1}H NMR (400 MHz, $CDCl_3$) of **19a**

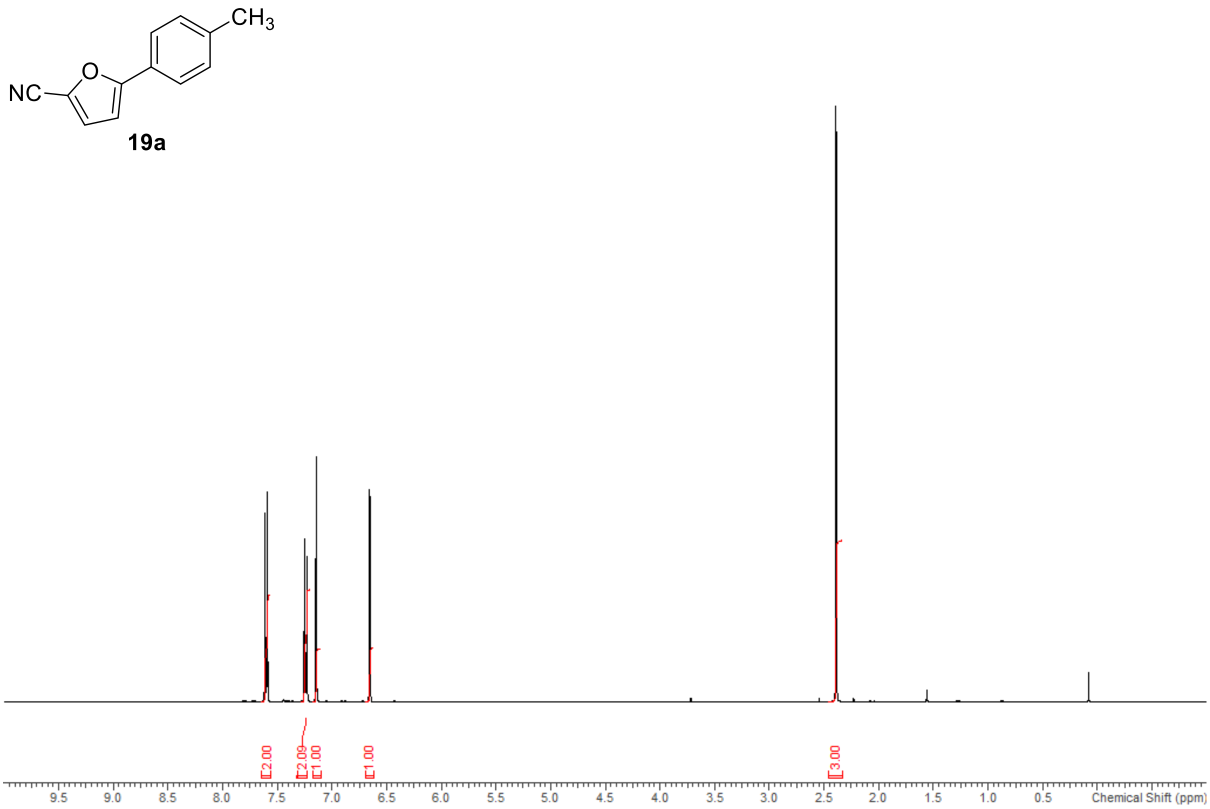

^{13}C NMR (100 MHz, $CDCl_3$) of **19a**

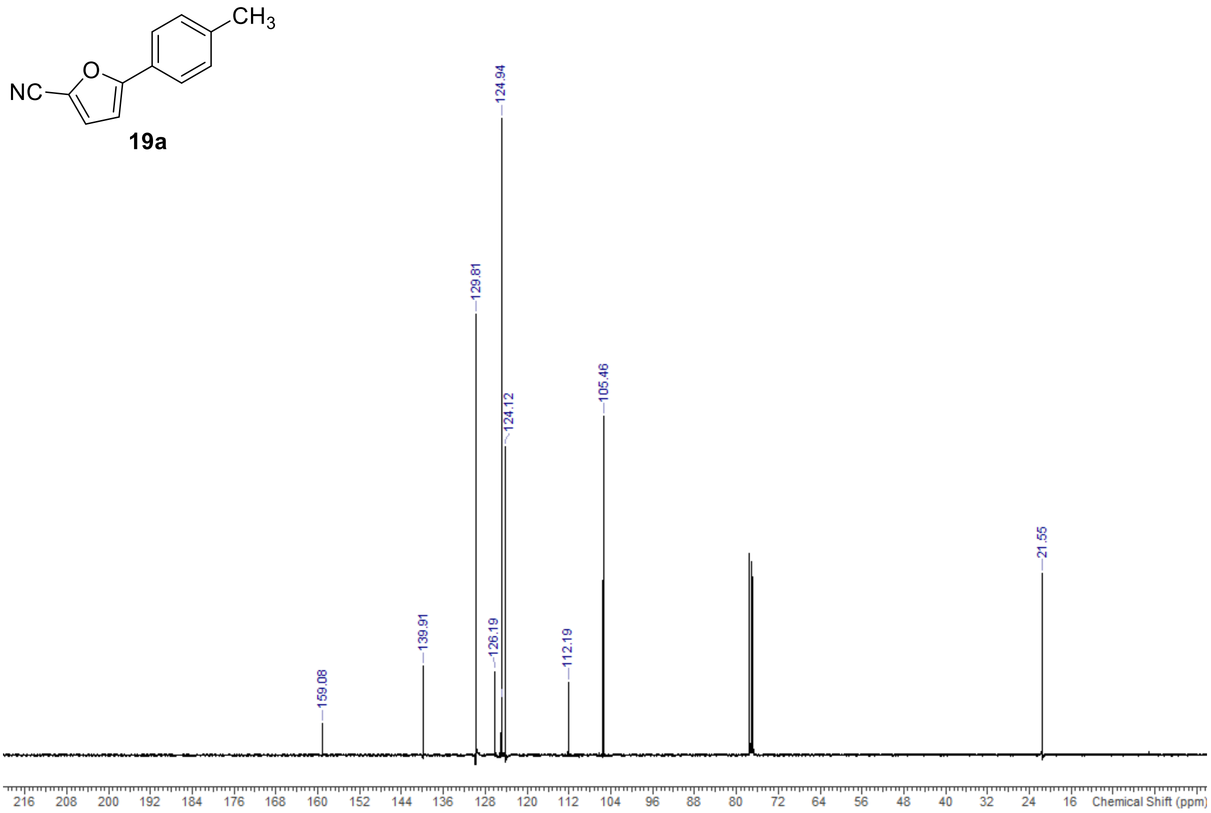

^{1}H NMR (400 MHz, $CDCl_3$) of **20a**

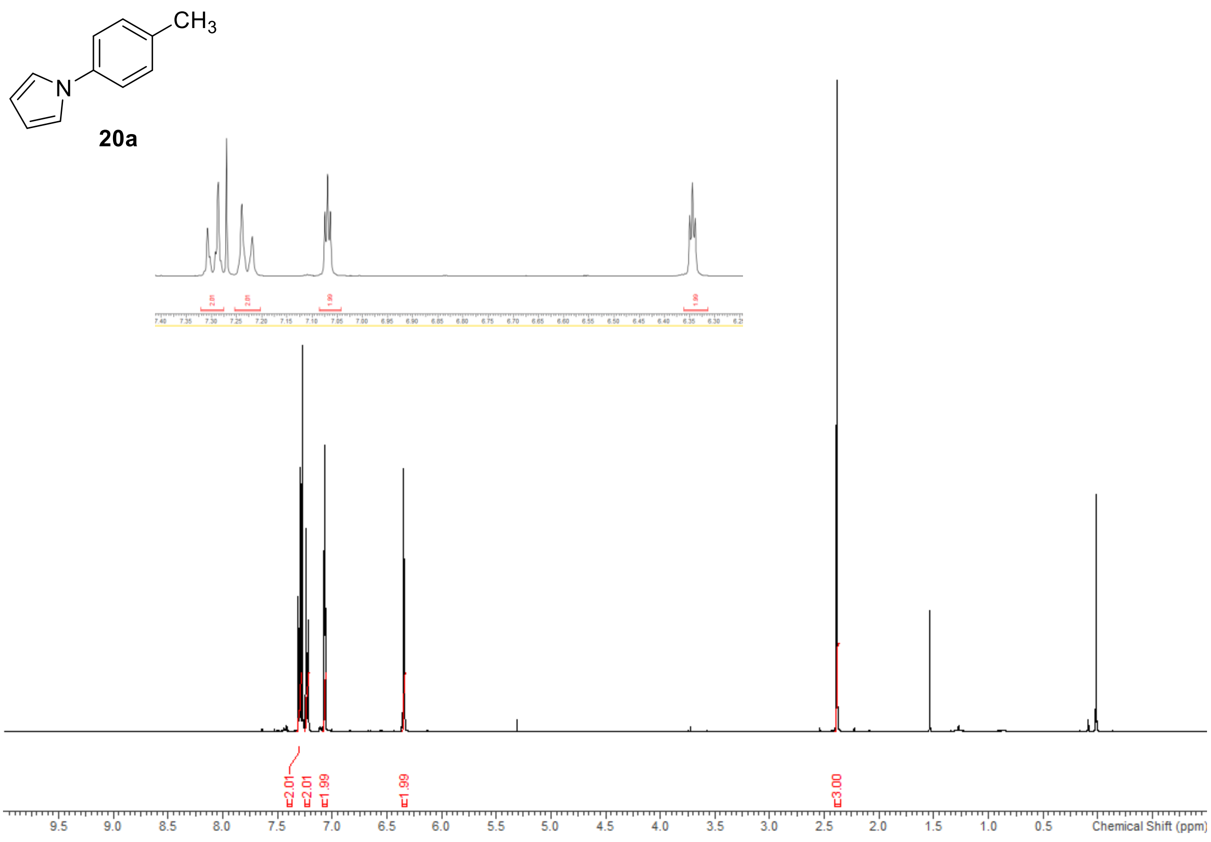

^{13}C NMR (100 MHz, $CDCl_3$) of **20a**

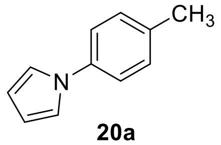

20a

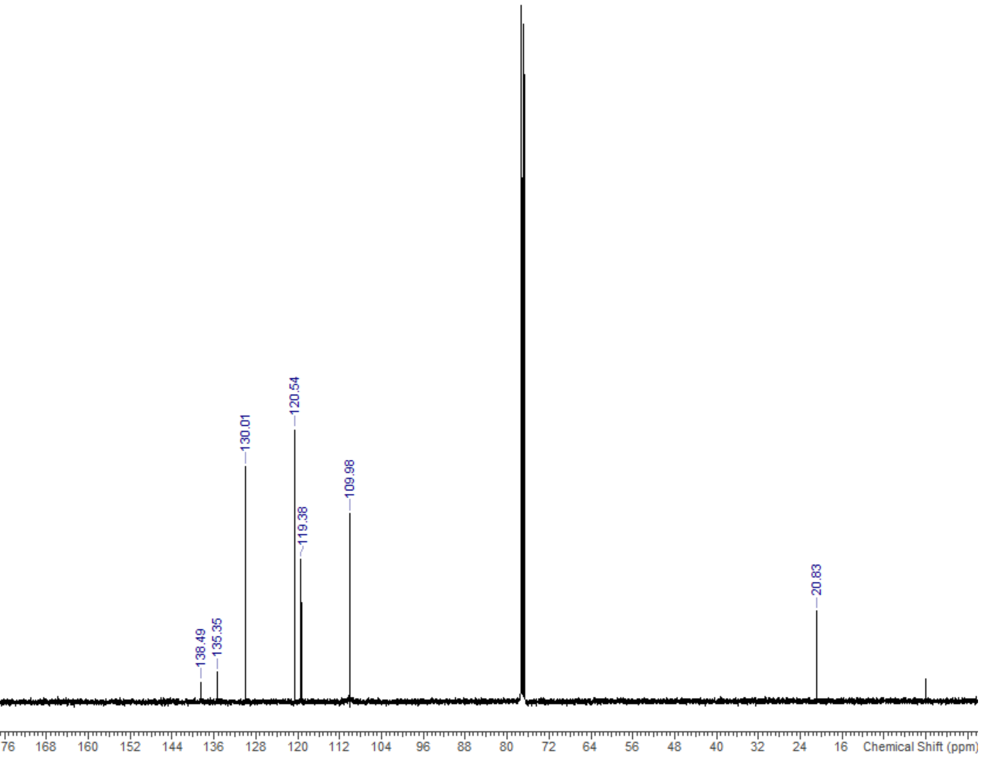

^{1}H NMR (400 MHz, $CDCl_3$) of **21a**

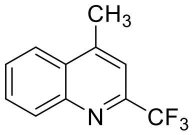

21a

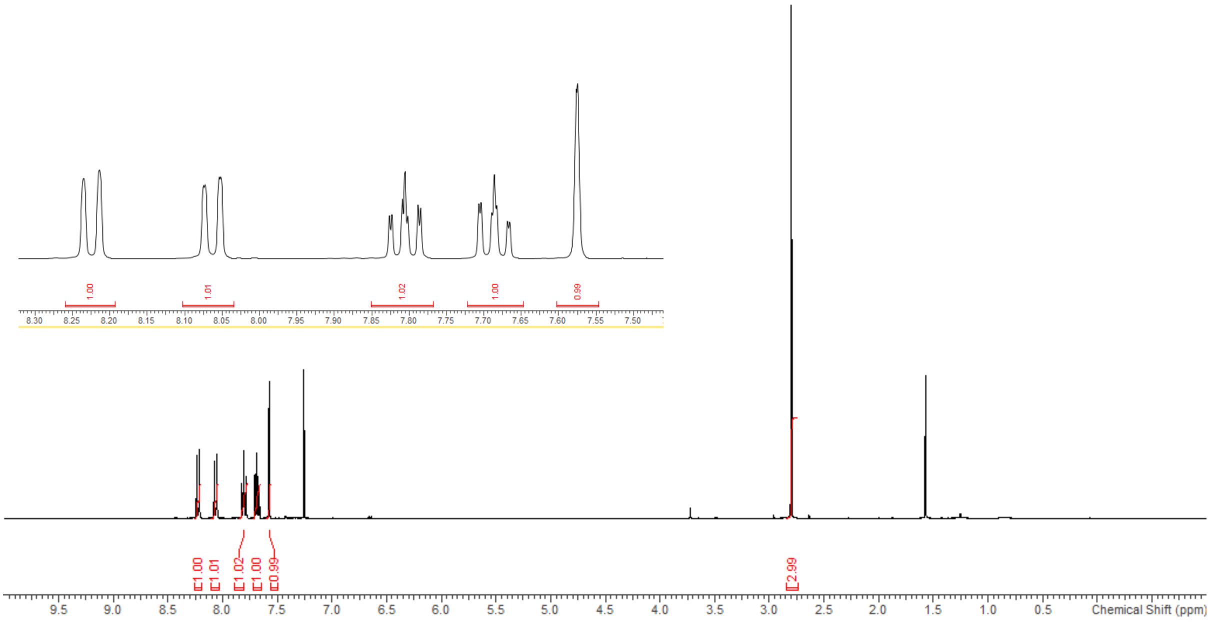

^{13}C NMR (100 MHz, CDCl$_3$) of **21a**

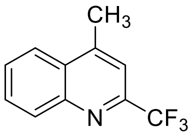

21a

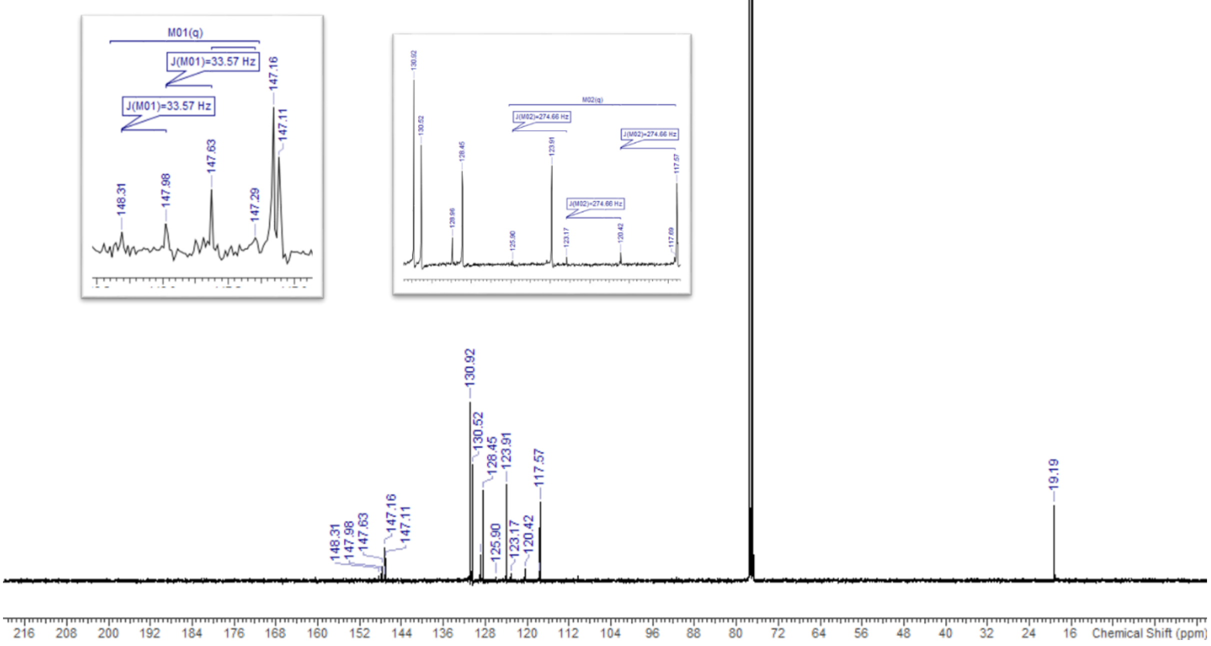

^{19}F NMR (376 MHz, CDCl$_3$) of **21a**

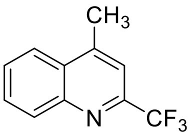

21a

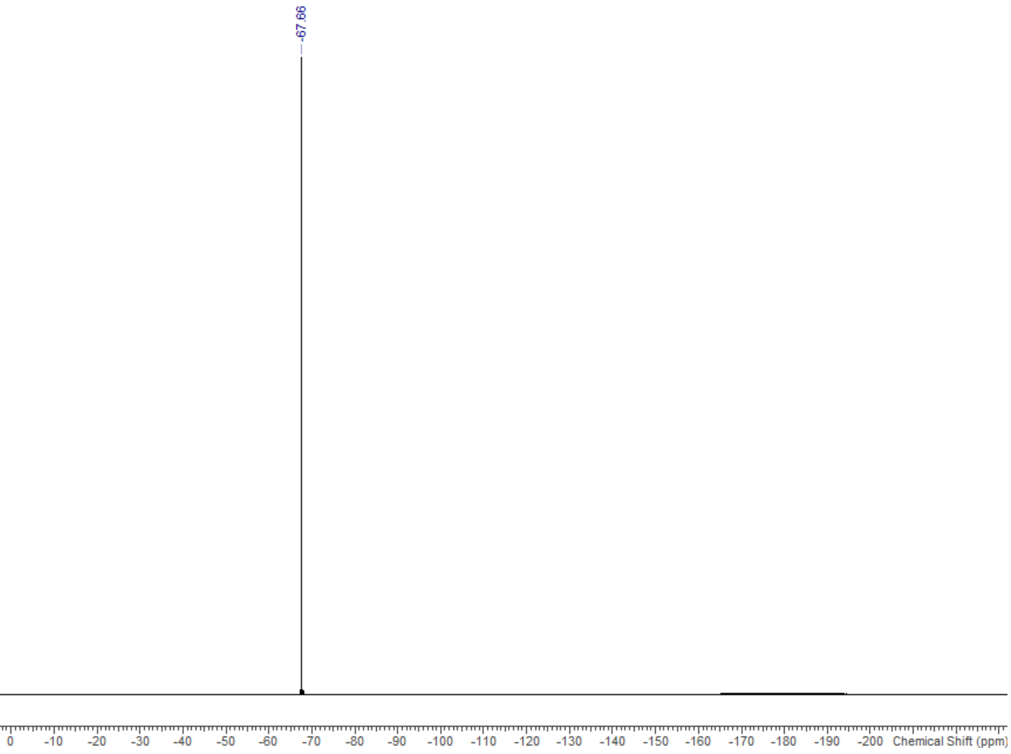

^{1}H NMR (400 MHz, $CDCl_3$) of **22a**

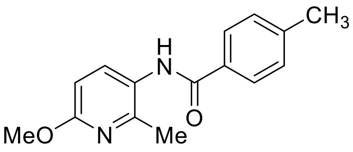

22a

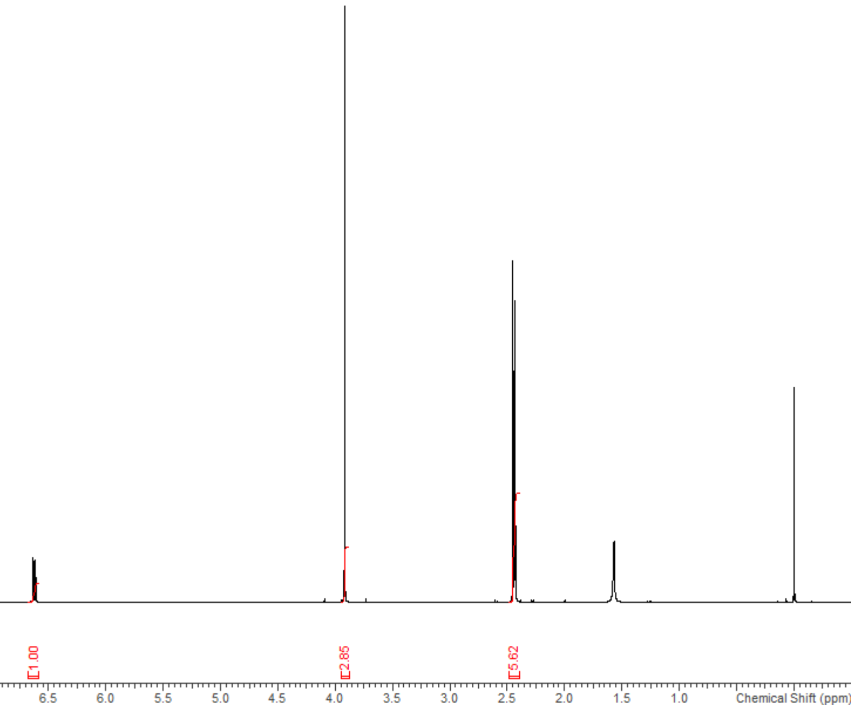

^{13}C NMR (100 MHz, $CDCl_3$) of **22a**

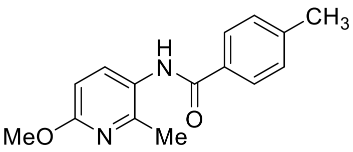

22a

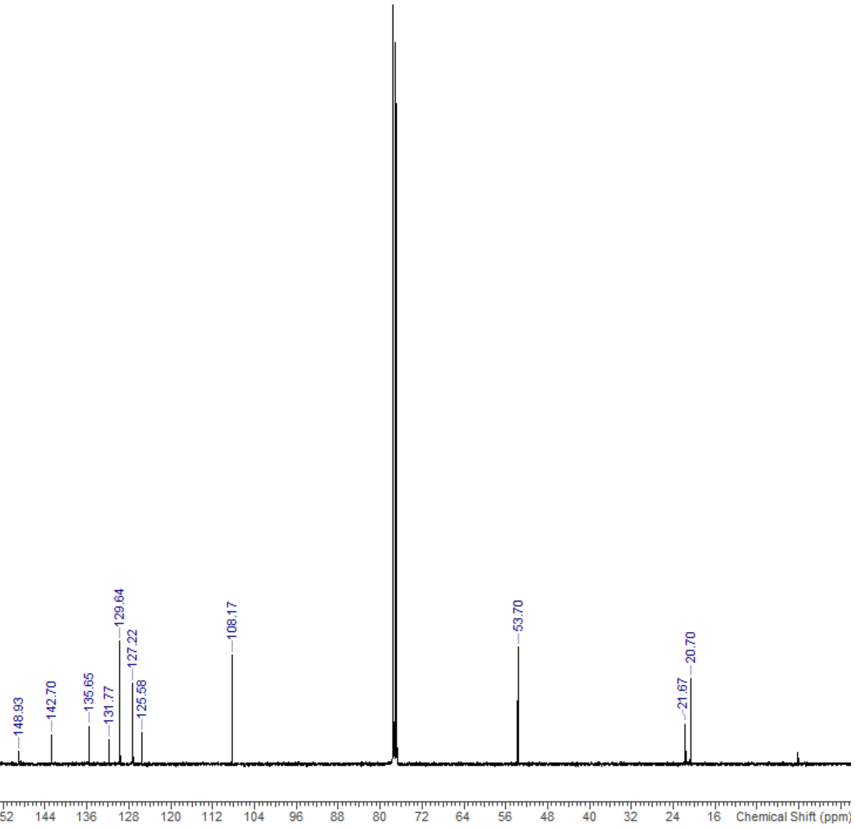

^{1}H NMR (400 MHz, $CDCl_3$) of **27**

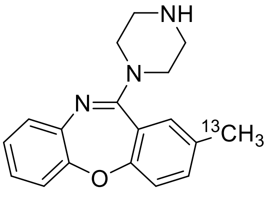

27

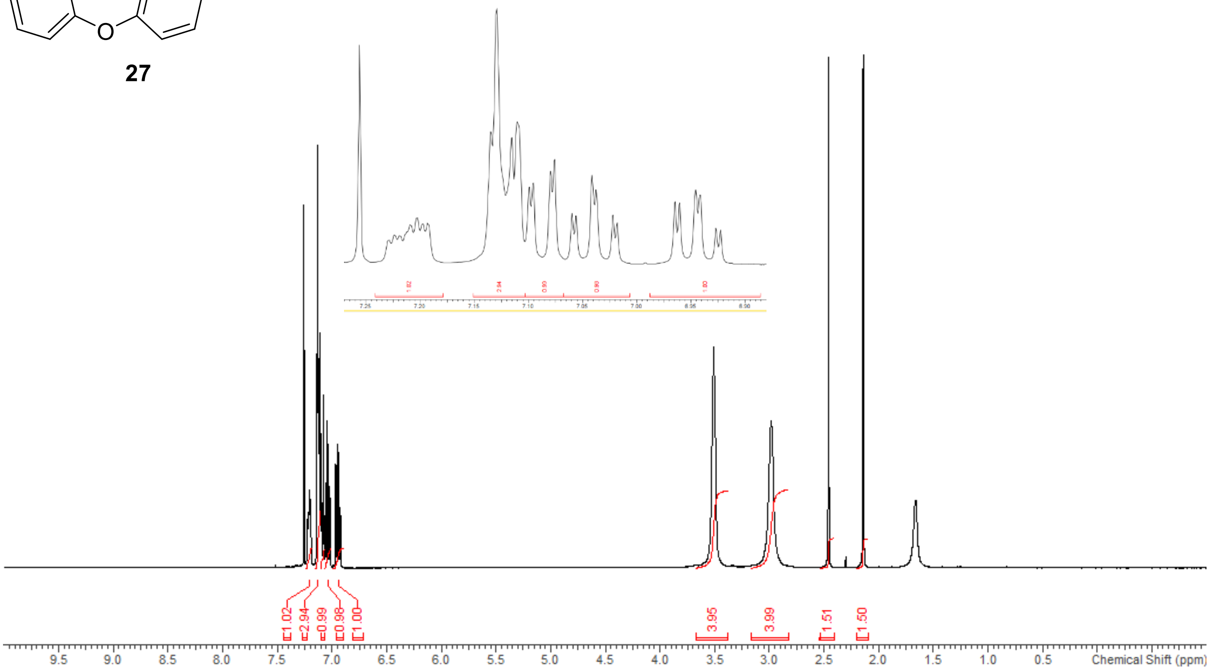

^{13}C NMR (100 MHz, $CDCl_3$) of **27**

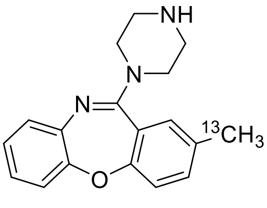

27

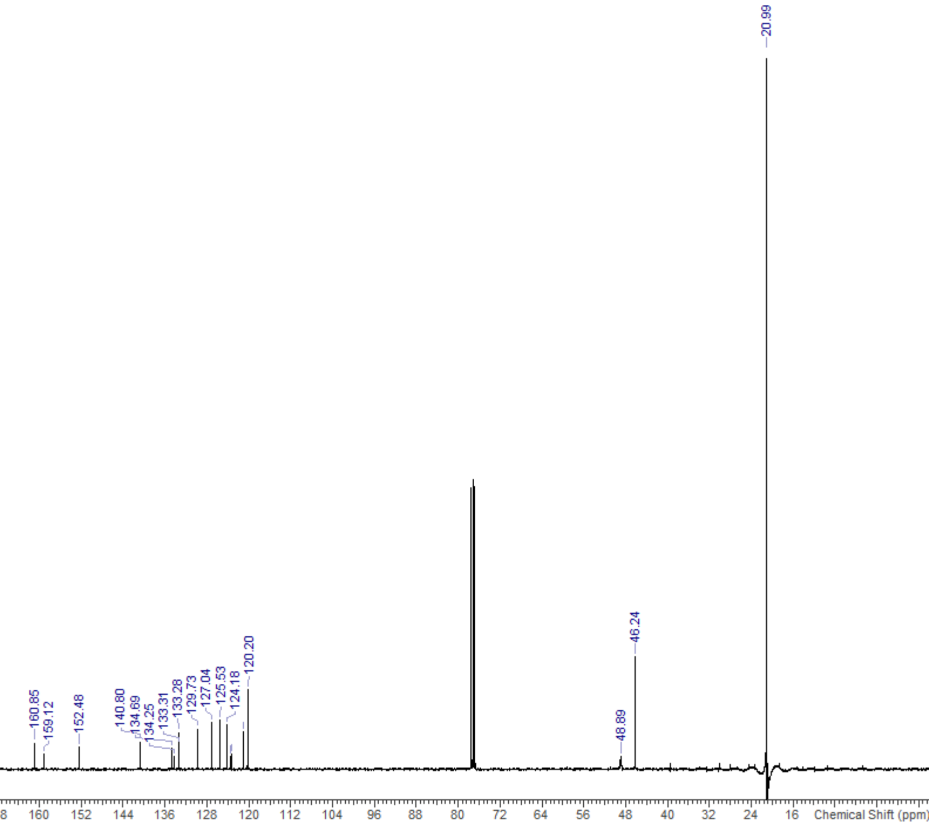

^{1}H NMR (400 MHz, CDCl3) of **28**

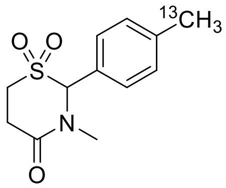

28

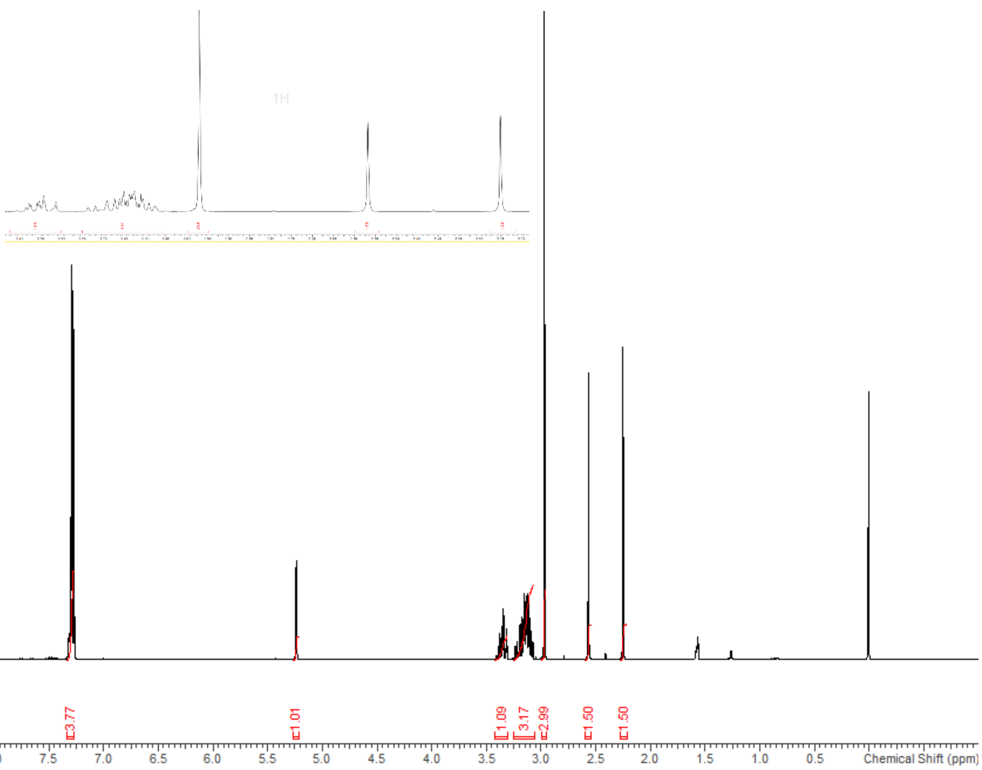

^{13}C NMR (100 MHz, CDCl3) of **28**

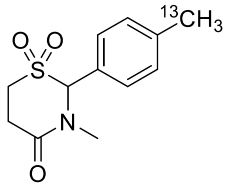

28

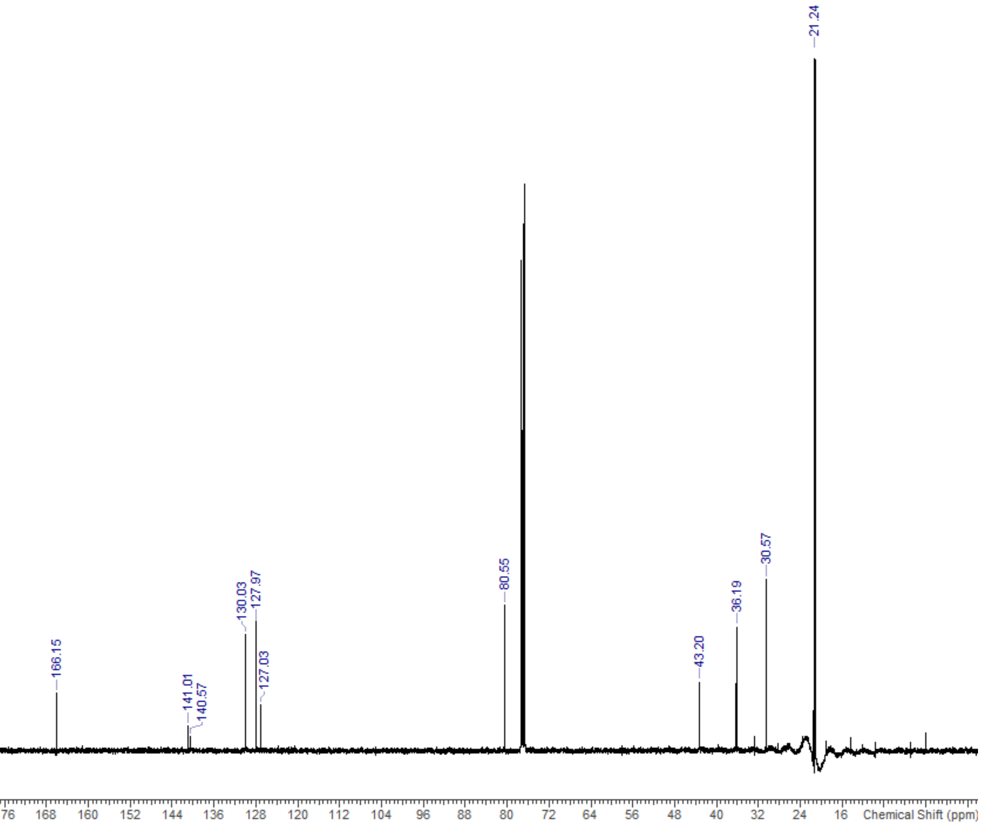

^{1}H NMR (400 MHz, $CDCl_3$) of **29**

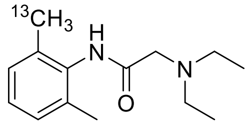

29

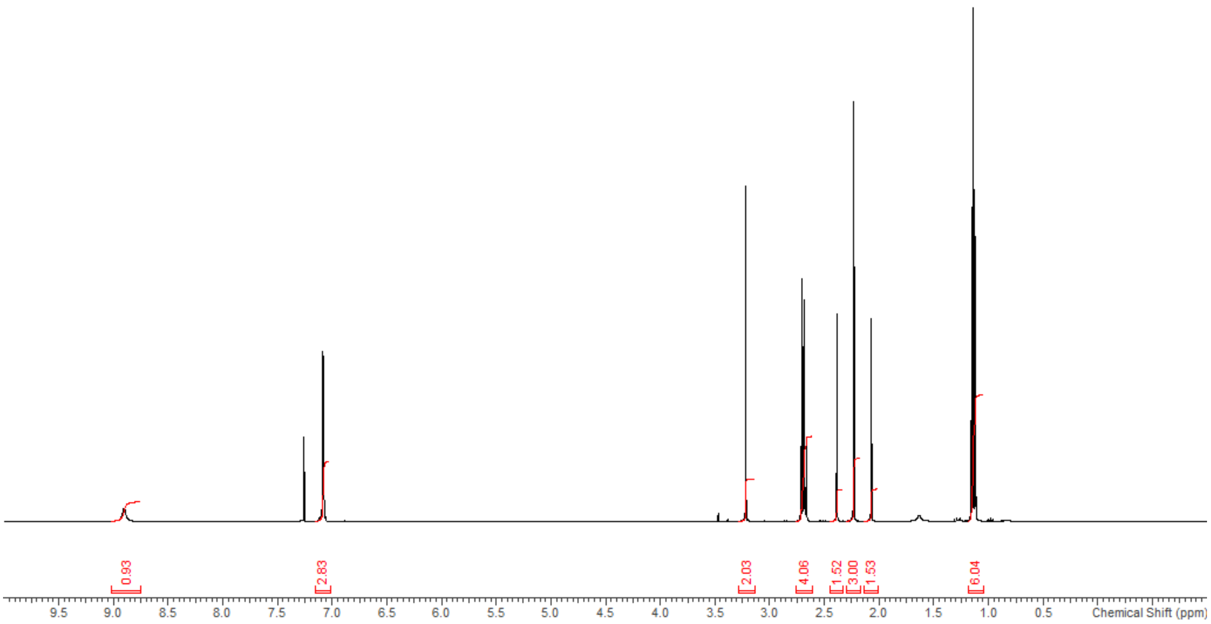

^{13}C NMR (100 MHz, $CDCl_3$) of **29**

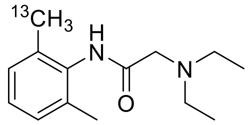

29

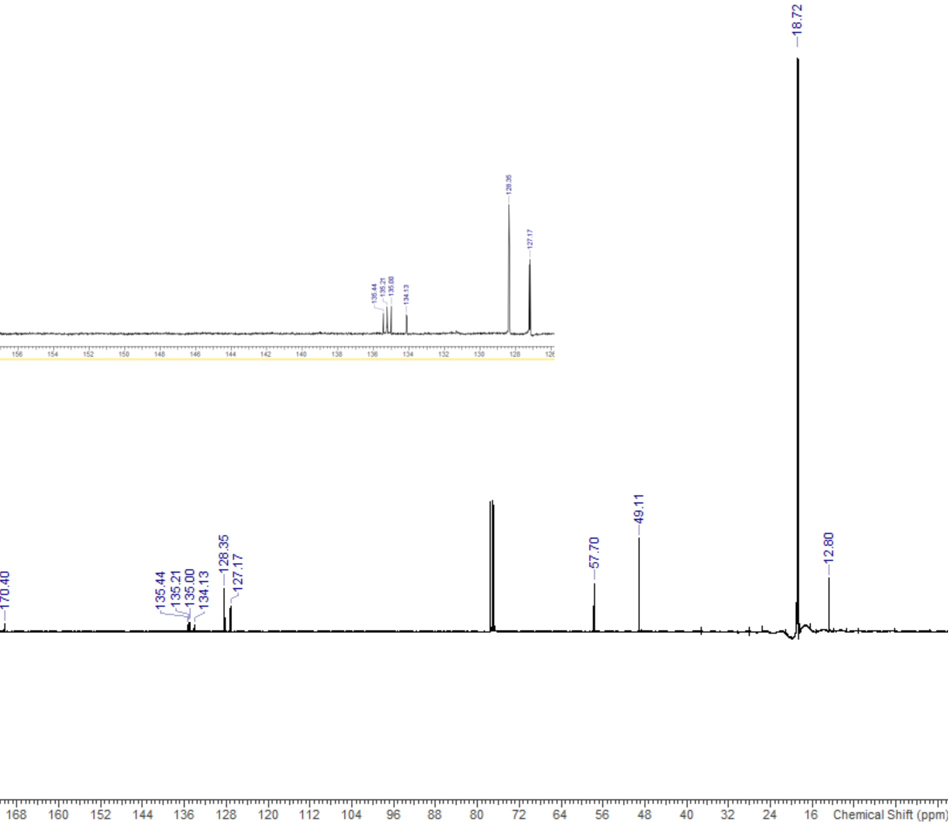

^{1}H NMR (400 MHz, $CDCl_3$) of **30**

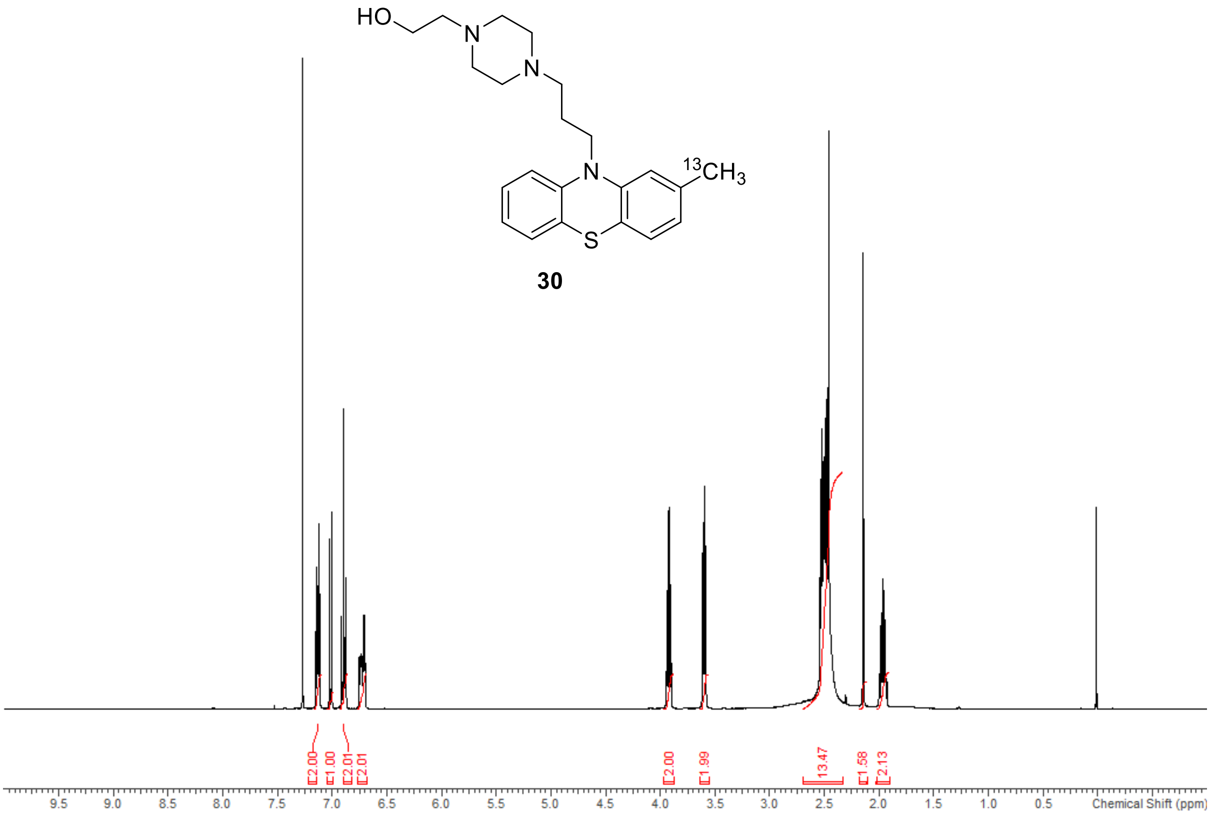

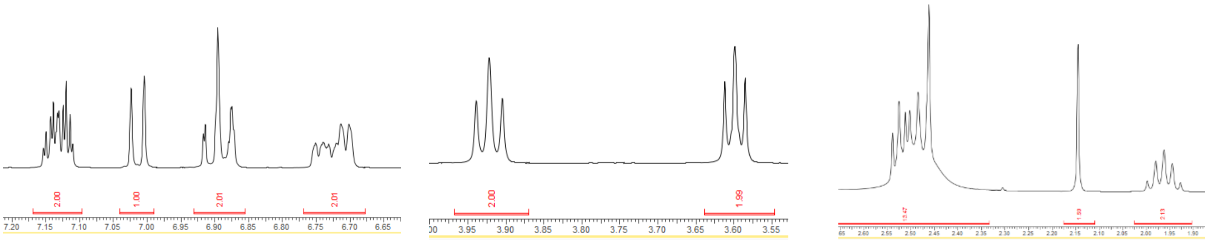

^{13}C NMR (100 MHz, $CDCl_3$) of **30**

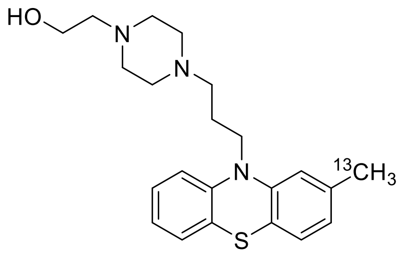

30

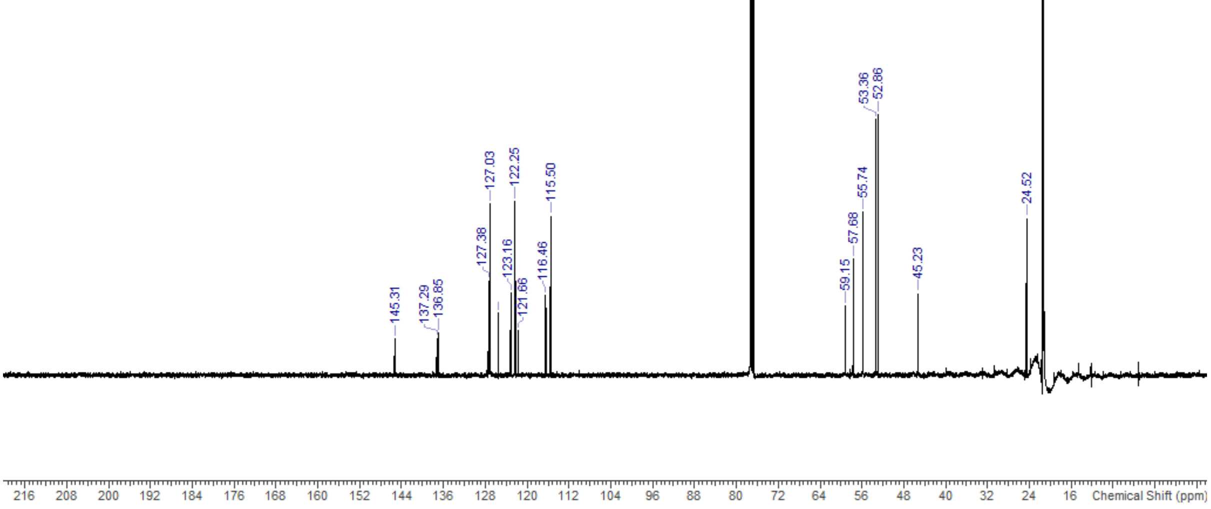

^{1}H NMR (400 MHz, $CDCl_3$) of **31**

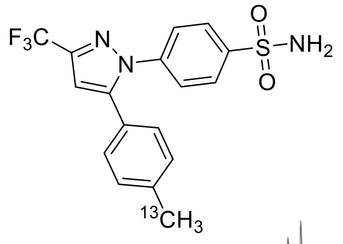

31

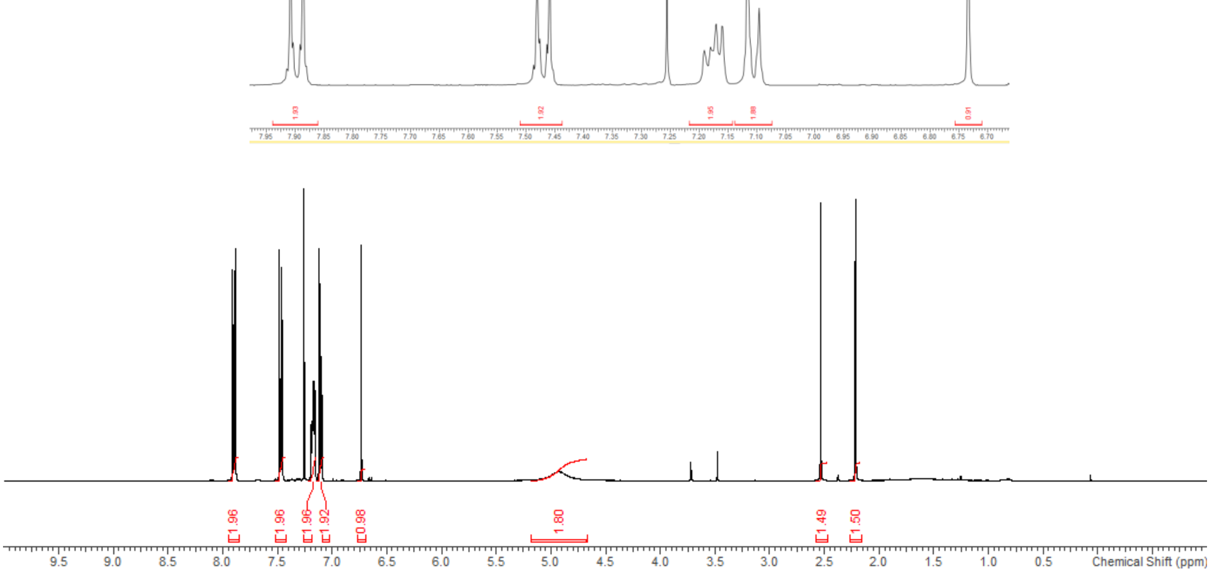

^{13}C NMR (100 MHz, $CDCl_3$) of **31**

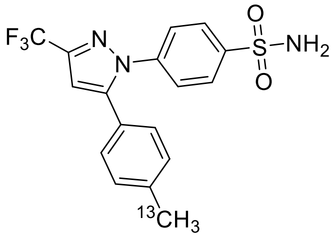

31

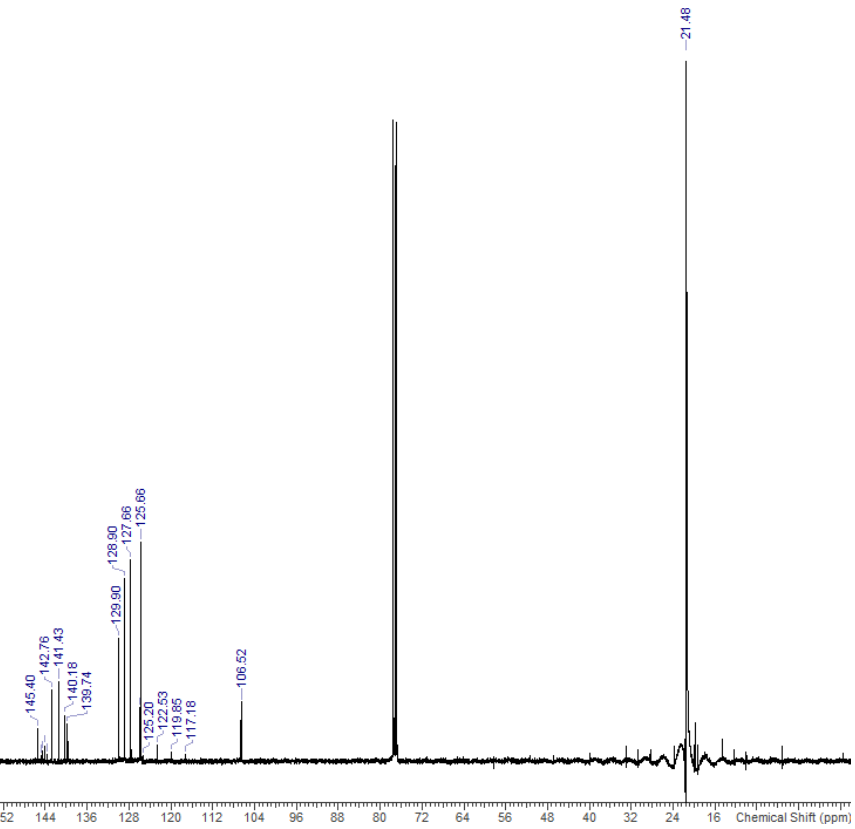

^{19}F NMR (376 MHz, $CDCl_3$) of **31**

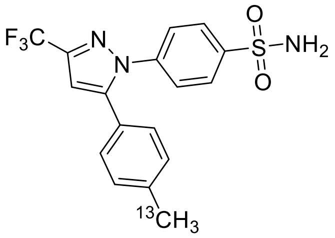

31

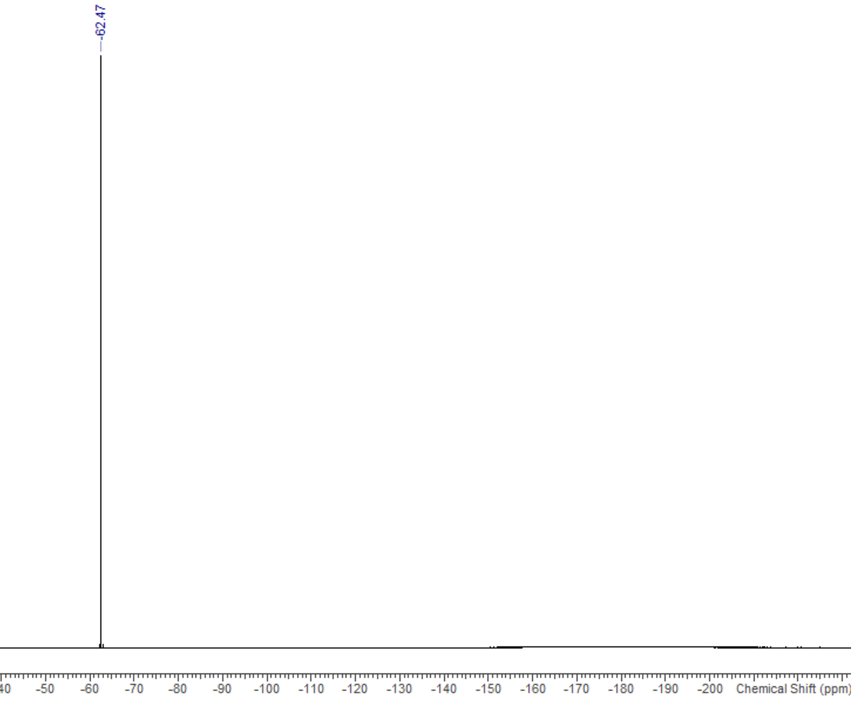

^{1}H NMR (400 MHz, DMSO-d_6) of **32**

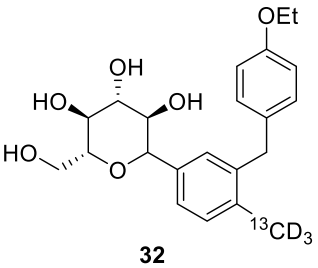

32

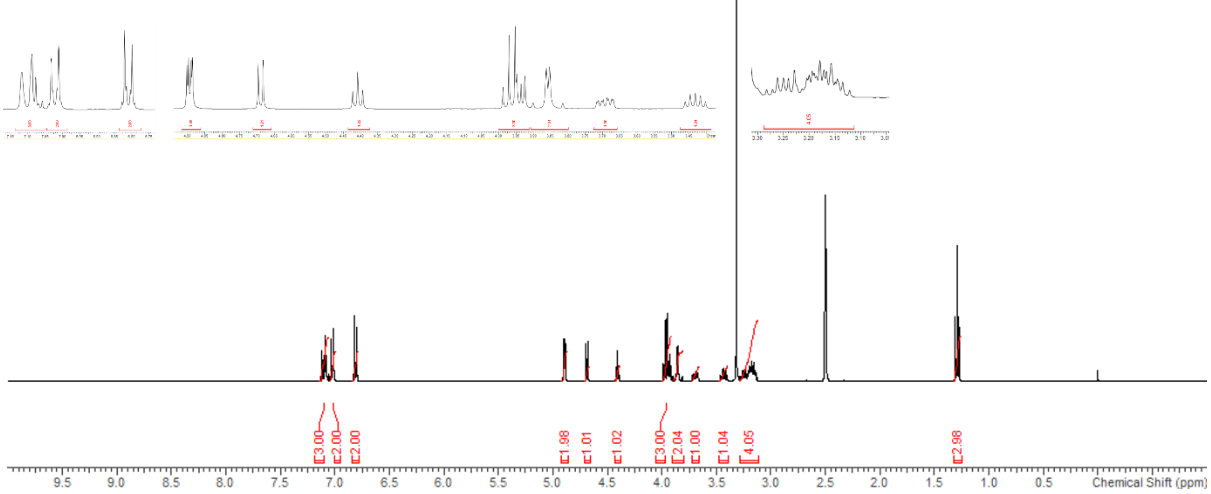

^{13}C NMR (100 MHz, CD_3OD) of **32**

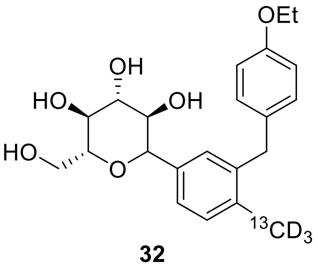

32

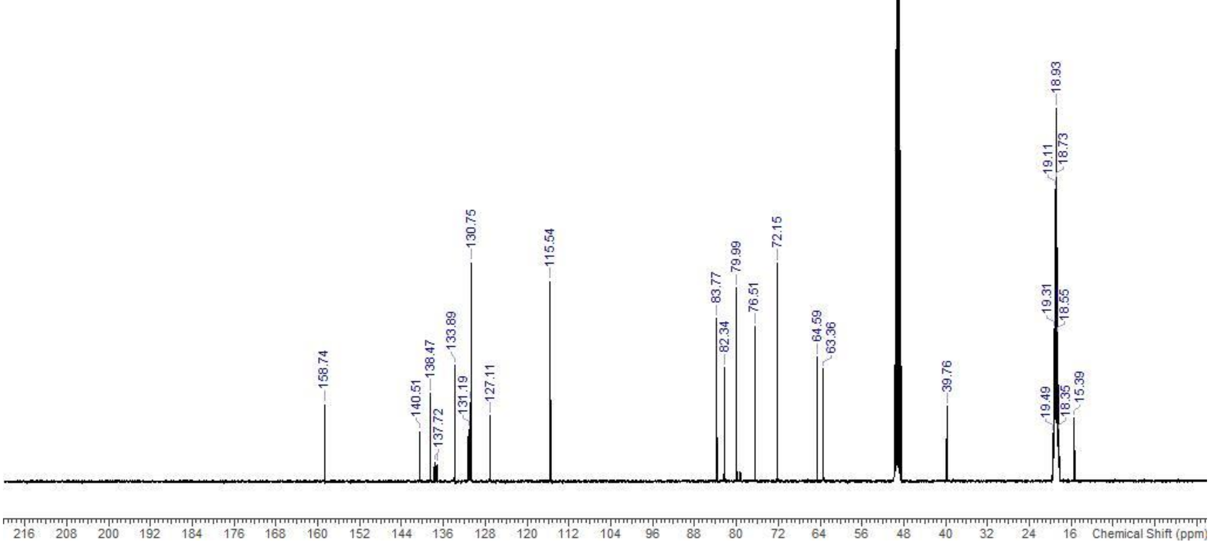

^{1}H NMR (400 MHz, $CDCl_3$) of **33**

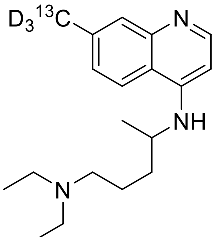

33

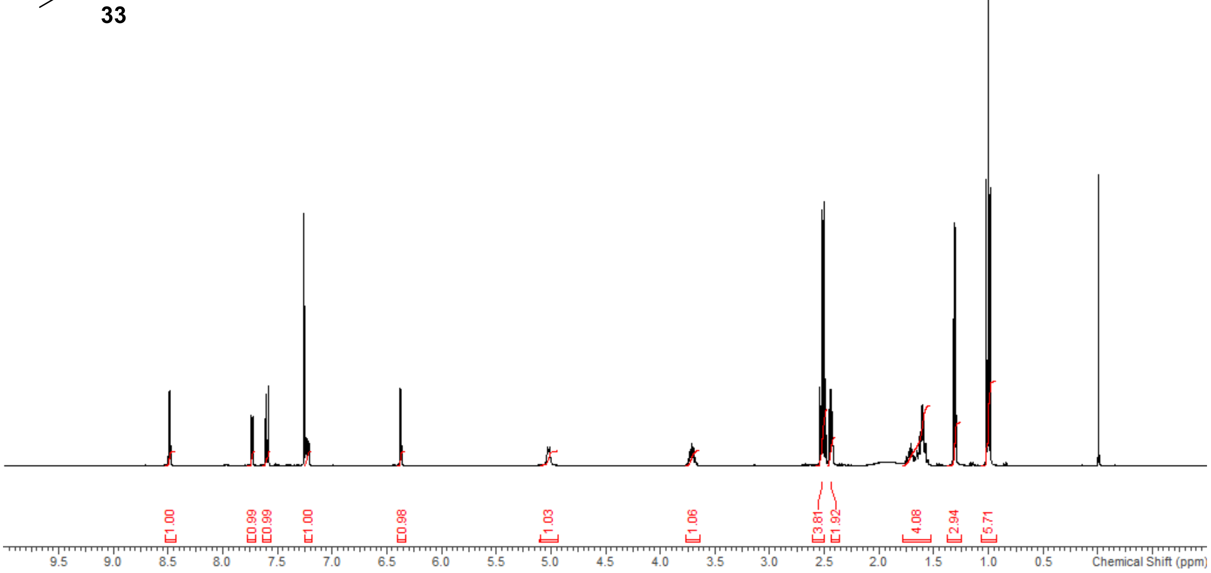

^{13}C NMR (100 MHz, $CDCl_3$) of **33**

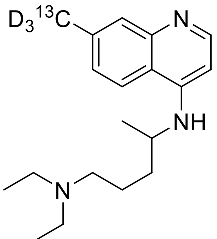

33

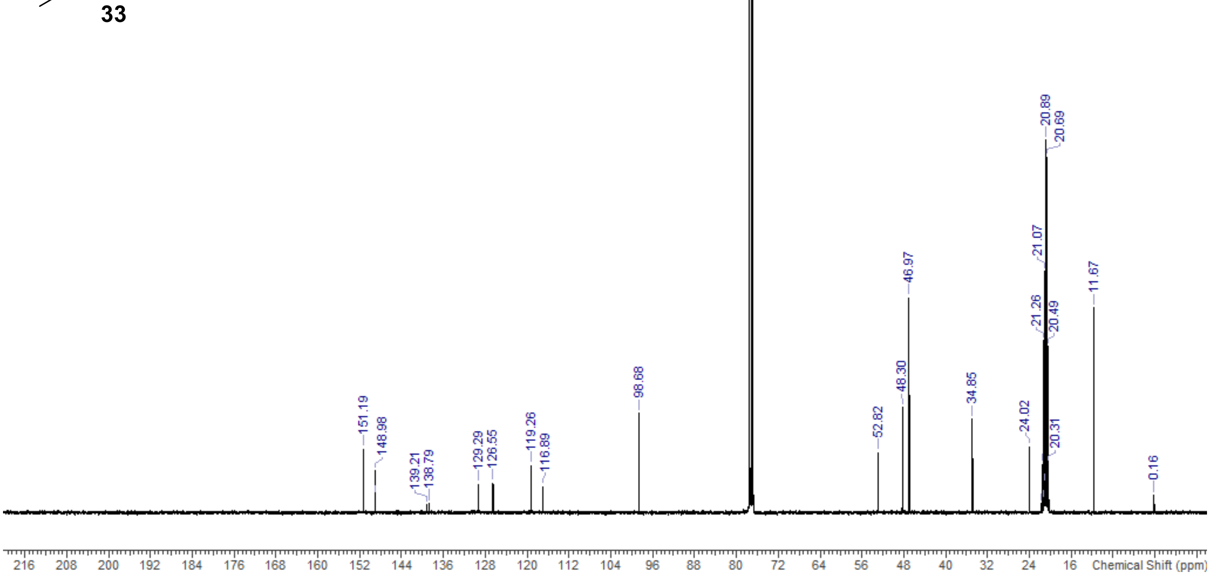

^{1}H NMR (400 MHz, $CDCl_3$) of **34**

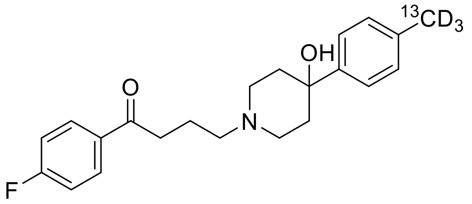

34

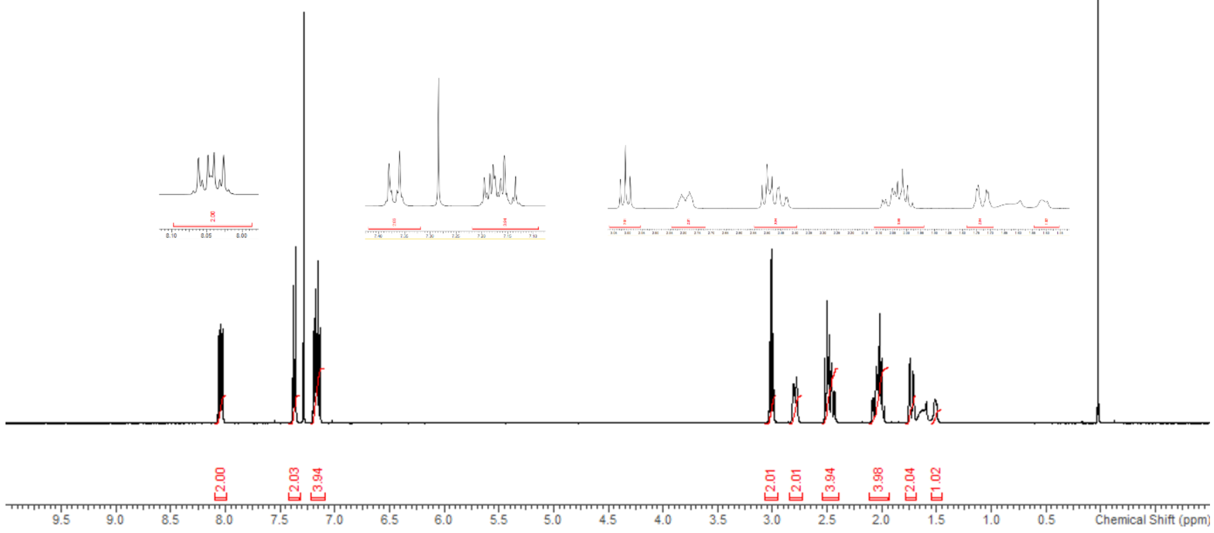

^{13}C NMR (100 MHz, $CDCl_3$) of **34**

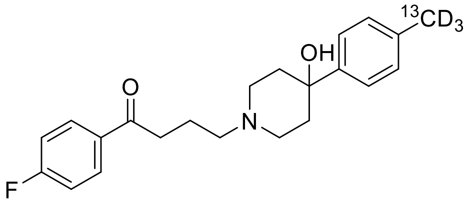

34

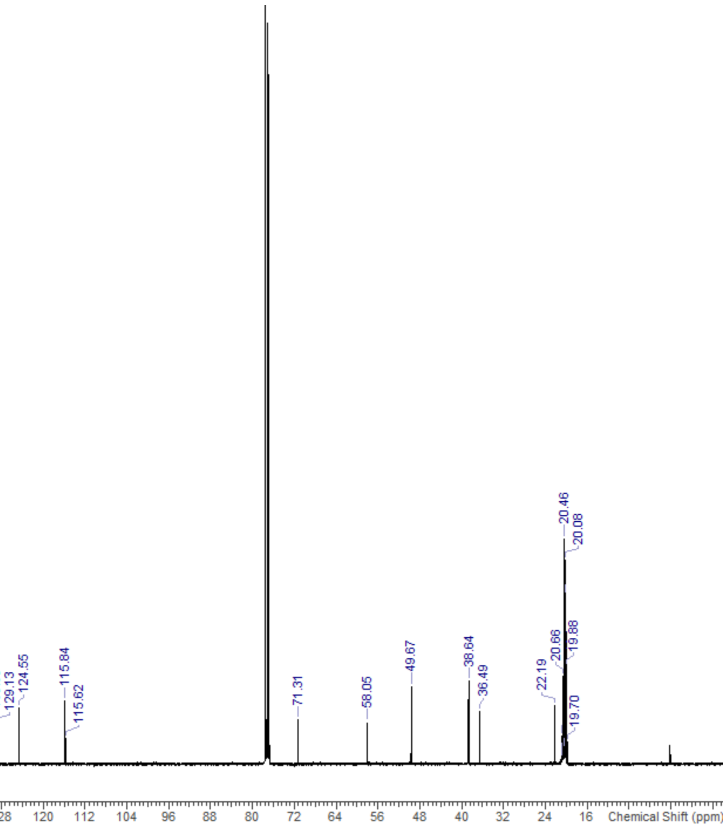

^{19}F NMR (376 MHz, $CDCl_3$) of **34**

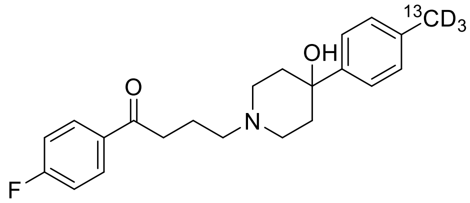

34

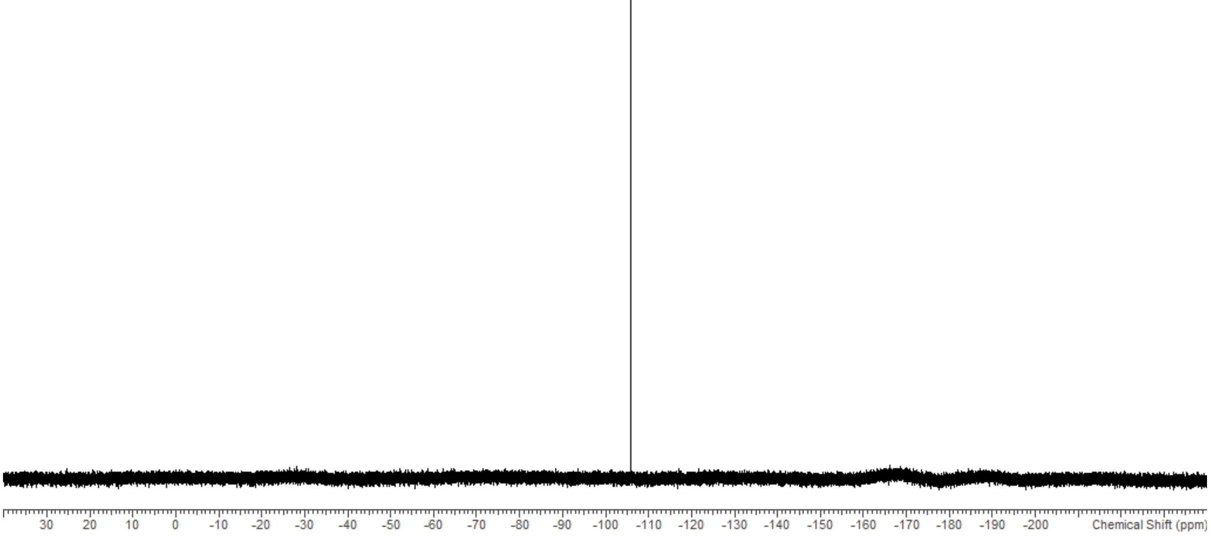

Supplement: Supplementary file 1 — cs3c02761_si_001.pdf [file cs3c02761_si_001.pdf]
